# Recent Progress on Polysaccharide-Based Hydrogels for Controlled Delivery of Therapeutic Biomolecules

**DOI:** 10.1021/acsbiomaterials.0c01784

**Published:** 2021-06-17

**Authors:** M. Isabel Rial-Hermida, Ana Rey-Rico, Barbara Blanco-Fernandez, Natalia Carballo-Pedrares, Eimear M. Byrne, João F. Mano

**Affiliations:** † Department of Chemistry, CICECO−Aveiro Institute of Materials, University of Aveiro 3810-193 Aveiro, Portugal; ‡ Cell Therapy and Regenerative Medicine Unit, Centro de Investigacións Científicas Avanzadas (CICA), Universidade da Coruña, 15071 A Coruña, Spain; § Institute for Bioengineering of Catalonia (IBEC), The Barcelona Institute of Science and Technology, 08028 Barcelona, Spain; ∥ CIBER en Bioingeniería, Biomateriales y Nanomedicina, CIBER-BBN, 28029 Madrid, Spain; ⊥ Wellcome-Wolfson Institute For Experimental Medicine, Queen’s University Belfast, 97 Lisburn Road, Belfast BT9 7BL, United Kingdom

**Keywords:** biotherapeutics, hydrogels, controlled delivery, polysaccharides, tissue engineering, stimuli-responsiveness

## Abstract

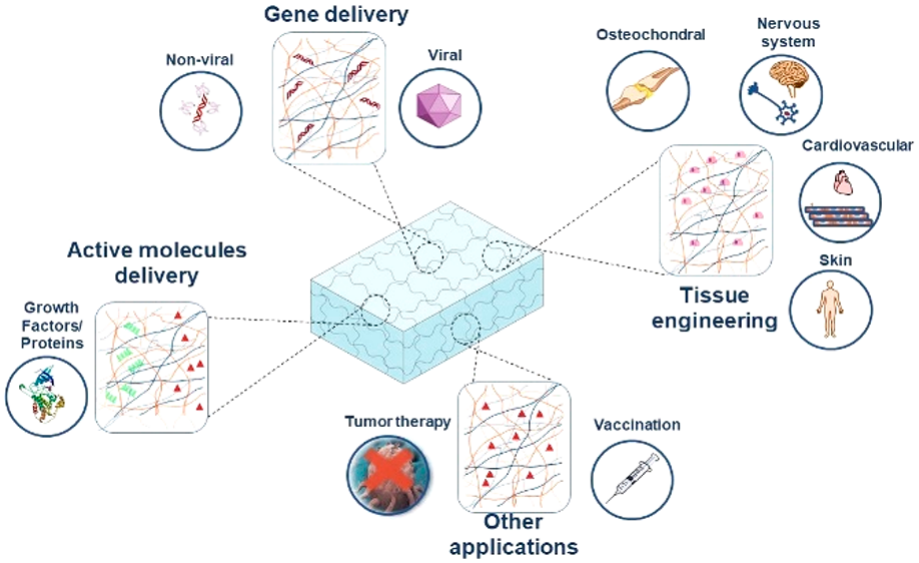

A plethora of applications using polysaccharides have been developed in recent years due to their availability as well as their frequent nontoxicity and biodegradability. These polymers are usually obtained from renewable sources or are byproducts of industrial processes, thus, their use is collaborative in waste management and shows promise for an enhanced sustainable circular economy. Regarding the development of novel delivery systems for biotherapeutics, the potential of polysaccharides is attractive for the previously mentioned properties and also for the possibility of chemical modification of their structures, their ability to form matrixes of diverse architectures and mechanical properties, as well as for their ability to maintain bioactivity following incorporation of the biomolecules into the matrix. Biotherapeutics, such as proteins, growth factors, gene vectors, enzymes, hormones, DNA/RNA, and antibodies are currently in use as major therapeutics in a wide range of pathologies. In the present review, we summarize recent progress in the development of polysaccharide-based hydrogels of diverse nature, alone or in combination with other polymers or drug delivery systems, which have been implemented in the delivery of biotherapeutics in the pharmaceutical and biomedical fields.

## 1 Introduction

Hydrogels based on biodegradable and bioabsorbable natural polymers have been widely used in drug delivery systems over the last 50 years. Hydrogels exhibit valuable properties and advantages, such as low toxicity or swelling.^1,2^ Over the past decade, these systems have attracted considerable attention for the development of therapeutic biomolecule delivery systems, including hormones, growth factors (GFs), gene vectors, or monoclonal antibodies. Hydrogels can slow down or even prevent the biodegradation of these biomolecules and/or sustain their release.^3−6^ It is important to note that some of the biopolymers forming part of hydrogels are sensitive to changes in the environment *per se* or upon chemical modifications (as pH, temperature, or ion concentration changes) and are susceptible to chemical tailoring for enhanced properties.^7^ Most polysaccharides are highly abundant, nontoxic, biodegradable, and easy to obtain from nature or byproducts of various industries, which means their repurposing assists in the development of adequate waste management and holds promise for the creation of a sustainable circular economy.^8^ For example, the sea has been explored as a rich source of polysaccharides which have potential for drug delivery applications.^9^ Such polysaccharides have specific properties and structures that are difficult to recapitulate via chemical synthesis,^10^ and they are usually used in the form of hydrogels, which recapitulate many structural and functional characteristics of living tissues.^11^

Delivery of biotherapeutics remains an enormous challenge due to their rapid degradation and metabolism once administrated by classical routes, which result in poor bioavailability.^12^ Currently, therapeutic biomolecules are receiving increased attention for their potential applications in clinical settings,^13,14^ including in the most recent diseases such as Covid-19,^15^ because of the high specificity for their target and, in some cases, their functional importance in physiological mechanisms.^3^ Preservation of the conformation of biomolecules is essential for the maintenance of their activity, particularly in the case of proteins or peptides. Therefore, natural processes of oxidation, deamination, or proteolysis phenomena should be avoided in their storage, transport, and final delivery as well as upon administration to ensure their integrity.^16^ Additionally, controlled and local release of proteins when and where required, may favor both the preservation of biomolecule’s activity and its safety in the cases where they may induce toxicity or immunological responses.^17^ Polysaccharides are excellent candidates as vehicles for therapeutic biomolecules, due to their easy release modulation and their capacity to maintain conformation and bioactivity of the biomolecule.

This review details important developments which have taken place in the past decade in terms of the use of polysaccharide-based hydrogels for the delivery of therapeutic biomolecules, including growth factors, nucleic acids, proteins, and enzymes. We highlight the most promising results obtained in this field and their vast potential for therapeutic use.

## 2 Formation of Polysaccharide-Based Hydrogels and Release Mechanisms

Polysaccharide-based hydrogels have been successfully used as delivery platforms in a broad range of fields, from tissue engineering to drug delivery. In the case of delivery of therapeutic biomolecules, a mild hydrogel cross-linking is usually required to guarantee their integrity and activity.

### 2.1 Cross-Linking of Polysaccharides Forming Hydrogels

Generally, we can classify hydrogels into physically and chemically cross-linked systems.^18^ Physical hydrogels are cross-linked through noncovalent bonds. The weak bonds within the polysaccharide chains usually make the cross-linking of these hydrogels reversible. Physical cross-links do not require the use of covalent cross-linking agents, and the hydrogel formation may occur in mild conditions, making these platforms promising systems for delivery of biomolecules because these conditions favor preservation of the structural and conformational integrity of the biomolecules.^19^ Typically, polysaccharide-based hydrogels are physically cross-linked by means of electrostatic interactions,^20^ hydrophobic interactions,^21^ ionic cross-linking supported by multivalent ions,^22^ van der Waals forces as hydrogen bonds,^23^ or host–guest complexes.^24^ Below, the most common methods are briefly explained.

Cross-linking by multivalent ions is based on the principle of gelling a polyelectrolyte solution followed by the addition of multivalent ions of opposite charge, or even other charged structures such as micro- or nanoparticles.^25^ Hydrogen bonding is another common approach for physical cross-linking polysaccharides chains. For example, *in situ* or shear thinning hydrogels and self-healing systems usually gel by means of physical cross-linking.^2^

Covalently cross-linked networks are commonly prepared by the union of small multifunctional molecules such as monomers, photoreactive groups, or oligomers through strong and irreversible bonds. Functional groups are included in the polysaccharide chains, naturally or by grafting to them, and typically have a key role in the covalent polymerization process.^3^ Typical chemical groups of polysaccharides involved in covalent cross-linking are carboxyl and amine groups, using carbodiimide chemistry.^26^ Moreover, polysaccharides can be easily functionalized by the addition of reactive groups such as thiols, alkenes, or acrylates due the high content in hydroxyl, amine, or carboxylic groups, which are covalently cross-linked.^3^ Several examples of these will be given in section 3.

Physically and chemically cross-linked hydrogels are able to incorporate therapeutic biomolecules by means of weak interactions, by adsorption in the structure, or by cleavable bonds.^3,4,27,28^ We will explore the different mechanisms of delivery of immobilized biotherapeutics from such cross-linked structures.

### 2.2 Mechanism of Delivery of Therapeutic Biomolecules

**Figure 1:**
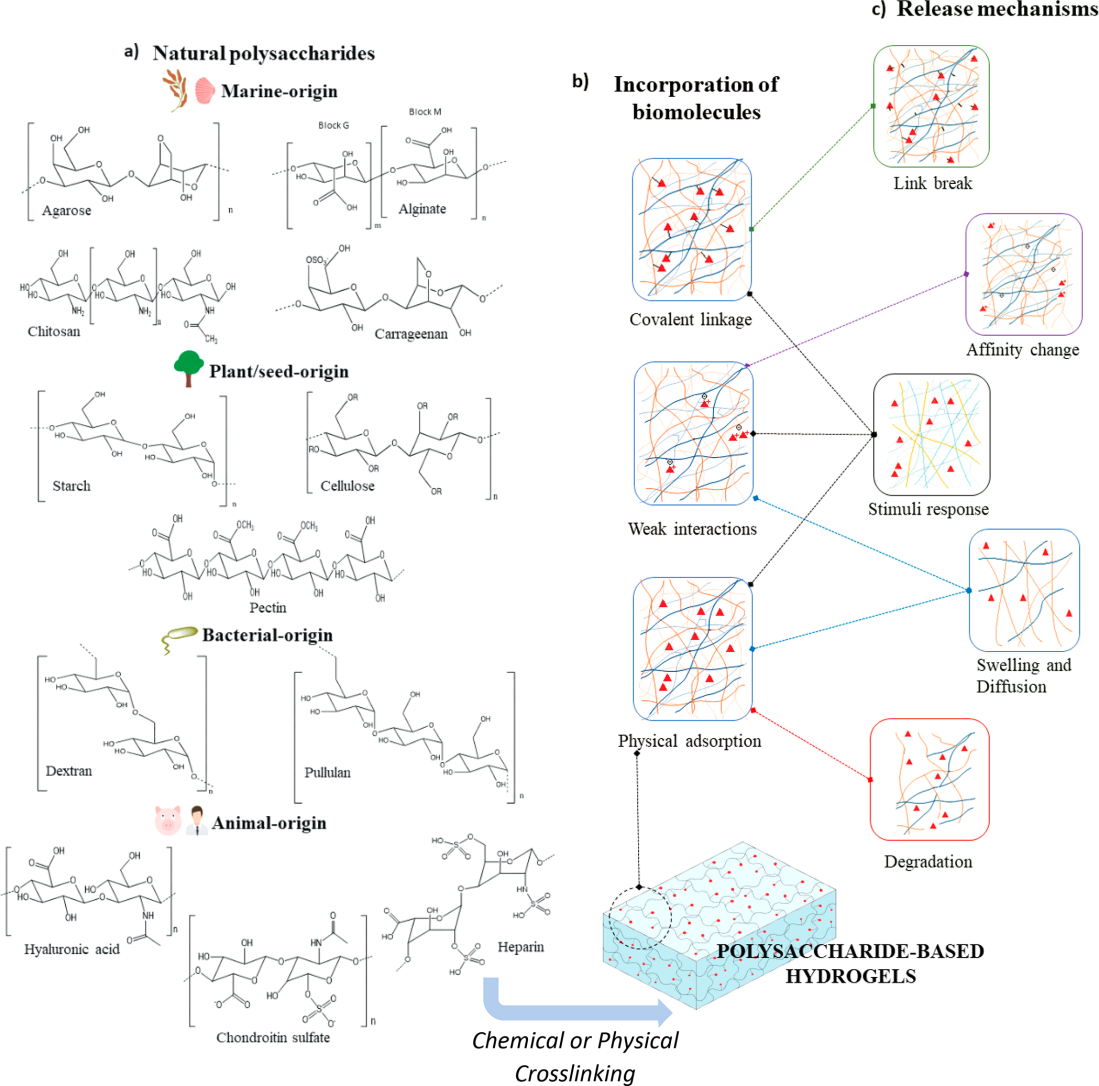
(a) Examples of natural polysaccharides that could produce chemically or physically cross-linked hydrogels, (b) methods to incorporate biomolecules into the obtained matrix, and (c) different mechanisms of release from the polysaccharide-based hydrogels.

In a classic approach to biomolecule delivery, therapeutic biomolecules are intravenously, subcutaneously, or intramuscularly administered; thus, the physicochemical properties of the biomolecules are the principal factors involved in their pharmacokinetics.^3^ The implementation of hydrogels could completely change this situation. Some of the mechanisms of biomolecule release from polysaccharide hydrogels are summarized in Figure 1.

- Controlled delivery by diffusion and swelling: In this case, the release of the therapeutic biomolecules is controlled by the mesh size of the macromolecular networks or by local porosity in the hydrogel structure, which generally permits the diffusion of liquid and small solute.^27^ This type of release is directly interconnected with the swelling of the matrix. Both phenomena can usually be taking part in the same delivery process. During the swelling process, hydrogels can absorb large quantities of water without dissolving through the extension of the polysaccharide chains, while still maintaining their interactions to protect the structure of the system. During this matrix reorganization, biomolecules can be delivered to the release media.^29^
- Stimuli-responsive controlled delivery: Some polysaccharides can react to changes in the environment. Moreover, they can be easily rendered functional with stimuli-sensitive motifs. Cross-linked polysaccharides can effortlessly provide networks that are sensitive to a range of internal or external stimuli, such as ion concentration changes,^30^ light wavelength,^31^ variations in pH,^32^ intensity of the magnetic^33^ or electric field,^34^ temperature,^35^ REDOX potential,^36^ or the presence of several biomolecules,^37^ leading to the possibility of the release of the biomolecules by controlling those stimuli.^38,39^ These systems therefore hold great potential for the development of switch on–off drug release systems.^40^
- Controlled delivery by degradation: As a consequence of physiological processes, biodegradation of the hydrogel is a natural approach for the delivery of entrapped biomolecules. The bioerosion of polysaccharides can control the release of biomolecules when the degradation speed is higher than the active agent diffusion. We can also take advantage of this process by applying, for example, a specific enzyme to digest the polysaccharide chains.^41^
- Affinity-based delivery: This mechanism employs the interactions between the biotherapeutic agent and the delivery system. These interactions can be useful bilaterally, in both incorporation and release of active agents. In these cases, the release can be tuned by the strength of the affinity interactions, the concentration of the binding ligand, the constant of dissociation of the formed complex, and by the size and geometry of the hydrogel.^42^ Ligand–protein binding in physiological conditions is a clear example of this. Moreover, molecular imprinted hydrogels or cyclodextrin-based delivery benefit from these interactions.^28^
- Link breaking-controlled delivery: When a biomolecule is incorporated into the matrix by a covalent union, the hydrolysis (or other bond-breaking action) of the bond is necessary to release the therapeutic molecule to the physiological media. These systems are designed for delivering the biomolecule when specific conditions in the media are present. For example, the increase of the pH when a bacterial infection occurs can be used for the hydrolysis of the link, and then, the active compound is released before the complete bacterial colonization happens.^43^

## 3 Controlled Delivery of Therapeutic Biomolecules from Polysaccharide-Based Hydrogels

A large variety of hydrogels have been explored for the fixation and delivery of biomolecules. In this section, such systems will be organized by the source from where the polysaccharides are obtained. As some polysaccharides are acquired from several sources, the classification exclusively attends to where it is obtained in greater quantity.

### 3.1 Marine-Origin Polysaccharide-Based Hydrogels

#### 3.1.1 Alginate-Based Hydrogels

Alginate (ALG) is an anionic linear polysaccharide formed by blocks of consecutive or alternated β-d-mannuronic acid (M) and α-l-guluronic (G) units bonded by (1 → 4) linkages. ALG can be obtained from several sources, such as marine brown algae or bacteria, that differ in M/G content, block length, molecular weight, and consequently, in physicochemical properties.^44^ ALG hydrogels are generally fabricated by ionic gelation using divalent and trivalent cations (i.e., Ca^2+^). The mechanical properties of ALG gels can be modulated by increasing the molecular weight or the G-block length of the biopolymer, as G blocks are responsible for the coordination with divalent cations, which enable the coordination of adjacent chains, which actively form the gels (egg-box model). This increase in stiffness is responsible for modulating the drug release rate as well as control the stability of the gels.^45^ Some properties of ALG hydrogels, such as their mucoadhesivity, biodegradability, or nontoxicity, have motivated their use in the field of tissue engineering and drug delivery, such as wound healing, bioinks for 3D printing, *in vitro* models, or delivery of antitumoral drugs.^46^

The most used cross-linker for preparation of ALG hydrogels is Ca^2+^, although other ions can also be used. Ca^2+^ cross-linked hydrogels have extensively been used for the encapsulation of GFs or antibodies.^47,48^ For example, the monoclonal antibody bevacizumab has been encapsulated in ALG hydrogels for the antivascular endothelial growth factor (VEGF) activity in cancer therapy.^49^ The positive charge on the antibody can ionically interact with the negative charged ALG at physiological pH without affecting the integrity of the therapeutic. These type of platforms show a slow sustained and pH sensitive release of antibodies,^50^ leading to a reduction in the tumor size in animal models.^49^ Raimondo and colleagues developed ALG hydrogels for dual release of VEGF and insulin-like growth factor-1 (IGF-1). When implanted in a rodent model of sciatic nerve ligation and neurorrhaphy, these hydrogels were able to promote functional reinnervation.^51^ Pulsatile release of the payloads could be successfully achieved by the application of a ultrasonic stimulus.^52^ Ca^2+^ cross-linked hydrogels can selectively release their payloads in response to this type of stimuli and self-heal when the stimuli stops due to Ca^2+^ ion recross-linking the polymer network.^53^ Therefore, this property enables an on-demand delivery of macromolecules such as GFs.^52^

In order to mimic the heparin structure and increase GF binding properties, Park and colleagues modified ALG by incorporating sulfate groups. A 3D bioprinting approach was applied to combine bone morphogenetic protein 2 (BMP-2) and the hydrogel to sustain release for more than 10 days.^54^

ALG has also been combined with other polymers, such as hyaluronic acid (HA),^55^ collagen,^56^*N*-carboxymethyl chitosan,^57^ tragacanth gum,^58^ whey protein,^59^ and cellulose^60^ and then cross-linked with Ca^2+^ to obtain hydrogels with potential applications in different tissue engineering approaches. For example, ALG/HA hydrogels were able to sustain the release of basic fibroblasts growth factor (bFGF) for more than a month, and when injected into geriatric laryngeal muscles of rats, a tissue rejuvenation was observed.^61^ pDNA encoding bFGF complexed to polyethylene glycol/polyethylenimine (PEG/PEI) was loaded in hydrogels made of ALG/HA with polycaprolactone microparticles. They were able to sustain the release of the pDNA from 20 to 45 days, enough for a long-term bFGF transgene expression in fibroblasts. When injected into the vocal fold of rabbits with laryngeal nerve denervation, an unprecedented recovery of vocal function was achieved.^62^

ALG can be combined with self-assembled peptides to render the hydrogel biologically active, without the need for GFs or other bioactive proteins. For example, naphthaleneacetic-Gly-Phe-Phe-Tyr-Gly-Arg-Gly-Asp-His-His (Pept-1) was combined with alginate/Ca^2+^ hydrogels to develop a new wound dressing that accelerated wound closure through platelet activation.^63^

Rather than cross-linking with ions, ALG can also be cross-linked with polycationic polymers by ionic complexation, such as with chitosan forming polyelectrolyte complex (PEC). Chitosan/ALG PEC are effective carriers for the delivery of antibodies, genes, or other biomolecules.^64,65^ For example, anti-VEGF antibodies can be released up to 30 days after injection using such a kind of platform.^66^

**Figure 2:**
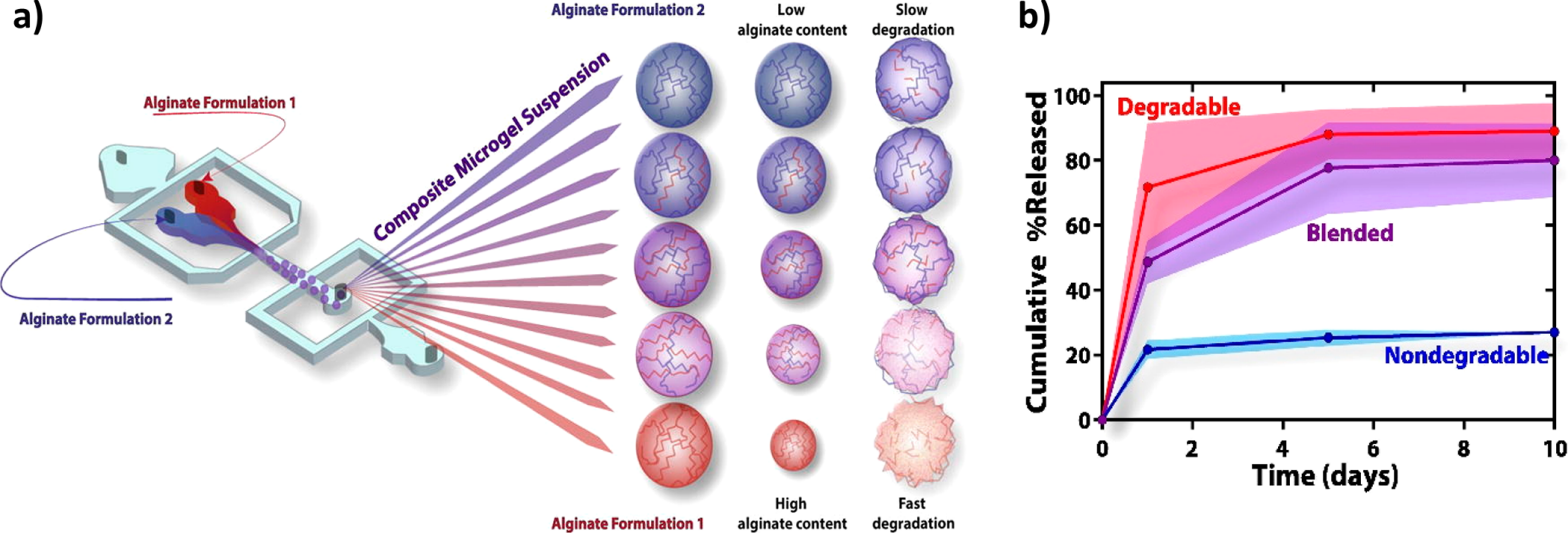
Several methods were followed to set up ALG novel systems, for example, (a) ALG microgels were prepared applying an on-chip polymer blending, by mixing two formulations of ALG differing in the molecular weight, concentration, or oxidation state; (b) different ALG formulations with different alginate oxidation state were synthesized to establish a relationship between ALG degradation rate and lentivectors delivery, showing that the presence of oxidated alginates in the microgels enhanced the delivery of the payloads due to the hydrolysis promotion of the hydrogel. Reproduced and adapted with permission from ref (69). Copyright 2018, Elsevier.

Partial oxidation of ALG enables an increase in the degradation of the polysaccharide by hydrolysis, without affecting the ionic cross-linking capabilities nor the toxicity.^67^ The subsequent reduction in degradation time may be beneficial for tissue regeneration, ocular delivery, or gene delivery.^67,68^ Priddy and colleagues demonstrated that the inclusion of BMP-2 in oxidized ALG hydrogels accelerated the release of this GF when compared with ALG hydrogels, maintaining the release for 26 days.^67^ By adjusting the oxidation of ALG, different degradation rates can be achieved, as a way to control the release of encapsulated biomolecules. For example, a microfluidic device was used to fabricate microgels encapsulating lentiviral vectors encoding VEGF. These microgels were composed of different ratios of alginates and were generated by mixing two different types of alginate formulations (different concentration, molecular weight, or oxidation state). They observed a faster release of lentiviral vectors at lower alginate content, due to the faster diffusion promoted by the lower cross-linking, and an increase in the hydrolysis of the hydrogel network, which was attributed to the mix of alginate and oxidized alginates^69^ (Figure 2).

Another approach for the preparation of ALG hydrogels is through its chemical cross-linking, which generally involves the use of low molecular weight cross-linkers or the reaction with other polymers. Wang et al. prepared gelatin cross-linked ALG, hydrogels with the ability to encapsulate proteins such as BMP-2 and sustain the growth of osteoblasts.^26^ ALG hydrogels have also been modified through chemical reactions including Michael addition or Schiff base. Vinyl-sulfone-ALG has been cross-linked through Michael addition to proteins expressing thiol groups. A variant of *C*. *botulinum* C3 transferase could then be immobilized in the hydrogels to have a sustained release of the enzyme and maintain the activity for longer times.^70^

The phase transition of polymers triggered by temperature could be used as a method for the preparation of hydrogels.^71^ Thermoresponsive polymers such as poly(*N*-isopropylacrylamide) (PNIPAAm) can change their conformational structure, leading to a more dehydrated, globular, and hydrophobic state when the temperature increases above the lower critical solution temperature (LCST). PNIPAAM can be blended with or grafted to ALG to fabricate hydrogels with this stimuli-sensitivity, rendering hydrogels with a LCST below body temperature and compatible with the encapsulation of biomolecules such as GF, enzymes, or genes.^72^ For example, hydrogels of ALG-PNIPAAm copolymers were used as gene delivery vehicles for prostate cancer treatment.^73^ A minimum proportion of PNIPAAm in the copolymer is needed to ensure that the LCST of the gel is below the body temperature, thus only 10% of alginate was used in the copolymer. This formulation gelled at body temperature without a requirement for ionic cross-linking, rendering it appropriate for injection. Nanoparticles of pDNA and RALA (an amphipathic cell penetrating peptide) were loaded in those hydrogels, and the release profile was characterized using different ALG types. It was observed that the diffusion of the nanoparticles could be controlled depending on which type of ALG was used. Lower molecular weights and M/G ratios enabled a greater release of the payloads. This higher particle diffusion was promoted by their lower stiffness and simple architecture, which consisted of superimposed layers of polymers. This platform provides evidence that a sustained release of pDNA for up to 1 month with a diminished burst effect is achievable via the combination of the protective effect of RALA and the hydrogel properties.^73^ Other thermoresponsive polymers were used are poloxamers. Segredo-Morales et al. prepared hydrogels of poloxamer-ALG incorporating microparticles with BMP-2 or 17-β-estradiol or plasma rich in GFs. Hydrogels displayed a slow rate of release after a burst release in the first 3 days.^74^

It is also possible to prepare hydrogels by radical polymerization using ALG-methacrylate derivatives. Visible light-curable gels can also be prepared by grafting furfurylamine to ALG and then irradiating the polymer with visible light in the presence of a photosensitizer. Resulting gels exhibit similar mechanical properties to the Ca^2+^ cross-linked ones. The active agent encapsulated in these hydrogels can be released at a rate which is dependent on the molecular weight of the molecule, e.g., about 4 days for IGF-1.^75^

Hybrid systems, including smart nanocomposite hydrogels, have been increasingly proposed for a variety of biomedical applications, including the delivery of active substances.^40^ The addition of nanoparticles or microparticles with the aim of achieving different release profiles has also been explored for GF and genes^76−78^ For example, silk fibroin microspheres encapsulating IGF-1 have shown potential in sustaining its release, with less than 50% of the total volume released after 25 days. This sustained release profile mediated a reduction in the infarct size and an improvement in the heart function on the rat myocardial infarction model within 28 days.^76^

#### 3.1.2 Agarose-Based Hydrogels

Agarose is a natural linear polysaccharide based on d-galactose and 3,6-anhydro-l-galactopyranose units, forming part of agar along with agaropectin.^79^ Several features of agarose support its use as a biomaterial for different cell and/or controlled drug delivery approaches, including its nontoxicity, thermo-reversible nature, interconnected porous microstructure, resemblance to the natural ECM, and long-term stability *in vivo*.^79,80^

Agarose hydrogels were used for the delivery of monoclonal antibodies in a competitive affinity release of a streptavidin–antibody conjugate from agarose-desthiobiotin hydrogels via controlled dissolution of sparingly soluble biotin derivatives.^81^ Release of the conjugate was controlled by adjusting the total biotin derivate concentration without additional antibody or hydrogel modification. Moreover, first-order tunable release of monoclonal antibody bevacizumab, a therapeutic anti-VEGF antibody, was achieved for more than 100 days.

Layer-by-layer (LbL) has been used to assemble complementary polymers for the development of coating and membranes for a variety of biomedical applications.^82^ LbL can also act as a barrier to control the mass transport properties of active release systems.^83^ Functionalization of agarose hydrogels using an LbL assembly technique has been shown to be a potential strategy for the controlled delivery of peptides.^84^ Lynam et al. deployed this technique to load and prevent the diffusion of lysozyme from agarose hydrogels.^85^ Their results showed a relationship between surface area and cumulative dose response that was in the clinically relevant range for the delivery of GFs.

In another approach, Ahearne and Kelly compared the efficiency of agarose, fibrin, and gellan gum hydrogels in the promotion of infrapatellar fat-pad progenitor cells chondrogenesis by encapsulation of transforming growth factor beta 3 (TGF-β3)-releasing gelatin microspheres.^86^ Their results showed a higher deposition of glycosaminoglycans via delivery of TGF-β3-loaded gelatin microspheres from agarose or gellan gum compared with fibrin hydrogels.

The controlled delivery of peptide agents from agarose hydrogels can be based on the use of nanoniosomes, a class of molecular cluster formed by self-association of nonionic surfactants in an aqueous phase normally stabilized with cholesterol.^87^ Moghassemi et al. prepared agarose composite hydrogels for the encapsulation of basic fibroblast growth factor (bFGF)-loaded niosomes. The systems showed sustained release profiles from the loaded compound over the 21-day test-period, leading to a noticeable increase in human umbilical vein endothelial cell (HUVEC) proliferation.^88^Table 1 presents a summary of some of the systems explained.

**Table 1:** Examples of Agarose Hydrogels for the Controlled Delivery of Biomolecules^a^

system	GF/protein	study	results
agarose-desthiobiotin hydrogel^81^	streptavidin-antibody conjugate or anti-VEGF antibody (avastin)	*in vitro*	release can be tuned by altering the total biotin derivative concentration
			first-order release of avastin, for over 100 days
layer-by-layer functionalized agarose hydrogel^85^	lysozyme	*in vitro*	relationship between surface area and cumulative lysozyme dose response
agarose hydrogel with gelatin microspheres^86^	TGF-β3	*in vitro* (IFP-MSCs)	higher GAG deposition when compared with fibrin hydrogels
nanoniosomal hydrogel^88^	bFGF and BSA	*in vitro* (HUVEC)	sustained bFGF release for 21 days
			increased HUVEC proliferation

a Abbreviations: TGF-β3, transforming growth factor beta 3; IFP, infrapatellar pad; MSCs, mesenchymal stem cells; GAG, glycosaminoglycans; bFGF, basic fibroblast growth factor; BSA, bovine serum albumin; HUVEC, human umbilical vein endothelial cells.

#### 3.1.3 Chitosan-Based Hydrogels

The second most abundant polymer in nature, chitin, is the precursor of chitosan (CH), a deacylated linear polysaccharide composed by β-(1–4)-linked d-glucosamine and *N*-acetyl-d-glucosamine units, which are randomly distributed.^89^ Its nontoxicity, biodegradation, and availability make CH an interesting candidate in the development of delivery systems, namely, hydrogels.^90^ CH possesses a variety of functional groups such as polyamine, amino, and hydroxyl which can interact with both cationic and anionic molecules, especially important in the case of proteins. The functionalized polysaccharide could be achieved with the inclusion of several groups along the chain,^91^ leading to multiple possibilities for its use in drug delivery^92^ and tissue engineering,^93^ among others. Hydrogels can be obtained from complexing CH and oppositely charged polysaccharides.^94^ Such highly moldable and versatile systems could be explored in the future in the context of the delivery of therapeutic biomolecules.

Currently, there are some examples of the delivery of bioactive compounds via CH hydrogels. Monoclonal antibodies were delivered from thiolate CH hydrogels for ocular administration.^95^ Covalent-cross-linked carboxymethyl CH hydrogel was also implemented for the codelivery of bevacizumab and 5-fluorouracil to treat postoperative scarring in a rabbit model of experimental glaucoma filtration surgery.^96^ In another study, a dynamic covalent Schiff-base bond was used for the formation of glycol-CH and oxidized ALG for the release of the same monoclonal antibody for the treatment of age-related macular degeneration.^68^ While in terms of tissue engineering, the local delivery of Anti-VEGF antibody from ALG/CH hydrogels prevented bony bar formation in physeal injuries.^97^

Several researchers found the implementation of CH hydrogels useful for the encapsulation of different GFs. In the tissue engineering field, bone morphogenetic protein 6 (BMP-6) and TGF-β3 with human ASCs were included into microspheres: a photopolymerizable N-methacrylate glycol CH *in situ* forming hydrogel was used to incorporate such cargo, enhancing the expression of chondrogenic markers.^98^ CH hydrogels were also involved as a support for hyaluronic acid nanoparticles carrying chondrogenic factors.^99^ In another approach, rhBMP-2 was released from an enzymatically cross-linked injectable glycol CH hydrogel.^100^ To address cartilage tissue defects, chondrogenic factors were included in a CH-beta glycerophosphate-hydroxyethyl cellulose hydrogel.^101^ Transforming growth factor beta 1 (TGF-β1) was chemically linked to methacrylate CH to facilitate the controlled delivery necessary to improve cellular aggregation and ECM deposition^102^ or encapsulated in CH/silk biohydrogel to promote the differentiation of mesenchymal stem cells (MSCs).^103^

CH hydrogels have not only been implemented for bone and cartilage regeneration. Recently, CH hydrogels have been used in wound healing and cardiac regeneration applications. Epidermal growth factor (EGF) coencapsulated with silver ions in CH hydrogels showed enhanced healing of diabetic wounds;^104^ whereas, thermosensitive hydroxybutyl CH encapsulating platelet lysates (PLs) GFs promoted the wound closure.^105^ CH hydrogels were also applied for improving the treatment with MSCs in acute myocardial infarction through the incorporation of the C domain peptide of IGF-1 on hydrogels.^106^

**Figure 3:**
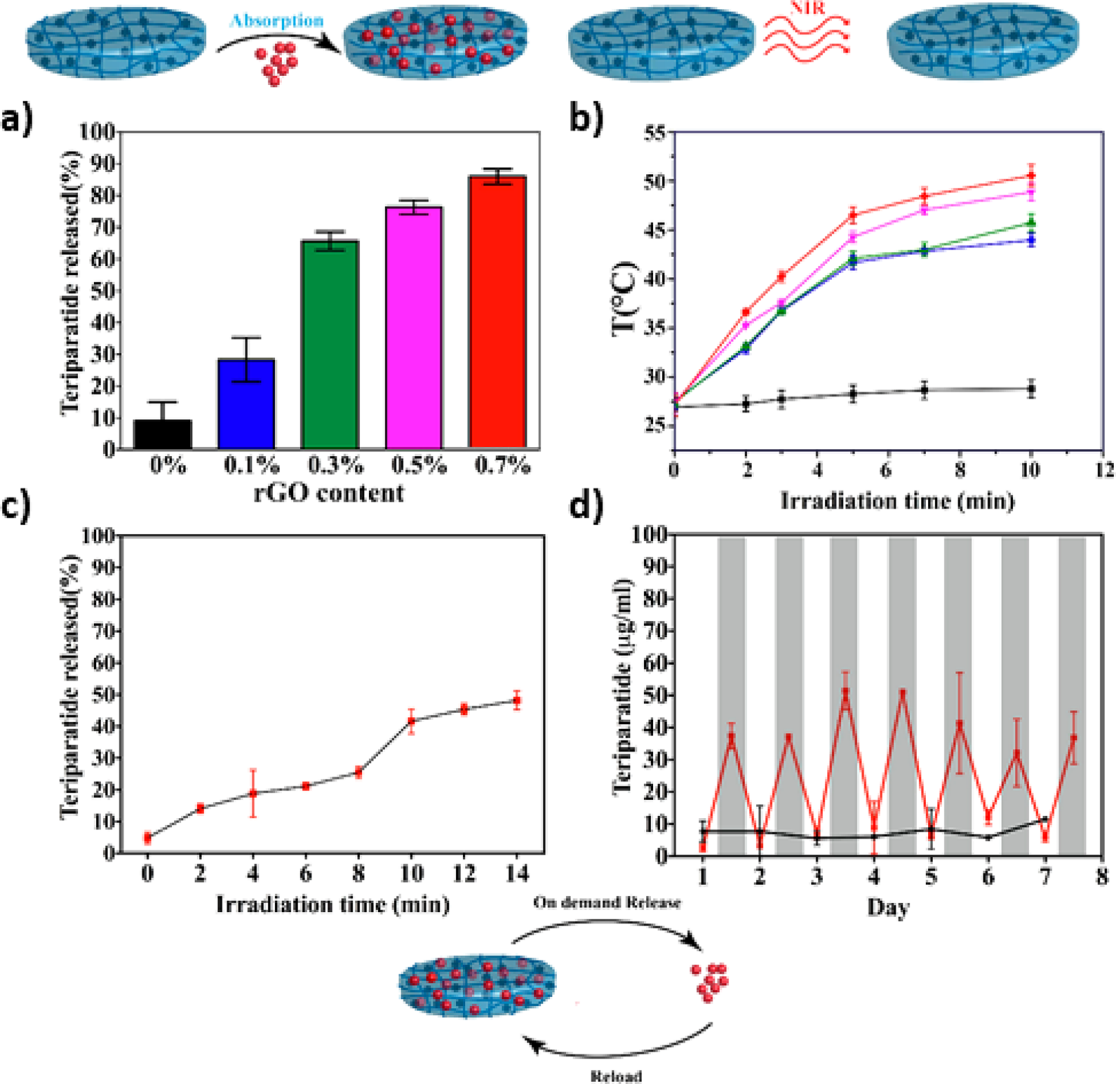
Example of CH-based stimuli sensitive-hydrogel. (a) Drug loading of the hydrogels as a function of rGO concentration in PBS; (b) photothermal heating curves of CS hydrogels (black, 0% content rGO; blue, 0.1% rGO; green, 0.3% rGO; purple, 0.5% rGO; and red, 0.7% rGO) under NIR light irradiation; (c) photothermally drug release from CS/rGO hydrogels; and (d) biomimetic pulsatile secretion of teriparatide in physiological conditions (the black line represents delivery without NIR light, and the red line with NIR light) demonstrating that this system is a novel alternative in the treatment of osteoporotic bone regeneration. Reproduced and edited with permission from ref (116). Copyright 2020, Elsevier.

The administration of hormones in a noninvasive way has been extensively studied by several researchers, particularly for the administration of insulin to diabetic patients, with the aim of attaining an optimal bioavailability of this hormone.^107^ Hence, CH hydrogel formulations were also attempted as insulin delivery systems. Promising *in vitro* results, which showed successful prevention of the typical peaks of blood sugar levels in diabetes, were obtained from a thermosensitive CH hydrogel which delivered insulin as required throughout the day.^108^ A very similar strategy was followed by Naderi-Meshkin et al.^101^ Additionally, transdermal insulin delivery using CH/poly(vinyl alcohol) (PVA) hydrogels was developed.^109^ Furthermore, there are numerous publications which present CH and CH-derivative hydrogels as support for a broad range of insulin delivery systems, including nanoparticles,^110^ vesicles,^111^ micelles,^112^ microspheres,^113^ or stimuli-sensitive delivery systems.^114^ Other hormones, such as exenatide, were also delivered using CH hydrogels;^115^ and recently, Wang et al. produced a combined hydrogel of graphene oxide (GO) and CH able to release teriparatide in a pulsatile and photothermal way, which mimics the release in physiological conditions^116^ (Figure 3). Results showed that hydrogels with higher GO content (0.7%) increased to higher temperatures upon radiation, and this increased their drug loading ability. In addition, the amounts of teriparatide released from these hydrogels were increased with the irradiation time, leading to a pulsatile release.

In gene therapy, CH hydrogels demonstrated adequate characteristics as host of DNA enzymes for topical delivery and were able to prevent the degradation and preserve functional activity in a porcine skin model.^117^ These systems were used for the delivery of lentiviral vectors for prolonging gene expression of therapeutic factors in the nervous system^118^ and for the encapsulation of small interfering RNA (siRNA) in the treatment of periodontitis,^119^ rhinosinusitis,^120^ and osteogenic differentiation,^121^ among others.^122^

#### 3.1.4 Carrageenan-Based Hydrogels

Carrageenan is a linear sulfated polysaccharide, extracted from the cell wall of red seaweeds based on repeating galactose units and 3,6-anhydrogalactose linked alternating α-1,3 and β-1,4 glycosidic linkages.^123^ Due to its gelation properties, immunomodulatory activities, and resemblance to natural glycosaminoglycans, carrageenan hydrogels have been proposed as potential candidates for various tissue engineering applications.^123−126^

Wang et al. prepared a collagen/nanohydroxyapatite/kappa-carrageenan gel for controlled delivery of the nerve growth factor beta (NGF-β) in a mandibular distraction osteogenesis model from rabbit.^127^ When the regeneration from damaged areas was analyzed, a faster bone formation was achieved via implantation of NGF-β-loaded hydrogels, relative to the administration of the free GF in saline solution. Also, carrageenan-based hydrogels containing TGF-β1 have been found to improve the biological performance of encapsulated adipose-derived stem cells in cartilage tissue engineering.^128^

### 3.2 Plant/Seed-Origin Polysaccharides

#### 3.2.1 Starch-Based Hydrogels

Plant-based green hydrogels have been proposed in a range of biomedical engineering applications.^129^ Among the polysaccharides obtained from such sources, starch is an abundant polymeric carbohydrate based on glucose made up of two high molecular weight polysaccharides, amylose and amylopectin, with units joined by α-1,4 and α -1-6 glycosidic bonds.^130^ Amylose is a predominantly linear polysaccharide, and in contrast, amylopectin has a highly branched structure, organized in clusters of short branch chains, giving rise to a relatively compact macromolecular organization. Depending on the nature and the final proportion of each one in the starch, gelation properties could be modulated.^131,132^ Generation of ionizable functional groups resulting from starch copolymerization makes this polysaccharide a good starting biomaterial to prepare stimuli-sensitive hydrogels.^133,134^ This compound also exhibits notable biochemical properties such as nontoxicity and biodegradability.^134,135^

Wöhl-Bruhn et al. designed new hydroxyethyl starch-based polymer derivatives for the production of hydrogels and hydrogel microspheres and for controlled release of fluorescein isothiocyanate-labeled dextran (FD70) and fluorescein isothiocyanate (FITC) labeled human IgG antibody (FITC-IgG).^136^ Functionalization of hydroxyethyl starch with PEG methacrylate or methacrylate polymers led to the formation of hydrogels with optimal water solubility and cross-linking capabilities. Moreover, high encapsulation efficiencies were achieved by tuning the solution ratio between polymer and PEG in the microsphere production process.

In another approach, Faikrua et al. studied the ability of CH/starch/β-glycerol phosphate hydrogel scaffolds to act as a carrier for chondrocytes and delivery system of TGF-β1.^137^ They investigated whether the proposed system could preserve the chondrocyte phenotype and viability after injection. Hydrogels exhibited a sustained release profile of TGF-β1 and resulted in an improved chondrogenesis after 14 days. Similarly, these systems have been shown to be suitable matrixes for the support and retention of chondrocyte function and viability.^137^ More recently, the potential of a starch-based hydrogel for dual release of BMP-2 and bone morphogenetic protein 12 (BMP-12) was tested for a bone-tendon regeneration application.^138^ These systems led to a sustained release pattern of both GFs (∼80% released at 3 weeks), prompting osteogenic and tenogenic cell activities in an *in vitro* primary cell culture model. In terms of the future possibilities, a novel strategy using starch was developed through the LbL technique, which takes advantage of the activity of α-amylase and the degradation of the matrix to control the delivery of DNA.^139^ This work should inform the development of novel systems for gene delivery applications.

#### 3.2.2 Cellulose-Based Hydrogels

Cellulose is a polysaccharide based on a linear chain of d-glucose units bonded by β-1,4 links, present in the protective cell walls of woody portion plants.^140^ Due to the presence of hydroxyl groups in its main chain, cellulose can be functionalized to prepare hydrogels with different structures and properties, acting as platforms for various tissue engineering and regenerative medicine approaches.^140−145^

Considering that cellulose is not soluble in water, it seems reasonable to modify its structure to produce soluble derivatives with highly water affinity, for the preparation of hydrogels. One of them, methylcellulose (MC), has been applied in the preparation of hydrogels for controlled delivery systems of chondroitinase ABC (ChABC) for functional repair in stroke-injured brain^144^ and in a model of spinal cord injury in rats.^142^ Pakulska et al. synthesized a cross-linked methylcellulose (XMC) hydrogel for minimally invasive, localized, and sustained intrathecal delivery of ChABC and stromal cell-derived factor 1α (SDF).^142^ While ChABC was immobilized in XMC hydrogels by protein–peptide affinity interactions, SDF was entrapped by electrostatic affinity interactions. Despite the fact that beneficial tissue and functional outcomes were observed (mostly due to the ChABC treatment), systems were unable to decrease chondroitin sulfate proteoglycan (CSPG) levels after injection. Taking this approach a step further, the same group used these cellulose-based hydrogels to incorporate a PEGylated form of a ChABC mutant variety.^144^ This strategy led to enhancement of the stability of ChABC through site-directed mutagenesis and PEGylation, which resulted in a reduction of protein unfolding and aggregation which prolonged the half-life and enzymatic activity. Furthermore, when implanted into the rat brain cortex, these hydrogels significantly reduced CSPG levels at 28 days postinjury.

Paukkonen et al. investigated the effect of freeze-drying and subsequent rehydration of anionic nanofibrillar cellulose (ANFC) hydrogels on the release profiles of different model compounds, namely, nadolol, metronidazole, and bovine serum albumin.^143^ Results showed that the freeze-drying process with suitable excipients did not significantly impact drug release properties from reconstructed hydrogels. Similarly, a decrease in drug diffusivity was shown by increased ANFC content. This effect was higher with larger protein molecules.

#### 3.2.3 Pectin-Based Hydrogels

Pectin is a complex structural polysaccharide found in the primary cell walls of terrestrial plants, exhibiting a high galacturonic acid content.^146^ Its biodegradable nature, nontoxicity, and ability to form gels in the presence of sugars and acids make pectin a robust candidate for the preparation of hydrogels for delivery systems in diverse wound healing applications.^147−149^

Zhang et al. prepared a bFGF-loaded pectin-based bioinspired hydrogel for stimulating wound healing in a full-thickness excision model from mice.^150^ This hydrogel showed sustained release profiles of bFGF for 1 week, with a slight burst effect within the first hours. When implanted in mice, it resulted in an enhanced wound re-epithelialization, collagen deposition, and contraction without signs of toxicity or inflammation.^150^

Pectin hydrogels have been also applied to deliver completely novel substances. A new molecule, sensitive to reactive oxygen species and based on a neuropeptide, has been delivered using zeolite imidazolate framework-8 (ZIF-8) nanoparticles embedded in a sodium ALG/pectin injectable hydrogel. This novel system was involved in the promotion of wound healing in a full thickness excision mouse model.^151^ By coating nanoparticles with polyethylene glycol thioketal, a responsive release under stimulation with reactive oxygen species was achieved. When implanted *in vivo*, hydrogels prompted an early inflammatory response followed by M2 macrophage polarization.^151^

Apart from applications in wound healing, pectin-based hydrogels have been suggested for other therapies. A gelatin-pectin-biphasic calcium phosphate composite scaffold (Gel-Pec-BCP) was involved in delivering VEGF and/or BMP-2 in a critical size defect model in rats.^152^ Superior bone formation was observed with Gel-Pec-BCP/BMP-2 scaffolds at 4 weeks postimplantation.

In another attempt, a pectin/zein-based hydrogel loaded with *Lactobacillus rhamnosus* GG-derived protein (p40) was synthesized to transactivate the epidermal growth factor receptor (EGFR) in intestinal epithelial cells and to protect the intestinal epithelium against injury and inflammation.^153^ When administered orally in mice, this system delivered bioactive p40 to the small intestine and the colon and led to a significant increase of bodyweight gain (prior to weaning) and functional maturation of the intestine during the postnatal period (day 2 to 21). Likewise, neonatal p40 treatment reduced the susceptibility to intestinal injury and colitis and promoted protective immune responses in adult mice. Table 2 shows a summary of some of the pectin-based hydrogels delivery systems.

**Table 2:** Hydrogels Developed Using Pectin for the Delivery of Therapeutic Biomolecules^a^

system	GF/protein	study	results
pectin/gum arabic/Ca^2+^ hydrogels^150^	bFGF	*in vitro* (scratch assay for wound healing)	enhanced cell proliferation, wound re-epithelialization, and collagen deposition without signs of toxicity or inflammation
		*in vivo* (full-thickness excision wound mice model)	
ZIF-8-PEG-TK@CA nanoparticles encapsulated into an alginate/pectin hydrogel^151^	novel ROS-responsive substance	*in vitro* (HDF and Raw 264.7 macrophages)	responsive release under stimulation by reactive oxygen species
		*in vivo* (full-thickness excision wound healing mouse model)	enhanced proliferation of HDF and up-regulation of inflammation-related genes in macrophages
			early inflammatory response and subsequent M2 macrophage polarization in the wound-healing process
Gel-Pec-BCP^152^	VEGF and BMP-2	*in vitro* (MC3T3-E1 preosteoblasts	increased cell spreading and proliferation
		*in vivo* (critical size defect rats)	higher bone formation with Gel-Pec-BCP/BMP-2 scaffolds
pectin/zein hydrogels^153^	*Lactobacillus Rhamnosus* GG-derived protein (p40)	*in vivo* (oral administration, wild-type mice)	enhanced bodyweight gain and functional maturation of the intestine from mice in early life, except for those with specific deletion of EGFR

a Abbreviations: bFGF, basic fibroblast growth factor; Gel-Pec-BCP, gelatin-pectin-biphasic calcium phosphate composite; VEGF, vascular endothelial growth factor; BMP-2, bone morphogenetic protein 2; EGFR, epidermal growth factor receptor; ZIF-8-PEG-TK@CA, zeolite imidazolate framework-8 (ZIF-8) with polyethylene glycol-thioketal (PEG-TK) nanoparticles encapsulated in injectable hydrogel of sodium alginate and pectin cross-linked using calcium chloride; HDF, human dermal fibroblasts.

### 3.3 Bacteria-Origin Polysaccharides

#### 3.3.1 Dextran-Based Hydrogels

Dextran (DEX) is a nontoxic hydrophilic polysaccharide obtained from *Lactobacillus*, *Leuconostoc*, and *Streptococcus spp*. It is formed by a linear backbone of glucopyranosyl units linked by (1 → 6) bonds that also have some ramifications linked by (1 → 2), (1 → 3), and (1 → 4) bonds.^154^ Its hydroxyl groups can be easily functionalized, enabling the preparation of hydrogels. DEX hydrogels can be prepared by chemical cross-linking (photo-cross-linking,^155^ Michael addition,^22^ Schiff-base reaction,^156^ enzymatic cross-linking^157^) or physical cross-linking.^158^ In general, physically cross-linked hydrogels have lower mechanical strength than chemical ones, but they can be prepared under milder conditions, and therefore damage to the incorporated biomacromolecules is reduced.

One of the most commonly used approaches for the preparation of DEX hydrogels is the photopolymerization of previously functionalized DEX with photopolymerizable groups, such as hydroxyethyl methacrylate (DEX-HEMA) or methacrylate (DEX-MA). Both porosity and stiffness of the DEX-MA hydrogels can be tuned by playing with the MA substitution degree, time of UV light, and photoinitiator concentration. The release of big molecules is usually slow due to the low porosity of these hydrogels, which may limit its use for the delivery of proteins and tissue engineering.^159^ To improve the release of large biomacromolecules from this type of hydrogels, a PEG spacer with carbonate bonds between the polysaccharide and the MA group was added.^160^ This modification changed the degradability profile and subsequently the release of large molecules like myoglobin.^160^

DEX-HEMA is another photopolymerizable derivative that contains a linker sensitive to hydrolysis between dextran and the MA group. Herein, the degradation and protein release can be improved by adding spacers like lactate.^154^ The release of interleukin (IL-2) from DEX-MA, DEX-HEMA, and DEX-lactate-HEMA hydrogels was investigated by Cadée et al. DEX-lactate-HEMA hydrogels provided the fastest IL-1 release while maintaining 50–70% of its biological activity, due to its higher susceptibility to hydrolysis.^161^

DEX photopolymerizable polymer networks have also shown their potential for gene delivery.^162−165^ siRNA was encapsulated by photo-cross-linking of polyethylenimine-MA with the DEX-HEMA backbone.^162^ The hydrogel degradation and siRNA release could be easily controlled by tuning the DEX-HEMA and PEI-MA concentrations. Hydrogels released all the siRNA encapsulated, leading to the knockdown of protein expression in HEK-293 cells.^162^ Hill et al. prepared hydrogels with a gradient of PEI/siRNA of DEX-HEMA encapsulating HEK-293-expressing green fluorescent protein (GFP) cells. A gradient of complexes PEI/siRNA and a spatial regulation of the GFP expression were achieved along the hydrogel, exhibiting the potential of these systems in organ bioengineering.^163^

**Figure 4:**
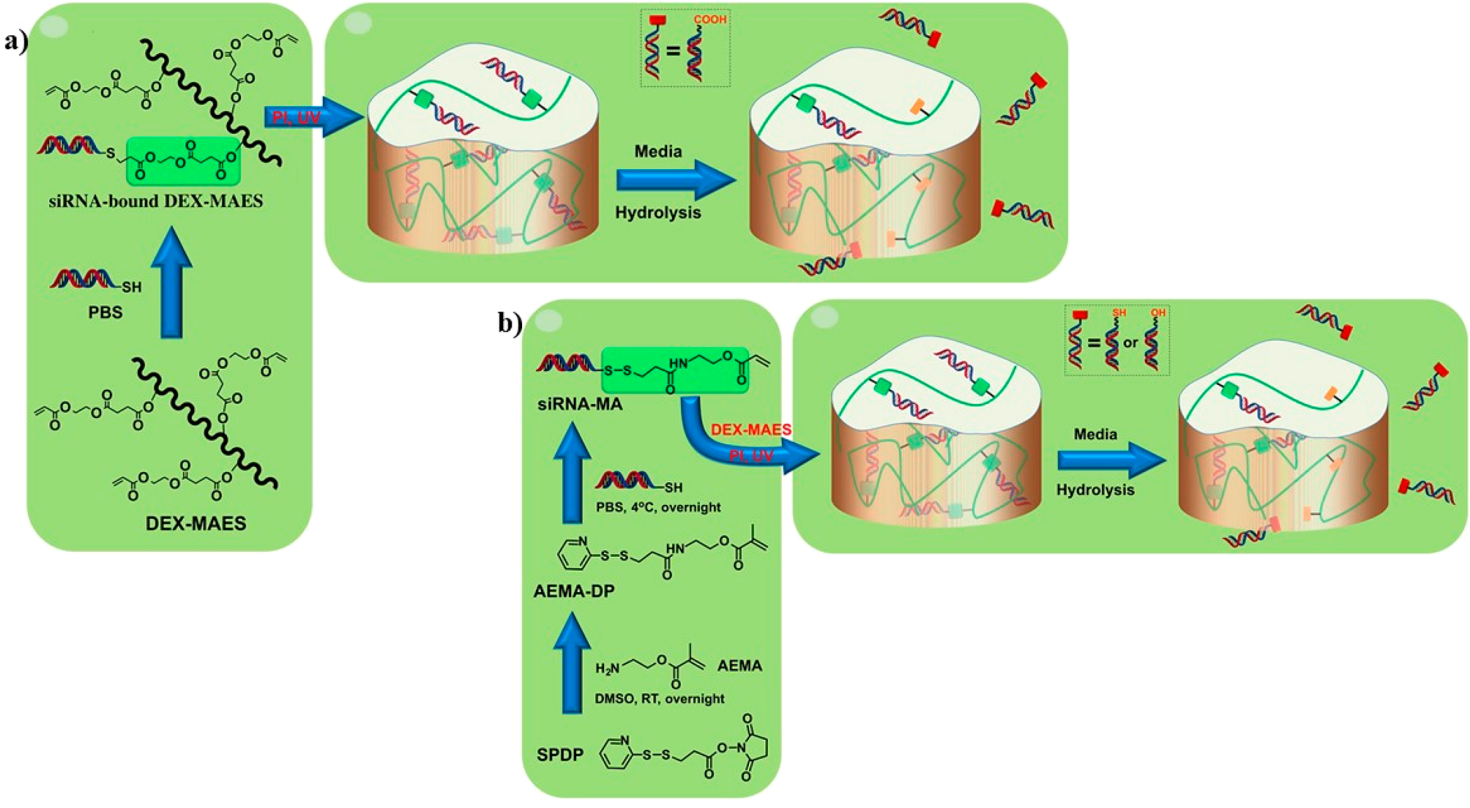
DEX hydrogel implemented for the delivery of siRNA prepared by two different methods: (a) tethering siRNA for the hydrogel via Michael-addition chemistry and (b) via UV conjugation. Reproduced and adapted from ref (168). Open access publication. Copyright 2019, American Association for the Advancement of Science.

DEX hydrogels can also be prepared by cross-linking the chains through a Michael addition reaction between thiol groups and vinyl sulfones or acrylates. This type of reaction happens under physiological conditions, preventing the degradation of sensitive biomolecules. Hiemstra et al. prepared hydrogels of DEX vinyl sulfones and tetrafunctional-mercapto-poly(ethylene glycol), which have an ethyl or propyl spacer between the ester bond/thioether. By adjusting the polymer concentration, the degree of substitution, the molecular weight of the DEX, and the spacer used, both degradation and mechanical properties of hydrogels could be controlled.^166,167^ DEX vinyl sulfone was cross-linked with the thiol groups of 4 thiol-PEG groups for encapsulating chemokine (MIP3) and poly(lactic-*co*-glycolic acid) (PLGA) microparticles loaded with pDNA and siRNA for IL-10. The hydrogels were able to release the MIP3 in a sustained manner and attract dendritic cells. Likewise, hydrogel embedded microparticles codelivering IL-10, siRNA, and plasmid DNA antigens efficiently promoted the migration *in vitro* of primary dendritic cells. Another approach for siRNA delivery was made using the incorporation of mono(2-acryloyloxyethyl) succinate into the DEX backbone (DEX-MAES).^168,169^ DEX-MAES hydrogels loaded with PEI/siRNA complexes were able to sustain the release of siRNA against a BMP antagonist for more than 2 months, which was sufficient to induce the osteogenic differentiation of MSC cultured in the hydrogel.^169^ Nguyen et al. synthesized an siRNA-targeting GFP with the incorporation of thiol and cholesterol groups (siGFP-SH), which can transfect cells without the need of transfection agents.^168^ Some of the acrylate groups of DEX-MAES were reacted with siGFP-SH through Michael-type addition, and the remaining ones were photopolymerized to cross-link DEX-MAES. The thioether ester bonds between the siRNA and DEX are hydrolytically degradable, and the modified siRNA can be released and knockdown the GFP expression of HeLa cells.^168^Figure 4.

Another approach for the preparation of DEX hydrogels is using oxidized dextran (oDex). oDex can self-assemble into hydrogels in the presence of dihydrazide groups, like adipic acid dihydrazide (ADH), due to the interaction between the aldehyde moieties of the oxidized dextran and the dihydrazide groups. These types of hydrogel require at least 3 h for complete gelation, after which time they are biodegraded within 9–23 days.^170^ Ribeiro et al. developed hydrogels of oDex for the encapsulation of CH microparticles containing VEGF and EGF.^171^ When tested as wound dressings, hydrogels enabled faster wound healing without signs of inflammation *in vivo*. Moreover, they showed that only one application of the formulation per week was sufficient to achieve this result.^171^

oDex can also be used for the preparation of hydrogels with gelatin, through the Schiff base reaction between the amine groups of the gelatin and the aldehyde groups of dextran.^156^ Also, the imine linkage is a dynamic covalent bond under physiological conditions, which facilitates a self-healing property of hydrogels.^156^ A sustained release of EGF up to 7 days can be achieved using this system.^172^ Chen et al. developed hydrogels for wound healing with this platform.^173^ Gelatin, ADH, and oDex were formulated in the presence of chlorhexidine and PLGA microspheres encapsulating bFGF. The inclusion of the GF in the microparticles endowed a sequential release of first the antiseptic and then the GF, and when administered into rat wounds, hydrogels accelerated wound closure by preventing infection and promoting healing.^173^ Interestingly, a high-throughput synthesis was used for the creation of hydrazone-cross-linked hydrogels with enable prolonged protein release for 12 days. These hydrogels were prepared by mixing a hydrazide-derivative dextran with an aldehyde-functionalized POEGMA, creating an *in situ*-gelling hydrogel with a minimal burst release and sustained release of a model protein.^174^

The introduction of carboxylate, benzylamide, and sulfate groups into DEX allows the cross-linking of the polymer with sodium trimetaphosphate, under alkaline pH. This new hydrogel has the capacity to bind GFs like TGF-β1 or BMP through ionic interactions with the remaining negative charges of the polysaccharide and sustain their release.^175,176^

Jin et al. modified DEX with epoxy benzophenone-modified carboxymethyl groups cross-linked using horseradish peroxidase (HRP). HRP can catalyze the reaction of anilines or phenols in the presence of hydrogen peroxide under physiological conditions.^177^ DEX-tyramine hydrogels were also modified to incorporate PEG domains in the hydrogel to sustain the release of PEGylated protein. These hydrogels were able to sustain the release of IFN-α2a without a burst effect, and when implanted *in vivo*, they were able to keep the IFN-α2a levels long enough to prevent liver injury in humanized mice with hepatitis C infection.^178^ Teixeira et al. incorporated platelet-rich plasma in DEX-tyramine hydrogels.^179^ This plasma derivative is full of GFs and anti-inflammatory cytokines,^180^ and when introduced into the hydrogels, the chondrogenic differentiation of MSC was promoted without the need for any supplement and was therefore useful in the repair of cartilage defects.^179^ DEX-tyramine can also react with other polysaccharides using the coupling reagent carbodiimide/*N*-hydroxysuccinimide ester. HA was successfully grafted to this dextran derivative; the resulting hydrogels were successfully prepared for the sustained release of VEGF. Endothelial-like cells derived from MSCs cultured in these hydrogels were able to sprout in the scaffold *in vivo* and successfully augment angiogenesis and vascularization.^181^

**Figure 5:**
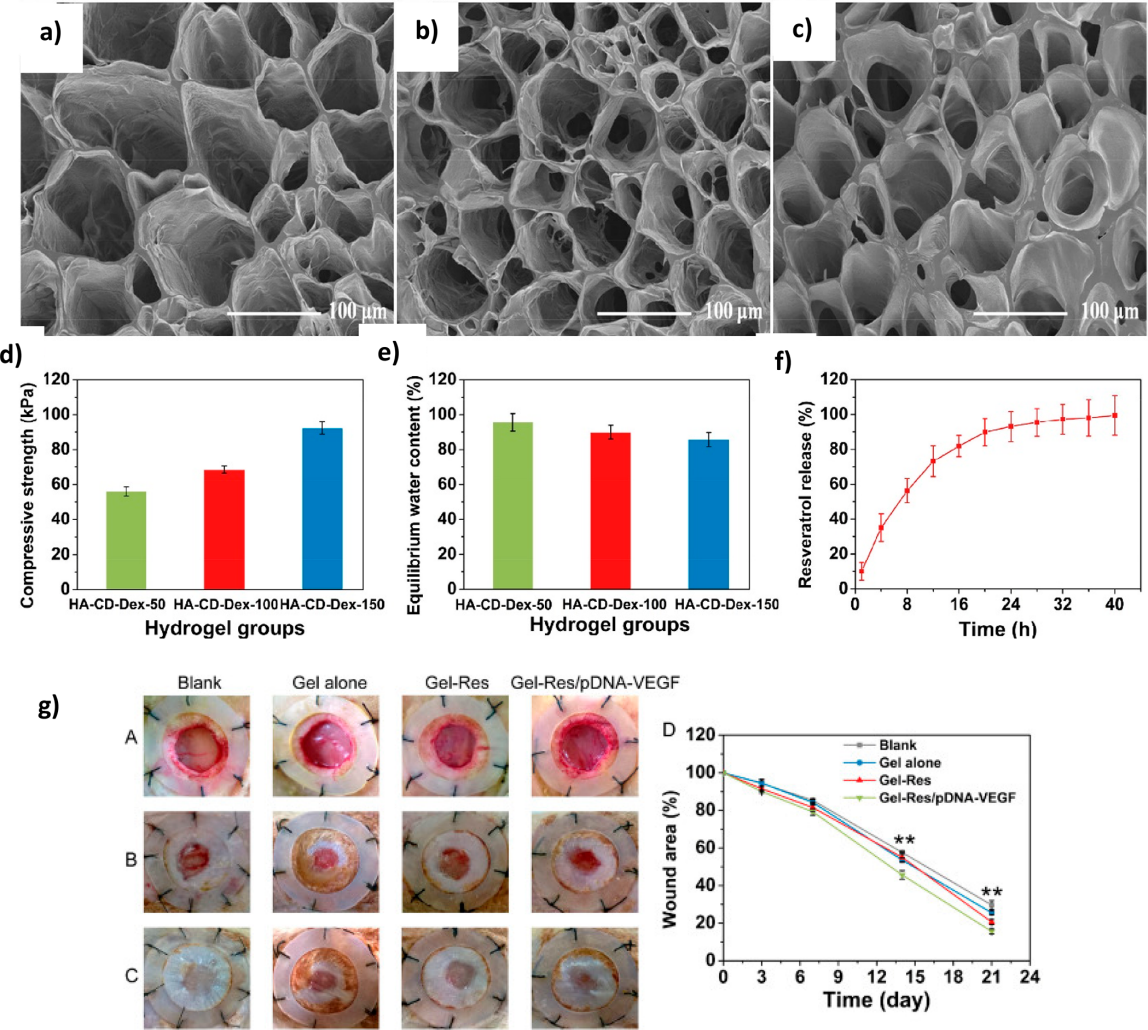
Example of a DEX-based hydrogel system for the delivery of an anti-inflammatory drug encapsulated into β-cyclodextrin and complexed with a plasmid encoding VEGF: Porous structure of (a) HA-CD-DEX-50, (b) HA-CD-DEX-100, and (c) HA-CD-DEX-150 hydrogels; (d) compressive strength of the developed hydrogels; (e) equilibrium water content of the three hydrogel formulations in PBS; (f) resveratrol release from HA-CD-DEX-150 hydrogel. (g) Wound healing efficacy of Gel-Res/pDNA-VEGF scaffold in a burning induced splinted excisional wound model in rats. (A–C) Representative images of wounds at days 7, 14, and 21 after treatment with no treatment, Gel alone, Gel-Res, and Gel-Res/pDNA-VEGF, respectively. (D) Wound closure rates at different time points of all treatment groups Reproduced with permission from ref (189). Copyright 2019, Elsevier.

DEX and its derivatives have been also combined with other polymers such as PVA,^182^ PEGDA,^183^ gelatin,^184^ and ALG^185^ for the fabrication of hydrogels for GF delivery. For example, Sun et al. fabricated hydrogels for immobilizing angiogenic GFs, such as VEGF, which were made of PEGDA and dextran-allyl isocyanate-ethylamine. The porosity of the hydrogel not only enabled the infiltration of cells into the hydrogel but also promoted neovascularization.^186^ Stahl et al. developed a bifunctional peptide with a domain mimicking VEGF (QK) and collagen (CMP). When combined with dextran-allyl isocyanate-ethylamine, these hydrogels augmented VEGF signaling. When topically administered into burn wounds of mice, they enhanced the vasculature repair in the wounds.^187^ Wu et al. fabricated a composite gel for the delivery of two cytokines (IL-2 and interferon-gamma -IFN-γ) and doxorubicin. For the fabrication of this hydrogel, 4-arm PEG-*b*-poly(l-glutamic acid) and hydroxypropyl CH/4-arm PEG-*b*-poly(l-lysine) were ionically cross-linked, and then cholesterol bearing DEX was chemically linked to the CH counterpart.^188^ This bioengineered gel was injectable and was able to protect the cytokines from degradation. Moreover, it simultaneously sustained the release of IL-2, IFN-γ, and doxorubicin into tumors in sufficient amounts to inhibit cancer growth. Wang et al. prepared hydrogels of *N*-hydroxyethylacrylamide-DEX and HA methacrylate cross-linked with PEG-methyl-acrylate-β-cyclodextrin. The anti-inflammatory drug resveratrol was encapsulated within the cyclodextrin, and a complex of PEI with a plasmid encoding for the VEGF was loaded into the hydrogel. This hydrogel was able to inhibit inflammation and promote vascularization in a burn wound model, accelerating its healing.^189^Figure 5.

An RNA-triple-helix consisted of an mRNA duplex tumor suppressor and a small synthetic (ssRNA) to inhibit oncomiR was conjugated to a dendrimer nanocarrier and mixed with dextran aldehyde to form a hydrogel. The resulting system maintained the functionality of the RNA *in vivo* and reduced up to 90% of triple negative breast tumors in mouse models.^190^

Huang et al. prepared thermoresponsive hydrogels of DEX for nerve growth factor (NGF) release by combining it with PNIPAAm and PLA. The formation of pores at 37 °C allowed a faster degradation and GF release for at least 15 days. Moreover, NGF released promoted the neurite outgrowth in PC12 cells, suggesting that the GF kept its biological activity.^191^ DEX-PCL-HEMA/PNIPAAm hydrogels have been also tested as a reservoir for GFs and have resulted in a controlled release. Moreover, when injected into a rat cardiac infarction tissue, an improvement in cardiac function, induction of angiogenesis, and reduced collagen content were observed.^192^ Furthermore, DEX-PCL-HEMA/PNIPAAm hydrogels were able to transfect cells with DNA or RNA.^193,194^ Short-hairpin RNA of angiotensin converting enzyme was conjugated to these hydrogels and injected into a infarct area in a myocardial infarction rat model. A reduction of this enzyme expression was observed *in vivo*, and also an improvement of the heart function and regeneration were observed, illustrating the cardioprotective effects of the gene silencing of this enzyme.^194^

The incorporation of specific cells into the matrix was also considered by some researchers. For example, the development of DEX-based hydrogels for the encapsulation of human embryonic stem cells along with VEGF and a tethered RGD peptide demonstrated both protection and sustained delivery of the GF.^195^

Interestingly, DEX has also being explored for the fabrication of nanomotors. Keller et al. designed asymmetric hydrogel microparticles applying a microfluidic chip. The microparticles consisting of two separated phases: one with droplets of DEX and other one with droplets of PEGDA encapsulating the enzyme catalase. After the decomposition of H_2_O_2_ by the enzyme, the motor of the microparticles propels O_2_ linearly or circularly as required.^196^ These type of developments could inspire the development of novel systems that could combine controlled delivery and sophisticated targeting or transport strategies of the carrier device.

#### 3.3.2 Pullulan-Based Hydrogels

Pullulan is a polysaccharide polymer consisting of maltotriose units connected by α-1,6 glycosidic bonds.^197^ Its biodegradability, nontoxic nature, and water retention capacity and the presence of multiple functional groups for cross-linking, make pullulan an optimal hydrogel-based biomaterial for delivery of cells and biomolecules.^197−199^

Fujioka et al. tested the addition of cholesteryl group- and acryloyl group-bearing pullulan (CHPOA) nanogels for the delivery of fibroblasts growth factor-18 (FGF-18) and BMP-2 in order to promote bone regeneration.^197^ The nanogels increased osteoinductive activity in a mouse calvaria defect model with synergistic effects between both GFs. Recently, biodegradable hydrogels consisting of nanogels and nanogel-coated liposomes were synthesized by cross-linking a CHPOA nanogel and four-arm terminal thiol group pentaerythritol tetra(mercaptoethyl)polyoxyethylene (PEGSH).^199^ The resulting systems led to a complete release of liposome complexes under physiological conditions within 20 days.

A composite scaffold based on pullulan/dextran/interfacial polyelectrolyte complexation (IPC) fibers was investigated for controlled delivery and improved preservation of various biomolecules.^198^ Application of this technique resulted in an entrapment efficiency of 70% of VEGF, with sustained release profiles for 7 days. These results contrast with previous observations, which showed encapsulation efficiencies lower than 20%^200^ with release profiles for 1 day.^201^ Other bacterial-origin polysaccharides can be envisaged in the future for the development of novel delivery systems, as there are huge possibilities of synthesis routes provided by bacteria.^202^

### 3.4 Animal/Human-Origin Polysaccharides

#### 3.4.1 Hyaluronic Acid-Based Hydrogels

Hyaluronic acid (HA) is a linear anionic polysaccharide consisting of repeated disaccharides d-glucuronic acid and *N*-acetylglucosamine bonding with α-(1 → 4) and β-(1 → 3) linkages, respectively. This biopolymer is the main component of the ECM of connective tissue in vertebrates,^203^ with a plethora of physiological functions such as support, viscoelasticity or cell adhesion, and proliferation.^204^ In physiological conditions, it is found as a polyanion (hyaluronan). HA has been used in a wide range of biomedical applications due to its nonimmunogenicity, cytocompatibility, biodegradability, and bioactivity.^204^

In addition, it is well-known that degraded HA fragments can induce angiogenesis and increase the expression of matrix metalloproteinases (MMP)^205,206^ and can activate some cell receptors including CD44 or ICAM-1. In general, HA hydrogels are obtained by the addition of gelling agents or cross-linking agents or the chemical modification of HA.^204^ Cross-linked HA hydrogels can be made by adding cross-linking agents such as divinyl sulfone or glutaraldehyde.^207^ However, cross-linking agents can be cytotoxic or form toxic byproducts, which may limit their use. More biocompatible strategies have been studied for the preparation of HA hydrogels, such as enzymatic cross-linking, click chemistry, or physical cross-linking. Is it even possible to introduce oligonucleotides into the HA chains, enabling cross-linking of the polymer with complementarity sequences.^208^ These new alternatives require the functionalization of HA with other chemical groups that can be introduced in the carboxylate, hydroxyl, or amide groups.

HA hydrogels can be formed via chemical cross-linking of methacrylate groups, which is a commonly used strategy with other biopolymers. These platforms have been used for the release of PLs and GF, and even MSCs have been cultured in such matrixes stimulating their differentiation for applications in periodontal regeneration,^209^ cartilage regeneration,^210^ bone regeneration, or skin regeneration.^211^ For example, HA-GMA photopolymerized hydrogels have been explored for bone regeneration, immobilizing GF such as BMP-2 or VEGF.^212^ The degradation profile of these hydrogels can be tuned by varying the concentration and molecular weight of the HA, leading to diverse GF release profiles and to osteoinductive effects *in vivo*.^212^ The photopolymerizable 2-aminoethyl methacrylate-HA also has potential for bone regeneration when encapsulating the growth and differentiation factor-5 (GDF-5). Hydrogels were able to sustain the release of this agent over 25 days and showed a high proliferation of MC3T3-E1 preosteoblasts. Furthermore, an improvement in osteogenesis was observed *in vivo* when it was administered in a rabbit model of bone defects.^213^ Another application studied for methacrylated-HA hydrogels was cardiac regeneration by encapsulating a tissue inhibitors matrix metalloproteinase (TIMPs).^214^ Interestingly, double cross-linking HA hydrogels were developed by the synthesis of HA particles that were cross-linked with divinyl sulfone and decorated with GMA. Once prepared, particles were included in HA-GMA hydrogels and subsequently polymerized using UV. This double cross-linking allowed a slower release of the payloads.^215^

Furthermore, acrylate-HA was reacted with heparin thiol and a disulfide cross-linker to encapsulate GFβ1 within the hydrogel, as heparin binding domains have affinity for GF. High MW heparins were able to retain larger amounts of GFβ1 and released them slower than low MW heparins. Hydrogels of HA and high MW heparins were also more efficient in differentiating cardiac progenitor cells into endothelial cells.^216^

Another important method for the preparation of HA hydrogels is the thiol–/–ene chemistry, which can be induced by UV light. This process does not involve the use of organic solvents and is also highly specific, which is optimal for cell encapsulation and delivery of sensitive biomolecules. For example, thiol-4-arms-PEGs have been used to cross-link HA-acrylate encapsulating bFGF, to induce the formation of collagen inside the hydrogel by fibroblasts.^217^ With this type of chemistry, it is also possible to add cysteine-containing peptides with different functionalities into the hydrogel, such as MMP-sensitive and cell-adhesive chains. Maleimide-HA hydrogels cross-linked with these peptides were able to sustain the release *in vitro* of BMP-2 and SDF-1α and were sufficient to increase bone formation.^218^ This platform has also been used for the *in vitro* 3D cell transfection of cells.^219−222^ Gojgini and colleagues encapsulated pDNA using this approach and observed that the softer the HA hydrogels, the greater DNA release and transgene expression on cultured MSCs.^220^Table 3 introduces some HA-based hydrogels for the controlled delivery of several GFs.

**Table 3:** Hydrogels Containing HA for the Controlled Delivery of Several GFs^a^

system	GF	study	results
alginate/HA hydrogels^223^	TGF-β3	*in vitro* (MSCs) and *in vivo* (s.c. implantation in nude mice)	superior chondrogenesis and neocartilage formation compared with controls
poly (ε-caprolactone)-collagen/HA hydrogels^224^	VEGF and PDGF	*in vitro* (HUVEC and fibroblasts coculture)	cellular attachment with infiltration and recapitulation of primitive capillary network in the scaffold’s architecture
perlecan/heparan sulfate/HA microgels^225^	BMP-2	*in vivo* (injection in OA model of mice)	treated knees had higher mRNA levels and lesser OA-like damage compared to control knees
bisphosphonate-linked HA hydrogel^226^	BMP-2	*in vitro* (rat osteoblasts or rat MSCs)	bioactive BMP-2 release by enzymatic degradation of the hydrogels
HA hydrogels with peptide-binding dendrimers^227^	BMP-2 or TGF-β1	*in vitro*	significantly lower amounts of growth factors released in the presence of the affinity binding peptide macromolecule
photo-cross-linkable HA/platelet rich plasma complexed hydrogel glue^228^	PDGF, TGF-β1, and FGF	*in vitro* (fibroblasts) and *in vivo* (rabbit cartilage defect model)	increased cell proliferation and migration
			integrative hyaline-like cartilage formation *in vivo*
HA hydrogels reinforced with cellulose nanocrystals and enriched with PLs^229^	PDGF and VEGF	*in vitro* (hDPCs) and *in vivo* (CAM)	stimulated chemotactic and pro-angiogenic activity by promoting hDPCs recruitment and cell sprouting
HA/collagen hydrogels containing high-sulfated HA microgels^230^	TGF-β1	*in vitro*	increased TGF-β1 retention and retarded release
HA/heparin hydrogels^231^	BMP-6	*in vitro* (myeloma cells or MSCs)	induced osteogenic differentiation and decreased viability of myeloma cell lines
HA hydrogel/nanohydroxyapatite particles^232^	BMP-2	*in vivo* (s.c. implantation in mouse)	addition of hydroxyapatite nanoparticles modified the release pattern of BMP-2, resulting in enhanced bone formation
gelatin/HA hydrogel^233^	FGF-10 and FGF-7	*in vitro* (Calu-3 or MSCs)	epithelial phenotype of MSCs after 2 weeks with reduction of vimentin and increase in pan cytokeratin expression

a Abbreviations: BMP-2, bone morphogenetic protein 2; TGF-β3, transforming growth factor beta 3; MSCs, mesenchymal stem cells; s.c., subcutaneous; VEGF, vascular endothelial growth factor; PDGF, platelet-derived growth factor; mRNA, mRNA; HUVEC, human umbilical vein endothelial cells; OA, osteoarthritis; TGF-β1, transforming growth factor beta 1; FGF, fibroblast growth factor; PLs, platelet lysates; hDPCs, human dental pulp cells; CAM, chick chorioallantoic membrane; BMP-6, bone morphogenetic protein 6; FGF-10, fibroblast growth factor 10; FGF-7, fibroblast growth factor 7; FITC, fluorescein isothiocyanate; NT-3, neurotrophin-3; BSA, bovine serum albumin; PLL, polylysine; HEK, human embryonic kidney.

Thiol groups can also be introduced in HA chains. There are commercially available HA-thiol hydrogel kits that can be used for the encapsulation of GF and cytokines for bone regeneration,^234,231^ lung regeneration,^235^ or neurogenesis.^236^ The application of the commercial kit with heparin allows higher IL-10 encapsulation, and the IL-10 released *in vivo* in a mouse model of lung injury demonstrated a reduction in the deposition of collagen in the lung parenchyma. Therefore, this strategy warrants further explorations as a potential treatment for fibrotic lung disorders.^235^ Moreover, this platform can be used for the encapsulation of GF and cells to study cell differentiation. Human urine-stem cells were encapsulated in the heparin/HA/PEGDA hydrogels within a cocktail of GFs that enhance neurogenesis (NGF, FGF), angiogenesis (VEGF), and myogenesis (IGF1, HGF, PDGF-BB). The injection of these hydrogels in athymic mice resulted in an improvement of vascularization, innervation, and myogenic differentiation.^236^ Wang and co-workers developed a conductive hydrogel consisting on HA-thiol cross-linked with tetraaniline-PEG diacrylate for the treatment of myocardial infarction. They loaded the hydrogel with pDNA encoding for endothelial nitric oxide synthase nanocomplexes and adipose derived stem cells. *In vivo* experiments demonstrated increased expression of the encoded gene and proangiogenic GF, and an improvement of heart function was observed.^237^ Additionally, aminoethyl methacrylated-HA and thiolated HA were used for the formation of hydrogels for human growth hormone (hGH) release in a rat model.^238^

Another approach for the preparation of HA hydrogels under mild conditions is through hydrazone cross-linking, which consists of mixing carbohydrazide-HA and an aldehyde-HA derivative or other polymers.^239,240^ This method leads to highly biocompatible and stable hydrogels, suitable for cell transfection. For example, aldehyde-HA/hydrazide-HA hydrogels can form stable complexes with DNA and efficiently transfect cells for expressing CD44.^241^ These hydrazine cross-linked HA hydrogels were used to protect human recombinant BMP-2 from degradation. These hydrogels can modulate the release of BMP-2 by tuning the protonation state of the carboxylic group with the change of pH, demonstrating a superior bone formation when acidic hydrogels were ectopically administered in rats.^242^

In another study, cellulose nanocrystals were included in the matrix, to protect the hydrogels from degradation. Silva and colleagues showed that when these hydrogels were used to encapsulate PLs, a sustained release of GF such as PDGF and VEGF could be observed. Interestingly, these hydrogels were able to recruit and promote the sprouting of human dental pulp and endothelial cells.^229^

**Figure 6:**
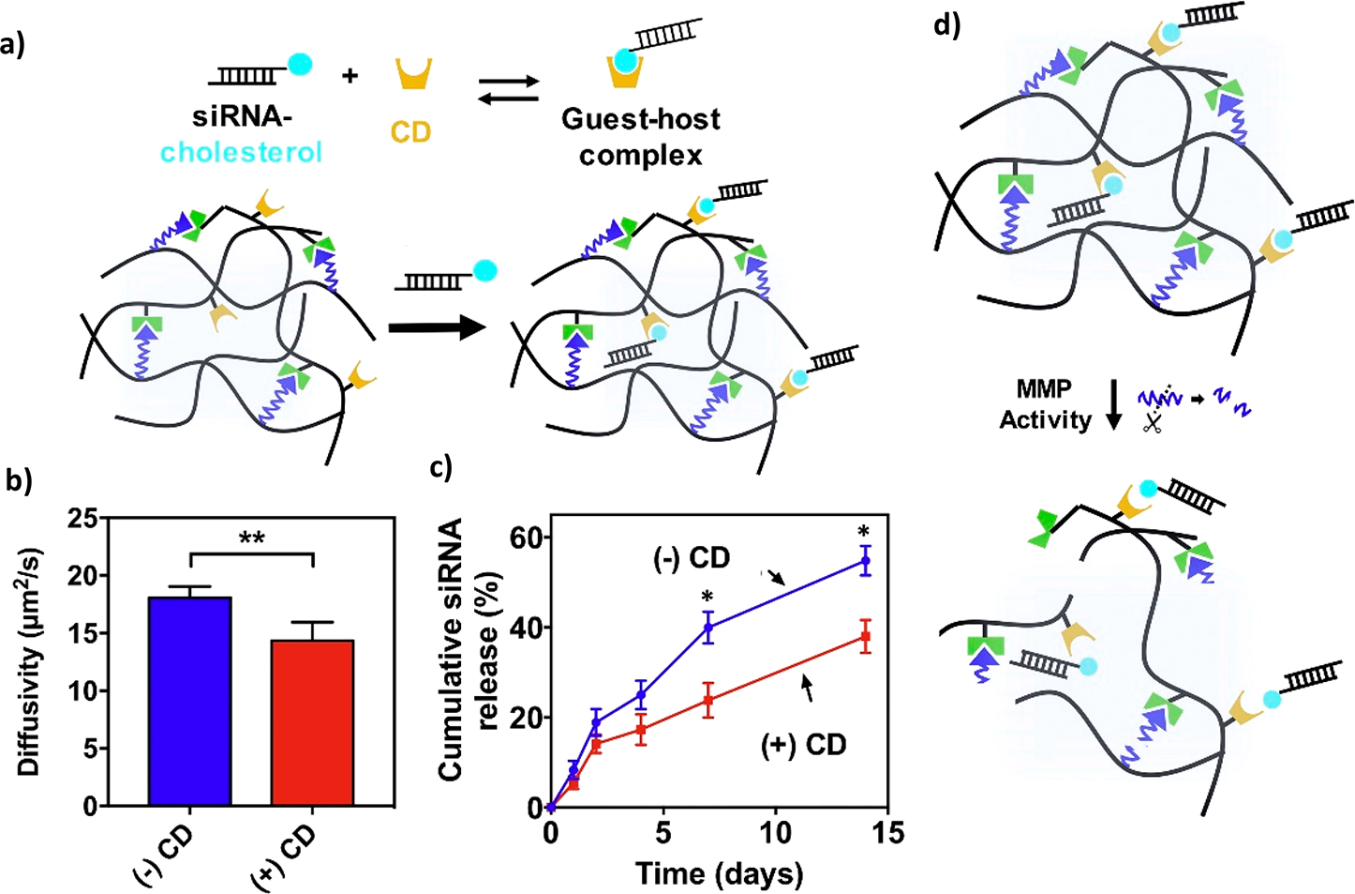
Example of HA hydrogels that encapsulate siRNA: (a) schematic of the siRNA-cholesterol interaction with cholesterol/CD interactions, (b) fluorescence recovery after photobleaching diffusivity with encapsulated fluorescein (FAM)-modified, with (+) and without (−) CD, (c) cumulative FAM-modified, cholesterol-modified siRNA release from hydrogels with or without CD, and (d) schematic of erosion and si-RNA response to delivery of active MMP. Reproduced and adapted with permission from ref (243). Copyright 2018, Elsevier.

HA hydrogels were applied to encapsulate siRNA against MMP-2 to test its potential in the treatment of myocardial infarction. The researchers introduced MMP sensitive peptides functionalized with hydrazide and β-cyclodextrin (CD) into the HA chains. CD enabled the siRNA complexation before its modification with cholesterol groups. siRNA release was mainly promoted by the hydrogel erosion by proteases, all of which were released in less than 5 days compared to the nonrelease observed without the enzyme. The released siRNA reduced the expression of MMP-2 in rat primary cardiac fibroblasts.^243^ (Figure 6).

Enzymatic cross-linking is another strategy used for the preparation of HA hydrogels under mild conditions. For example, Levison and colleagues introduced peptides that are substrate for transglutaminases and heparin into HA chains. Both polymers could be cross-linked together by adding thrombin and transglutaminase, enabling the encapsulation of TGF-β1. This hydrogel could sustain the release of TGF- β1 for long time. Moreover, when chondroprogenitor cells were encapsulated in the same hydrogel, this slow release allowed an efficient matrix deposition, appropriate for cartilage repair.^244^ HRP is the most used enzyme for the enzymatic cross-linking of HA. Using these strategy, tyramine functionalized HA (HA-Tyr, Corgel) is cross-linked in the presence of HRP and hydrogen peroxide. HA-Tyr hydrogels have been extensively investigated for the release of GF and antibodies^245−247^ and can be biodegraded by hyaluronidases. For example, PLs were encapsulated in a HA-Tyr hydrogel to study chondrogenic differentiation.^248^ Moreover, Egbu and colleagues compared the release of the antibody infliximab using the PK-Eye model from hydrogels made of HA-Tyr or poly(ethylene glycol) diacrylate (PEGDA), poly *N*-isopropylacrylamide (pNIPAAM), and HA cross-linked by APS/TEMED. HA-Tyr hydrogels showed a faster release of the antibody than the polymerized cross-linking ones.^249^ Other HA derivatives that can be used for the HRP cross-linking are HA conjugated to (−)-epigallocatechin-3-gallate (EGCG). This enzymatic cross-linking can be carried out without the presence of exogenous H_2_O_2_, as the autoxidation of EGCG produces H_2_O_2_. Moreover, when this type of cross-linking is used, the enzymatic degradation of the hydrogels can be reduced. Interestingly, HA-gallols can also be cross-linked in the presence of gallol-rich compounds such as oligo-epigallocatechin gallate rendering hydrogels with low enzymatic biodegradation and high protein encapsulation through noncovalent interactions with the gallols.^250^ This behavior is explained by the high affinity of the hyaluronase with the gallols, which entrap the enzyme.^251^

**Figure 7:**
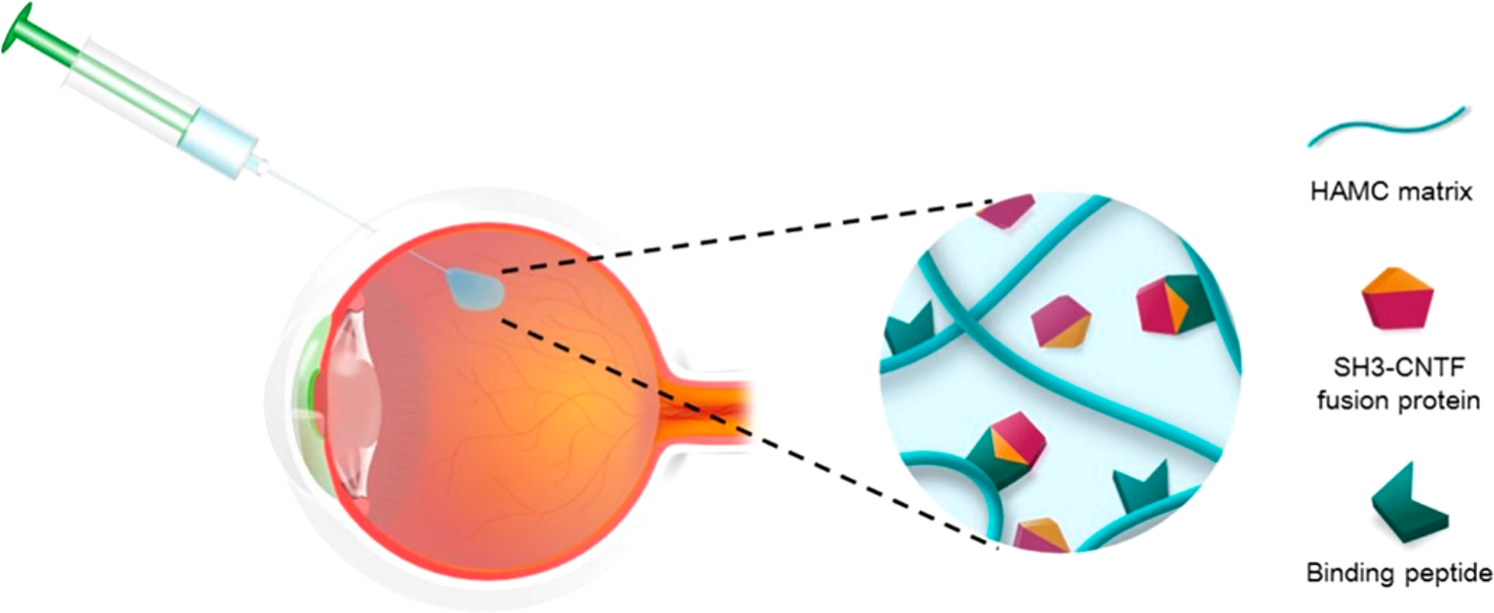
Schematic representation of intravitreal administration of ciliary neurotrophic factor (CNTF) applied using an affinity-based release system based on a HA hydrogel. The system was able to release CNTF over 7 days with the same stability and bioactivity as commercial ones. Reproduced with permission from ref (259). Copyright 2019, Elsevier.

One of the most commonly used strategies for the formation of physically cross-linked HA hydrogels is the combination of HA with gelling agents, such as poloxamer,^252^ ALG,^253^ collagen,^254^ or more frequently, methylcellulose (MC).^255,256^ For example, an HA-MC hydrogel was able to sustain the release of IGF-1. The authors modulated the delivery by adding Src homology 3 (SH3)-binding peptides to the polymer chains.^257^ A similar strategy was followed by Vulic and Shoichet, who encapsulated SH3-rhFGF2 that slowed down the GF diffusion. Different release profiles were obtained by changing the affinity of the binding peptide SH3. Interestingly, the release was linear, without a burst release.^258^ This platform has shown its efficacy in intravitreal GF delivery and was completely nontoxic.^259^ (Figure 7).

Another alternative is the inclusion of the GF in nanoparticles/microparticles to slow down the release of therapeutic biomolecules.^260^ This approach was used by Obermeyer et al., who encapsulated brain-derived neurotrophic factor (BDNF) in hydrogels made of HA-MC including PLGA nanoparticles. BDNF delivery improved the plasticity and reduced the stroke lesion size in a rodent model.^261^

Alternatively, self-assembled physical hydrogels can be formed by inclusion of hydrophobic polymers into the HA chain. For example, PNIPAAm was grafted into HA, resulting in thermoresponsive hydrogels. Pereira et al. encapsulated SDF to be used as implants in intervertebral disc to recruit MSCs.^262^ Hydrogels were able to release around 50% of the GF in 7 days, which was enough to attract MSCs to the disc *ex vivo*. These platforms are also potential candidates for the encapsulation of GFs for various functions, including the potentiation of MSC differentiation to intervertebral disk-like cells,^263^ bone regeneration,^264^ or for the delivery of antibodies into the eye.^265^ Following this idea, Steele et al. fabricated a hydrophobic HA derivative that can be cross-linked by poly(ethylene glycol)-*block*-poly(lactic acid) (PEG-PLA) nanoparticles through the self-assembly of both components. SDF was encapsulated inside NPs, and a dimeric fragment of hepatocyte growth factor (HGF) was added into the hydrogel, to obtain a dual-stage GF release. In this regard, the extended time of GF release was sufficient to ensure the reduction of a scar in both sheep and rat models of myocardial ischemia.^266^

Another approach to produce cross-linking in HA is based on supramolecular guest–host interactions. The most representative guest–host pair used for cross-linking HA is adamantane and β-cyclodextrin.^267^ By grafting both components into the HA chain, hydrogels can be formed by the complexation of both molecules in aqueous solutions. The stiffness of the hydrogel can be easily modulated by adjusting the proportions of both components. Interestingly, these hydrogels have also shear-thinning properties and can be easily injected. Rodell and co-workers showed that these hydrogels can sustain the release of IL-10 and anti-TGFβ for 3 weeks *in vitro*, through the mechanism of erosion of the hydrogel.^268^

#### 3.4.2 Chondroitin Sulfate-Based Hydrogels

Chondroitin sulfate (CS) is a linear polysaccharide based on (1–3)-β-*N*-acetyl-d-galactosamine and (1–4)-β-glucuronic acid, which exhibits sulfate, hydroxyl, and carboxylic acid functionalities.^269^ Due to its affinity for GFs and stem cell regulatory functions, CS has been widely used in different regenerative medicine approaches for the development of adapted delivery systems for various GFs^269−274^ and proteins.^275,276^

CS has been involved as a starting material in different neuro-regenerative approaches^277^ due to its role in guidance of neuronal migration during neural development.^38,270^ Conovaloff et al. synthesized a bioactive synthetic peptide/CS hybrid hydrogel to augment primary cortical neurite outgrowth via enhanced binding of the NGF.^270^ Addition of the peptide to CS hydrogels resulted in a sustained NGF diffusivity and enhanced cell growth. A similar trend was observed by incorporating a CS-binding peptide to CS hydrogels.^271^

Due to its abundance in the cartilage ECM, CS has been extensively used as a scaffolding hydrogel biomaterial for cartilage repair.^38,269,275^ Du et al. synthesized porous hydrogel scaffolds by coprecipitation of CS and collagen for dual GFs delivery.^273^ The presence of the collagen binding domain (CBD) on bFGF resulted in sustained release profile of this GF over time, which promoted hMSCs differentiation into chondrocytes. More recently, Chen et al. incorporated low molecular weight heparin into carboxymethyl CH-oxidized CS hydrogels loaded with trans-TGF-β3 and peripheral blood MSCs.^269^ The presence of heparin improved the mechanical properties of the hydrogels, enhancing their stability and mediating sustained release of TGF-β3 for 3 weeks. Furthermore, these new scaffolds provided a favorable microenvironment for MSCs.^269^

The influence of the degree of sulfation of CS-based hydrogels on the release profiles and bioactivity of a positively charged model protein (histone) have been evaluated.^275^ A reduction in histone release was noted from desulfated chondroitin-based hydrogels when compared to native counterparts. In addition, higher type-II collagen and aggrecan expressions were observed when MSCs were encapsulated in desulfated chondroitin hydrogels.

Encapsulation of biomolecules in flexible networks can provide unique features, such as preservation of biomolecule activity and controlled delivery. Schuurmans et al. synthesized methacrylated CS (CSMA) microhydrogel spheres to study the absorption of lysozymes.^276^ When embedded into a thermosensitive hydrogel scaffold, lysozyme-loaded CSMA microgels were completely released in 58 days.

#### 3.4.3 Heparin-Based Hydrogels

Heparin is a linear polysaccharide consisting of 1–4 linked disaccharide repeating units of uronic acid and glucosamine residues.^278^ The prevalence of sulfate and carboxylate groups endows heparin with a high negative charge and promotes its interaction with various proteins such as GFs, chemokines, and proteases.^278,279^ For this reason, heparin-based hydrogels have been widely exploited for the controlled delivery of GFs in diverse tissue engineering approaches.^280,281,290−293,282−289^

Various heparin/gelatin hybrid hydrogels have been prepared for delivering VEGF to promote angiogenic responses in many tissue engineering approaches.^282,289,290^ The interaction of heparin with GFs such as VEGF can both maintain the activity of the GF and act as a controlled delivery system. Resulting hydrogels provided sustained release profiles for at least 3 weeks,^282,289,290^ exhibiting bioactivity both *in vitro*(282) and *in vivo*.^289^

Thermosensitive hydrogels, based on heparin combined with poloxamer, have been synthesized to obtain *in situ* gelling systems that can be implanted using minimally invasive procedures.^283,288,294^ The delivery of NGF^283^ or keratinocyte growth factor (KGF)^288^ via these hydrogels has shown efficacy in both nerve regeneration^283^ and endometrial wound healing.^288^ More recently, such hydrogels were proposed for codelivering bFGF and NGF in a sciatic nerve crush injury in diabetic rats.^294^ Upon injection, the systems led to a localized release of both GFs over 30 days with improved recovery of motor functions.

Modular hydrogels based on cross-linking multiarmed and end-functionalized PEG (star-shaped PEG) with heparin have been prepared in order to combine structural flexibility of star-shaped PEG with the affinity from heparin to various GFs.^281,291^ When these hydrogels were locally injected in an acute kidney injury model from mice, bFGF-loaded hydrogels demonstrated a significant increase in cell proliferation, although small effects were also noted in the noninjected kidney due to a side systemic effect.^281^ In parallel, both foreign body reactions and hydrogel tissue integration could be effectively controlled by defined adjustments of the PEG/heparin hydrogel systems.^291^

More recently, a codelivery strategy was tested using self-assembling peptide/heparin hydrogels containing an inflammatory agent (TNF-α) and a proliferative factor (HGF), with the aim of treating ischemia-reperfusion-induced organ injury.^293^ When injected in mice, hydrogels could sequentially deliver the two biomolecules achieving anti-inflammatory and pro-proliferative effects with a single administration.

In an innovative approach, the production of 3D printed heparin-based hydrogels with customizable geometry and controlled delivery properties was described.^292^ By increasing the shell layers’ thickness, a decrease could be obtained in the release rate of VEGF and platelet derived growth factor (PDGF). By switching the spatial order, the delivery sequence of the GFs could be modulated and predicted using a mathematical model.

## Conclusions

This review reflects on the enormous progress that has been made in the development of polysaccharide-based hydrogels for therapeutic delivery, portraying a broad picture of recent advances, in particular, over the past decade. The potential of polysaccharide hydrogels is augmented by their nontoxicity, biodegradability, capacity to respond to physiological stimuli and reabsorption, as well as the fact that they do not need to be surgically removed (avoiding secondary complications), which render these systems extraordinary candidates for the development of delivery systems for biotherapeutics in combination with several cutting-edge technologies. These hydrogels demonstrated their suitability for the delivery of biotherapeutics in a controlled manner; and consequently, an impressive amount of delivery systems for biotherapeutics have been developed in the past decade using polysaccharide-based hydrogels.

From the materials side, we expect that more precise chemical modifications of polysaccharides, and their combination with other polymers or nanobiomaterials, could improve the general structural behavior of the systems (e.g., mechanical properties or degradation profile) and tune the interaction with the bioactive agents at the molecular and nanoscale level. On a larger scale, the progress of 3D printing and bioprinting will permit the production of highly reproducible and complex structures based on polysaccharide hydrogels, including heterogeneous systems able to deliver biotherapeutics with both temporal and spatial controlled release.

In the near future, it is expected that novel systems, like the ones discussed in this review, could give rise to new marketable products to treat a broad range of pathologies. To this end, efforts aiming to progress systems based on natural polymers, such as polysaccharides, into clinical use should be intensified, with the aim of overcoming regulatory issues, which are currently considered as a major obstacle.

## Abbreviations Used

VEGF: vascular endothelial growth factor
IGF-1: insulin-like growth factor-1
BMP-2: bone morphogenetic protein 2
FGF: fibroblast growth factor
pDNA: plasmid DNA
GF: Growth factor
ECM: extracellular matrix
TGF-β3: Transforming growth factor beta 3
BMP-6: bone morphogenetic protein 6
EGF: Epidermal growth factor
MSCs: Mesenchymal stem cells
s.c.: subcutaneous
siRNA: small interfering RNA
NGF-β: nerve growth factor beta
FD70: fluorescein isothiocyanate-labeled dextran with a molecular weight of 70 kDa
FITC-IgG: fluorescein isothiocyanate-labeled IgG-human antibody
PEG: polyethylene-glycol
ChABC: chondroitinase ABC
SDF: stromal cell-derived factor 1α
CSPG: chondroitin sulfate proteoglycan
ANFC: anionic nanofibrillar cellulose
HEK: human embryonic kidney
IL-10: Interleukine-10
HRP: horseradish peroxidase
PDGF: platelet-derived growth factor
mRNA: mRNA
HUVEC: Human Umbilical Vein Endothelial Cells
TGF-β1: Transforming growth factor beta 1
hDPCs: human dental pulp cells
FGF-10: fibroblast growth factor 10
FGF-7: fibroblast growth factor 7
FITC: fluorescein isothiocyanate
NT-3: neurotrophin-3
BSA: bovine serum albumin

## References

[ref1] Peppas N. A.; Hoffman A. S. Hydrogels. In Biomaterials Science An Introduction to Materials in Medicine; Wagner W. R., Ed.; Elsevier Inc.: Pittsburgh, PA, 2020; pp 153–166, 10.1016/B978-0-12-816137-1.00014-3.

[ref2] Li J.; Mooney D. J. Designing Hydrogels for Controlled Drug Delivery. Nat. Rev. Mater. 2016, 1 (12), 1–18. 10.1038/natrevmats.2016.71. PMC5898614 29657852

[ref3] Vermonden T.; Censi R.; Hennink W. E. Hydrogels for Protein Delivery. Chem. Rev. 2012, 112 (5), 2853–2888. 10.1021/cr200157d. 22360637

[ref4] Censi R.; Di Martino P.; Vermonden T.; Hennink W. E. Hydrogels for Protein Delivery in Tissue Engineering. J. Controlled Release 2012, 161 (2), 680–692. 10.1016/j.jconrel.2012.03.002. 22421425

[ref5] Jin M.; Shi J.; Zhu W.; Yao H.; Wang D. Polysaccharide-Based Biomaterials in Tissue Engineering : A Review. Tissue Eng., Part B 2021, 1–76. 10.1089/ten.teb.2020.0208. 33267648

[ref6] Carballo-Pedrares N.; Fuentes-Boquete I.; Díaz-Prado S.; Rey-Rico A. Hydrogel-Based Localized Nonviral Gene Delivery in Regenerative Medicine Approaches—an Overview. Pharmaceutics 2020, 12 (8), 752 10.3390/pharmaceutics12080752. PMC7464633 32785171

[ref7] Prabaharan M.; Mano J. F. Stimuli-Responsive Hydrogels Based on Polysaccharides Incorporated with Thermo-Responsive Polymers as Novel Biomaterials. Macromol. Biosci. 2006, 6 (12), 991–1008. 10.1002/mabi.200600164. 17128423

[ref8] Mano J. F.; Silva G. A.; Azevedo H. S.; Malafaya P. B.; Sousa R. A.; Silva S. S.; Boesel L. F.; Oliveira J. M.; Santos T. C.; Marques A. P.; Neves N. M.; Reis R. L. Natural Origin Biodegradable Systems in Tissue Engineering and Regenerative Medicine: Present Status and Some Moving Trends. J. R. Soc., Interface 2007, 4 (17), 999–1030. 10.1098/rsif.2007.0220. 17412675 PMC2396201

[ref9] Cardoso M. J.; Costa R. R.; Mano J. F. Marine Origin Polysaccharides in Drug Delivery Systems. Mar. Drugs 2016, 14 (2), 34 10.3390/md14020034. PMC4771987 26861358

[ref10] Coviello T.; Matricardi P.; Marianecci C.; Alhaique F. Polysaccharide Hydrogels for Modified Release Formulations. J. Controlled Release 2007, 119 (1), 5–24. 10.1016/j.jconrel.2007.01.004. 17382422

[ref11] Miao T.; Wang J.; Zeng Y.; Liu G.; Chen X. Polysaccharide-Based Controlled Release Systems for Therapeutics Delivery and Tissue Engineering: From Bench to Bedside. Adv. Sci. 2018, 5 (4), 1700513 10.1002/advs.201700513. PMC5908359 29721408

[ref12] Chen J.; Jo S.; Park K. Polysaccharide Hydrogels for Protein Drug Delivery. Carbohydr. Polym. 1995, 28 (1), 69–76. 10.1016/0144-8617(95)00080-1.

[ref13] Cohen M. D.; Keystone E. Rituximab for Rheumatoid Arthritis. Rheumalology Ther. 2015, 2, 99–111. 10.1007/s40744-015-0016-9. PMC4883263 27747531

[ref14] Guziewicz N.; Best A.; Perez-Ramirez B.; Kaplan D. L. Lyophilized Silk Fibroin Hydrogels for the Sustained Local Delivery of Therapeutic Monoclonal Antibodies. Biomaterials 2011, 32 (10), 2642–2650. 10.1016/j.biomaterials.2010.12.023. 21216004 PMC3032024

[ref15] Pinto D.; Park Y. J.; Beltramello M.; Walls A. C.; Tortorici M. A.; Bianchi S.; Jaconi S.; Culap K.; Zatta F.; De Marco A.; Peter A.; Guarino B.; Spreafico R.; Cameroni E.; Case J. B.; Chen R. E.; Havenar-Daughton C.; Snell G.; Telenti A.; Virgin H. W.; Lanzavecchia A.; Diamond M. S.; Fink K.; Veesler D.; Corti D. Cross-Neutralization of SARS-CoV-2 by a Human Monoclonal SARS-CoV Antibody. Nature 2020, 583 (7815), 290–295. 10.1038/s41586-020-2349-y. 32422645

[ref16] Ganguly K.; Chaturvedi K.; More U. A.; Nadagouda M. N.; Aminabhavi T. M. Polysaccharide-Based Micro/Nanohydrogels for Delivering Macromolecular Therapeutics. J. Controlled Release 2014, 193, 162–173. 10.1016/j.jconrel.2014.05.014. 24845128

[ref17] Tabata Y.; Ikada Y. Protein Release from Gelatin Matrices. Adv. Drug Delivery Rev. 1998, 31 (3), 287–301. 10.1016/S0169-409X(97)00125-7. 10837630

[ref18] Hoffman A. S. Hydrogels for Biomedical Applications. Adv. Drug Delivery Rev. 2012, 64 (SUPPL), 18–23. 10.1016/j.addr.2012.09.010. 11755703

[ref19] Sharma S.; Tiwari S. A Review on Biomacromolecular Hydrogel Classification and Its Applications. Int. J. Biol. Macromol. 2020, 162, 737–747. 10.1016/j.ijbiomac.2020.06.110. 32553961

[ref20] Cao J.; Cai Y.; Yu L.; Zhou J. Dual Physically Crosslinked Hydrogels Based on the Synergistic Effects of Electrostatic and Dipole-Dipole Interactions. J. Mater. Chem. B 2019, 7 (4), 676–683. 10.1039/C8TB03032D. 32254800

[ref21] Fredrick R.; Podder A.; Viswanathan A.; Bhuniya S. Synthesis and Characterization of Polysaccharide Hydrogel Based on Hydrophobic Interactions. J. Appl. Polym. Sci. 2019, 136 (25), 47665 10.1002/app.47665.

[ref22] Peng G.; Wang J.; Yang F.; Zhang S.; Hou J.; Xing W.; Lu X.; Liu C. In Situ Formation of Biodegradable Dextran-Based Hydrogel via Michael Addition. J. Appl. Polym. Sci. 2013, 127 (1), 577–584. 10.1002/app.37825.

[ref23] Pasqui D.; De Cagna M.; Barbucci R. Polysaccharide-Based Hydrogels: The Key Role of Water in Affecting Mechanical Properties. Polymers (Basel, Switz.) 2012, 4 (3), 1517–1534. 10.3390/polym4031517.

[ref24] Dragan E. S.; Dinu M. V. Polysaccharides Constructed Hydrogels as Vehicles for Proteins and Peptides. A Review. Carbohydr. Polym. 2019, 225 (May), 115210 10.1016/j.carbpol.2019.115210. 31521316

[ref25] Hu Y.; Dong X.; Ke L.; Zhang S.; Zhao D.; Chen H.; Xiao X. Polysaccharides/Mesoporous Silica Nanoparticles Hybrid Composite Hydrogel Beads for Sustained Drug Delivery. J. Mater. Sci. 2017, 52 (6), 3095–3109. 10.1007/s10853-016-0597-x.

[ref26] Wang K.; Nune K. C.; Misra R. D. K. The Functional Response of Alginate-Gelatin-Nanocrystalline Cellulose Injectable Hydrogels toward Delivery of Cells and Bioactive Molecules. Acta Biomater. 2016, 36, 143–151. 10.1016/j.actbio.2016.03.016. 26971665

[ref27] Peppas N. A.; Bures P.; Leobandung W.; Ichikawa H. Hydrogels in Pharmaceutical Formulations. Eur. J. Pharm. Biopharm. 2000, 50 (1), 27–46. 10.1016/S0939-6411(00)00090-4. 10840191

[ref28] Wang N. X.; Von Recum H. A. Affinity-Based Drug Delivery. Macromol. Biosci. 2011, 11 (3), 321–332. 10.1002/mabi.201000206. 21108454

[ref29] Hoare T. R.; Kohane D. S. Hydrogels in Drug Delivery: Progress and Challenges. Polymer 2008, 49 (8), 1993–2007. 10.1016/j.polymer.2008.01.027.

[ref30] Chambin O.; Dupuis G.; Champion D.; Voilley A.; Pourcelot Y. Int. J. Pharm. 2006, 321, 86–93. 10.1016/j.ijpharm.2006.05.015. 16790326

[ref31] Levalley P. J.; Sutherland B. P.; Jaje J.; Gibbs S.; Jones R. M.; Gala R. P.; Kloxin C. J.; Kiick K. L.; Kloxin A. M. On-Demand and Tunable Dual Wavelength Release of Antibodies Using Light-Responsive Hydrogels. ACS Appl. Bio Mater. 2020, 3 (10), 6944–6958. 10.1021/acsabm.0c00823. PMC8315695 34327309

[ref32] Rizwan M.; Yahya R.; Hassan A.; Yar M.; Azzahari A.; Selvanathan V.; Sonsudin F.; Abouloula C. pH Sensitive Hydrogels in Drug Delivery : Brief History, Properties, Swelling, and Release Mechanism, Material Selection and Applications. Polymers (Basel, Switz.) 2017, 9 (4), 137 10.3390/polym9040137. PMC6432076 30970818

[ref33] Zhao W.; Odelius K.; Edlund U.; Zhao C.; Albertsson A. In Situ Synthesis of Magnetic Field-Responsive Hemicellulose Hydrogels for Drug Delivery. Biomacromolecules 2015, 16, 2522–2528. 10.1021/acs.biomac.5b00801. 26196600 PMC4535706

[ref34] Murdan S. Electro-Responsive Drug Delivery from Hydrogels. J. Controlled Release 2003, 92 (1–2), 1–17. 10.1016/S0168-3659(03)00303-1. 14499181

[ref35] Wei W.; Li J.; Qi X.; Zhong Y.; Zuo G.; Pan X.; Su T.; Zhang J.; Dong W. Synthesis and Characterization of a Multi-Sensitive Polysaccharide Hydrogel for Drug Delivery. Carbohydr. Polym. 2017, 177 (April), 275–283. 10.1016/j.carbpol.2017.08.133. 28962769

[ref36] Yang S.; Tang Z.; Zhang D.; Deng M.; Chen X. PH and REDOX Dual-Sensitive Polysaccharide Nanoparticles for Efficient Delivery of Doxorubicin. Biomater. Sci. 2017, 5 (10), 2169–2178. 10.1039/C7BM00632B. 28914292

[ref37] Knipe J. M.; Chen F.; Peppas N. A. Enzymatic Biodegradation of Hydrogels for Protein Delivery Targeted to the Small Intestine. Biomacromolecules 2015, 16, 962–972. 10.1021/bm501871a. 25674922

[ref38] Alvarez-Lorenzo C.; Blanco-Fernandez B.; Puga A. M.; Concheiro A. Crosslinked Ionic Polysaccharides for Stimuli-Sensitive Drug Delivery. Adv. Drug Delivery Rev. 2013, 65 (9), 1148–1171. 10.1016/j.addr.2013.04.016. 23639519

[ref39] Koetting M. C.; Peters J. T.; Steichen S. D.; Peppas N. A. Stimulus-Responsive Hydrogels: Theory, Modern Advances, and Applications. Mater. Sci. Eng., R 2015, 93, 1–49. 10.1016/j.mser.2015.04.001. PMC4847551 27134415

[ref40] Lavrador P.; Esteves M. R.; Gaspar V. M.; Mano J. F. Stimuli-Responsive Nanocomposite Hydrogels for Biomedical Applications. Adv. Funct. Mater. 2021, 31 (8), 2005941 10.1002/adfm.202005941.

[ref41] de Lima C. S. A.; Balogh T. S.; Varca J. P. R. O.; Varca G. H. C.; Lugão A. B.; Camacho-Cruz L. A.; Bucio E.; Kadlubowski S. S. An Updated Review of Macro, Micro, and Nanostructured Hydrogels for Biomedical and Pharmaceutical Applications. Pharmaceutics 2020, 12 (10), 970 10.3390/pharmaceutics12100970. PMC7602430 33076231

[ref42] Vulic K.; Shoichet M. S. Affinity-Based Drug Delivery Systems for Tissue Repair and Regeneration. Biomacromolecules 2014, 15 (11), 3867–3880. 10.1021/bm501084u. 25230248

[ref43] Li S.; Dong S.; Xu W.; Tu S.; Yan L.; Zhao C.; Ding J.; Chen X. Antibacterial Hydrogels. Adv. Sci. 2018, 5 (5), 1700527 10.1002/advs.201700527. PMC5980143 29876202

[ref44] Jeon O.; Alt D. S.; Ahmed S. M.; Alsberg E. The Effect of Oxidation on the Degradation of Photocrosslinkable Alginate Hydrogels. Biomaterials 2012, 33 (13), 3503–3514. 10.1016/j.biomaterials.2012.01.041. 22336294 PMC3593072

[ref45] Braccini I.; Pérez S. Molecular Basis of Ca2+-Induced Gelation in Alginates and Pectins: The Egg-Box Model Revisited. Biomacromolecules 2001, 2 (4), 1089–1096. 10.1021/bm010008g. 11777378

[ref46] Lee K. Y.; Yuk S. H. Polymeric Protein Delivery Systems. Prog. Polym. Sci. 2007, 32 (7), 669–697. 10.1016/j.progpolymsci.2007.04.001.

[ref47] Schweizer D.; Vostiar I.; Heier A.; Serno T.; Schoenhammer K.; Jahn M.; Jones S.; Piequet A.; Beerli C.; Gram H.; Goepferich A. Pharmacokinetics, Biocompatibility and Bioavailability of a Controlled Release Monoclonal Antibody Formulation. J. Controlled Release 2013, 172 (3), 975–982. 10.1016/j.jconrel.2013.10.010. 24140353

[ref48] Zhang Z.; Zhang R.; McClements D. J. Control of Protein Digestion under Simulated Gastrointestinal Conditions Using Biopolymer Microgels. Food Res. Int. 2017, 100 (2), 86–94. 10.1016/j.foodres.2017.08.037. 28888462

[ref49] Ferreira N. N.; M. B. Ferreira L.; Miranda-Goncalves V.; Reis R. M.; Seraphim T. V.; Borges J. C.; Baltazar F.; Gremiao M. P. D. Alginate Hydrogel Improves Anti-Angiogenic Bevacizumab Activity in Cancer Therapy. Eur. J. Pharm. Biopharm. 2017, 119, 271–282. 10.1016/j.ejpb.2017.06.028. 28669796

[ref50] Schweizer D.; Schönhammer K.; Jahn M.; Göpferich A. Protein-Polyanion Interactions for the Controlled Release of Monoclonal Antibodies. Biomacromolecules 2013, 14 (1), 75–83. 10.1021/bm301352x. 23157419

[ref51] Raimondo T. M.; Li H.; Kwee B. J.; Kinsley S.; Budina E.; Anderson E. M.; Doherty E. J.; Talbot S. G.; Mooney D. J. Biomaterials Combined Delivery of VEGF and IGF-1 Promotes Functional Innervation in Mice and Improves Muscle Transplantation in Rabbits. Biomaterials 2019, 216 (May), 119246 10.1016/j.biomaterials.2019.119246. 31203034

[ref52] Emi T.; Michaud K.; Orton E.; Santilli G.; Linh C.; O’Connell M.; Issa F.; Kennedy S. Ultrasonic Generation of Pulsatile and Sequential Therapeutic Delivery Profiles from Calcium-Crosslinked Alginate Hydrogels. Molecules 2019, 24 (6), 1048 10.3390/molecules24061048. PMC6470886 30884862

[ref53] Huebsch N.; Kearney C. J.; Zhao X.; Kim J.; Cezar C. A.; Suo Z.; Mooney D. J. Ultrasound-Triggered Disruption and Self-Healing of Reversibly Cross-Linked Hydrogels for Drug Delivery and Enhanced Chemotherapy. Proc. Natl. Acad. Sci. U. S. A. 2014, 111 (27), 9762–9767. 10.1073/pnas.1405469111. 24961369 PMC4103344

[ref54] Park J.; Lee S. J.; Lee H.; Park S. A; Lee J. Y. Three Dimensional Cell Printing with Sulfated Alginate for Improved Bone Morphogenetic Protein-2 Delivery and Osteogenesis in Bone Tissue Engineering. Carbohydr. Polym. 2018, 196, 217–224. 10.1016/j.carbpol.2018.05.048. 29891290

[ref55] Jung S. W.; Oh S. H.; Lee I. S.; Byun J. H.; Lee J. H. In Situ Gelling Hydrogel with Anti-Bacterial Activity and Bone Healing Property for Treatment of Osteomyelitis. Tissue Eng. Regener. Med. 2019, 16 (5), 479–490. 10.1007/s13770-019-00206-x. PMC6778575 31624703

[ref56] Ruehle M. A.; Li M. T. A.; Cheng A.; Krishnan L.; Willett N. J.; Guldberg R. E. Decorin-Supplemented Collagen Hydrogels for the Co-Delivery of Bone Morphogenetic Protein-2 and Microvascular Fragments to a Composite Bone-Muscle Injury Model with Impaired Vascularization. Acta Biomater. 2019, 93, 210–221. 10.1016/j.actbio.2019.01.045. 30685477 PMC6759335

[ref57] Hu Y.; Zhang Z.; Li Y.; Ding X.; Li D.; Shen C.; Xu F. J. Dual-Crosslinked Amorphous Polysaccharide Hydrogels Based on Chitosan/Alginate for Wound Healing Applications. Macromol. Rapid Commun. 2018, 39 (20), 1800069 10.1002/marc.201800069. 29855096

[ref58] Cikrikci S.; Mert B.; Oztop M. H. Development of PH Sensitive Alginate/Gum Tragacanth Based Hydrogels for Oral Insulin Delivery. J. Agric. Food Chem. 2018, 66 (44), 11784–11796. 10.1021/acs.jafc.8b02525. 30346766

[ref59] Déat-Lainé E.; Hoffart V.; Garrait G.; Jarrige J. F.; Cardot J. M.; Subirade M.; Beyssac E. Efficacy of Mucoadhesive Hydrogel Microparticles of Whey Protein and Alginate for Oral Insulin Delivery. Pharm. Res. 2013, 30 (3), 721–734. 10.1007/s11095-012-0913-3. 23093377

[ref60] Kim J. H.; Park S.; Kim H.; Kim H. J.; Yang Y. H.; Kim Y. H.; Jung S. K.; Kan E.; Lee S. H. Alginate/Bacterial Cellulose Nanocomposite Beads Prepared Using Gluconacetobacter Xylinus and Their Application in Lipase Immobilization. Carbohydr. Polym. 2017, 157, 137–145. 10.1016/j.carbpol.2016.09.074. 27987845

[ref61] Choi Y.; Kim S.; Kim I.; Lee J.; Kwon S. Injectable Basic Fibroblast Growth Factor-Loaded Alginate/Hyaluronic Acid Hydrogel for Rejuvenation of Geriatric Larynx. Acta Biomater. 2019, 89, 104–114. 10.1016/j.actbio.2019.03.005. 30849562

[ref62] Kim I. G.; Park M. R.; Choi Y. H.; Choi J. S.; Ahn H. J.; Kwon S. K.; Lee J. H. Regeneration of Paralyzed Vocal Fold by the Injection of Plasmid DNA Complex-Loaded Hydrogel Bulking Agent. ACS Biomater. Sci. Eng. 2019, 5 (3), 1497–1508. 10.1021/acsbiomaterials.8b01541. 33405624

[ref63] Zhai Z.; Xu K.; Mei L.; Wu C.; Liu J.; Liu Z.; Wan L.; Zhong W. Co-Assembled Supramolecular Hydrogels of Cell Adhesive Peptide and Alginate for Rapid Hemostasis and Efficacious Wound Healing. Soft Matter 2019, 15 (42), 8603–8610. 10.1039/C9SM01296F. 31616890

[ref64] Xiao B.; Chen Q.; Zhang Z.; Wang L.; Kang Y.; Denning T.; Merlin D. TNFα Gene Silencing Mediated by Orally Targeted Nanoparticles Combined with Interleukin-22 for Synergistic Combination Therapy of Ulcerative Colitis. J. Controlled Release 2018, 287 (July), 235–246. 10.1016/j.jconrel.2018.08.021. PMC6482469 30107214

[ref65] Wu T.; Huang J.; Jiang Y.; Hu Y.; Ye X.; Liu D.; Chen J. Formation of Hydrogels Based on Chitosan/Alginate for the Delivery of Lysozyme and Their Antibacterial Activity. Food Chem. 2018, 240, 361–369. 10.1016/j.foodchem.2017.07.052. 28946284

[ref66] Fletcher N. A.; Krebs M. D. Sustained Delivery of Anti-VEGF from Injectable Hydrogel Systems Provides a Prolonged Decrease of Endothelial Cell Proliferation and Angiogenesis: In Vitro. RSC Adv. 2018, 8 (16), 8999–9005. 10.1039/C7RA13014G. PMC9078589 35539877

[ref67] Priddy L. B.; Chaudhuri O.; Stevens H. Y.; Krishnan L.; Uhrig B. A.; Willett N. J.; Guldberg R. E. Oxidized Alginate Hydrogels for Bone Morphogenetic Protein-2 Delivery in Long Bone Defects. Acta Biomater. 2014, 10 (10), 4390–4399. 10.1016/j.actbio.2014.06.015. 24954001 PMC4160396

[ref68] Xu X.; Weng Y.; Xu L.; Chen H. Sustained Release of Avastin® from Polysaccharides Cross-Linked Hydrogels for Ocular Drug Delivery. Int. J. Biol. Macromol. 2013, 60, 272–276. 10.1016/j.ijbiomac.2013.05.034. 23748006

[ref69] Madrigal J. L.; Sharma S. N.; Campbell K. T.; Stilhano R. S.; Gijsbers R.; Silva E. A. Microgels Produced Using Microfluidic On-Chip Polymer Blending for Controlled Released of VEGF Encoding Lentivectors. Acta Biomater. 2018, 69, 265–276. 10.1016/j.actbio.2018.01.013. 29398644 PMC6819130

[ref70] Formica F. A.; Cavalli E.; Broguiere N.; Zenobi-Wong M. Cell-Instructive Alginate Hydrogels Targeting RhoA. Bioconjugate Chem. 2018, 29 (9), 3042–3053. 10.1021/acs.bioconjchem.8b00436. 30125096

[ref71] Mano J. F. Stimuli-Responsive Polymeric Systems for Biomedical Applications. Adv. Eng. Mater. 2008, 10 (6), 515–527. 10.1002/adem.200700355.

[ref72] Zhao J.; Zhao X.; Guo B.; Ma P. X. Multifunctional Interpenetrating Polymer Network Hydrogels Based on Methacrylated Alginate for the Delivery of Small Molecule Drugs and Sustained Release of Protein. Biomacromolecules 2014, 15 (9), 3246–3252. 10.1021/bm5006257. 25102223

[ref73] Chalanqui M. J.; Pentlavalli S.; McCrudden C.; Chambers P.; Ziminska M.; Dunne N.; McCarthy H. O. Influence of Alginate Backbone on Efficacy of Thermo-Responsive Alginate-g-P(NIPAAm) Hydrogel as a Vehicle for Sustained and Controlled Gene Delivery. Mater. Sci. Eng., C 2019, 95, 409–421. 10.1016/j.msec.2017.09.003. 30573265

[ref74] Segredo-Morales E.; García-García P.; Reyes R.; Pérez-Herrero E.; Delgado A.; Évora C. Bone Regeneration in Osteoporosis by Delivery BMP-2 and PRGF from Tetronic–Alginate Composite Thermogel. Int. J. Pharm. 2018, 543 (1–2), 160–168. 10.1016/j.ijpharm.2018.03.034. 29567197

[ref75] Heo Y.; Akimoto J.; Kobatake E.; Ito Y. Gelation and Release Behavior of Visible Light-Curable Alginate. Polym. J. 2020, 52 (3), 323–332. 10.1038/s41428-019-0280-6.

[ref76] Feng J.; Wu Y.; Chen W.; Li J.; Wang X.; Chen Y.; Yu Y.; Shen Z.; Zhang Y. Sustained Release of Bioactive IGF-1 from a Silk Fibroin Microsphere-Based Injectable Alginate Hydrogel for the Treatment of Myocardial Infarction. J. Mater. Chem. B 2020, 8 (2), 308–315. 10.1039/C9TB01971E. 31808500

[ref77] Yuan P.; Qiu X.; Jin R.; Bai Y.; Liu S.; Chen X. One-Pot Preparation of Polymer Microspheres with Different Porous Structures to Sequentially Release Bio-Molecules for Cutaneous Regeneration. Biomater. Sci. 2018, 6 (4), 820–826. 10.1039/C7BM00993C. 29461560

[ref78] Luo Z.; Zhang S.; Pan J.; Shi R.; Liu H.; Lyu Y.; Han X.; Li Y.; Yang Y.; Xu Z.; Sui Y.; Luo E.; Zhang Y.; Wei S. Time-Responsive Osteogenic Niche of Stem Cells: A Sequentially Triggered, Dual-Peptide Loaded, Alginate Hybrid System for Promoting Cell Activity and Osteo-Differentiation. Biomaterials 2018, 163, 25–42. 10.1016/j.biomaterials.2018.02.025. 29452946

[ref79] Zarrintaj P.; Manouchehri S.; Ahmadi Z.; Saeb M. R.; Urbanska A. M.; Kaplan D. L.; Mozafari M. Agarose-Based Biomaterials for Tissue Engineering. Carbohydr. Polym. 2018, 187, 66–84. 10.1016/j.carbpol.2018.01.060. 29486846

[ref80] Graham S.; Marina P. F.; Blencowe A. Thermoresponsive Polysaccharides and Their Thermoreversible Physical Hydrogel Networks. Carbohydr. Polym. 2019, 207, 143–159. 10.1016/j.carbpol.2018.11.053. 30599994

[ref81] Huynh V.; Wylie R. G. Competitive Affinity Release for Long-Term Delivery of Antibodies from Hydrogels. Angew. Chem., Int. Ed. 2018, 57 (13), 3406–3410. 10.1002/anie.201713428. 29392857

[ref82] Costa R. R.; Mano J. F. Polyelectrolyte Multilayered Assemblies in Biomedical Technologies. Chem. Soc. Rev. 2014, 43 (10), 3453–3479. 10.1039/c3cs60393h. 24549278

[ref83] Cardoso M. J.; Caridade S. G.; Costa R. R.; Mano J. F. Enzymatic Degradation of Polysaccharide-Based Layer-by-Layer Structures. Biomacromolecules 2016, 17 (4), 1347–1357. 10.1021/acs.biomac.5b01742. 26957012

[ref84] Mehrotra S.; Lynam D.; Maloney R.; Pawelec K. M.; Tuszynski M. H.; Lee I.; Chan C.; Sakamoto J. Time Controlled Protein Release from Layer-by-Layer Assembled Multilayer Functionalized Agarose Hydrogels. Adv. Funct. Mater. 2010, 20 (2), 247–258. 10.1002/adfm.200901172. 20200599 PMC2830720

[ref85] Lynam D.; Peterson C.; Maloney R.; Shahriari D.; Garrison A.; Saleh S.; Mehrotra S.; Chan C.; Sakamoto J. Augmenting Protein Release from Layer-by-Layer Functionalized Agarose Hydrogels. Carbohydr. Polym. 2014, 103 (1), 377–384. 10.1016/j.carbpol.2013.12.069. 24528743 PMC3986118

[ref86] Ahearne M.; Kelly D. J. A Comparison of Fibrin, Agarose and Gellan Gum Hydrogels as Carriers of Stem Cells and Growth Factor Delivery Microspheres for Cartilage Regeneration. Biomed. Mater. 2013, 8 (3), 035004 10.1088/1748-6041/8/3/035004. 23532058

[ref87] Moghassemi S.; Hadjizadeh A. Nano-Niosomes as Nanoscale Drug Delivery Systems: An Illustrated Review. J. Controlled Release 2014, 185 (1), 22–36. 10.1016/j.jconrel.2014.04.015. 24747765

[ref88] Moghassemi S.; Hadjizadeh A.; Hakamivala A.; Omidfar K. Growth Factor-Loaded Nano-Niosomal Gel Formulation and Characterization. AAPS PharmSciTech 2017, 18 (1), 34–41. 10.1208/s12249-016-0579-y. 27502406

[ref89] Bhattarai N.; Gunn J.; Zhang M. Chitosan-Based Hydrogels for Controlled, Localized Drug Delivery. Adv. Drug Delivery Rev. 2010, 62 (1), 83–99. 10.1016/j.addr.2009.07.019. 19799949

[ref90] Del Valle L.; Diaz A.; Puiggali J. Hydrogels for Biomedical Applications: Cellulose, Chitosan, and Protein/Peptide Derivatives. Gels 2017, 3 (3), 27 10.3390/gels3030027. PMC6318613 30920524

[ref91] Alves N. M.; Mano J. F. Chitosan Derivatives Obtained by Chemical Modifications for Biomedical and Environmental Applications. Int. J. Biol. Macromol. 2008, 43 (5), 401–414. 10.1016/j.ijbiomac.2008.09.007. 18838086

[ref92] Peers S.; Montembault A.; Ladavière C. Chitosan Hydrogels for Sustained Drug Delivery. J. Controlled Release 2020, 326 (June), 150–163. 10.1016/j.jconrel.2020.06.012. 32562854

[ref93] Sahranavard M.; Zamanian A.; Ghorbani F.; Shahrezaee M. H. A Critical Review on Three Dimensional-Printed Chitosan Hydrogels for Development of Tissue Engineering. Bioprinting 2020, 17, e00063 10.1016/j.bprint.2019.e00063.

[ref94] Oliveira M. B.; Bastos H. X. S.; Mano J. F. Sequentially Moldable and Bondable Four-Dimensional Hydrogels Compatible with Cell Encapsulation. Biomacromolecules 2018, 19 (7), 2742–2749. 10.1021/acs.biomac.8b00337. 29698598 PMC6450509

[ref95] Moreno M.; Pow P. Y.; Tabitha T. S. T.; Nirmal S.; Larsson A.; Radhakrishnan K.; Nirmal J.; Quah S. T.; Geifman Shochat S.; Agrawal R.; Venkatraman S. Modulating Release of Ranibizumab and Aflibercept from Thiolated Chitosan-Based Hydrogels for Potential Treatment of Ocular Neovascularization. Expert Opin. Drug Delivery 2017, 14 (8), 913–925. 10.1080/17425247.2017.1343297. 28643528

[ref96] Yang L. Q.; Lan Y. Q.; Guo H.; Cheng L. Z.; Fan J. Z.; Cai X.; Zhang L. M.; Chen R. F.; Zhou H. S. Ophthalmic Drug-Loaded N,O-Carboxymethyl Chitosan Hydrogels: Synthesis, in Vitro and in Vivo Evaluation. Acta Pharmacol. Sin. 2010, 31 (12), 1625–1634. 10.1038/aps.2010.125. 21042284 PMC4002939

[ref97] Erickson C. B.; Newsom J. P.; Fletcher N. A.; Yu Y.; Rodriguez-Fontan F.; Weatherford S. A.; Hadley-Miller N.; Krebs M. D.; Payne K. A. Anti-VEGF Antibody Delivered Locally Reduces Bony Bar Formation Following Physeal Injury in Rats. J. Orthop. Res. 2020, 1–11. 10.1002/jor.24907. 33179297

[ref98] Sukarto A.; Yu C.; Flynn L. E.; Amsden B. G. Co-Delivery of Adipose-Derived Stem Cells and Growth Factor-Loaded Microspheres in RGD-Grafted N-Methacrylate Glycol Chitosan Gels for Focal Chondral Repair. Biomacromolecules 2012, 13 (8), 2490–2502. 10.1021/bm300733n. 22746668

[ref99] Min Q.; Yu X.; Liu J.; Wu J.; Wan Y. Chitosan-Based Hydrogels Embedded with Hyaluronic Acid Complex Nanoparticles for Controlled Delivery of Bone Morphogenetic Protein-2. Pharmaceutics 2019, 11 (5), 214 10.3390/pharmaceutics11050214. PMC6572415 31060227

[ref100] Gohil S. V.; Wang L.; Rowe D. W.; Nair L. S. Spatially Controlled RhBMP-2 Mediated Calvarial Bone Formation in a Transgenic Mouse Model. Int. J. Biol. Macromol. 2018, 106, 1159–1165. 10.1016/j.ijbiomac.2017.08.116. 28847606

[ref101] Naderi-Meshkin H.; Andreas K.; Matin M. M.; Sittinger M.; Bidkhori H. R.; Ahmadiankia N.; Bahrami A. R.; Ringe J. Chitosan-Based Injectable Hydrogel as a Promising in Situ Forming Scaffold for Cartilage Tissue Engineering. Cell Biol. Int. 2014, 38 (1), 72–84. 10.1002/cbin.10181. 24108671

[ref102] Kim J.; Lin B.; Kim S.; Choi B.; Evseenko D.; Lee M. TGF-B1 Conjugated Chitosan Collagen Hydrogels Induce Chondrogenic Differentiation of Human Synovium-Derived Stem Cells. J. Biol. Eng. 2015, 9 (1), 1–11. 10.1186/1754-1611-9-1. 25745515 PMC4350967

[ref103] Shao J.; Ding Z.; Li L.; Chen Y.; Zhu J.; Qian Q. Improved Accumulation of TGF-β by Photopolymerized Chitosan/Silk Protein Bio-Hydrogel Matrix to Improve Differentiations of Mesenchymal Stem Cells in Articular Cartilage Tissue Regeneration. J. Photochem. Photobiol., B 2020, 203 (415), 111744 10.1016/j.jphotobiol.2019.111744. 31887637

[ref104] Lee Y. H.; Hong Y. L.; Wu T. L. Novel Silver and Nanoparticle-Encapsulated Growth Factor Co-Loaded Chitosan Composite Hydrogel with Sustained Antimicrobility and Promoted Biological Properties for Diabetic Wound Healing. Mater. Sci. Eng., C 2021, 118, 111385 10.1016/j.msec.2020.111385. 33254992

[ref105] Lim T.; Tang Q.; Zhu Z.; Wei X.; Zhang C. Sustained Release of Human Platelet Lysate Growth Factors by Thermosensitive Hydroxybutyl Chitosan Hydrogel Promotes Skin Wound Healing in Rats. J. Biomed. Mater. Res., Part A 2020, 108 (10), 2111–2122. 10.1002/jbm.a.36970. 32323472

[ref106] Yao Y.; Yang L.; Feng L. F.; Yue Z. W.; Zhao N. H.; Li Z.; He Z. X. IGF-1C Domain-Modified Hydrogel Enhanced the Efficacy of Stem Cells in the Treatment of AMI. Stem Cell Res. Ther. 2020, 11 (1), 1–14. 10.1186/s13287-020-01637-3. 32216819 PMC7098145

[ref107] Lopes M.; Simoes S.; Veiga F.; Seica R.; Ribeiro A. Why Most Oral Insulin Formulations Do Not Reach Clinical Trials Therapeutic Delivery Oral. Ther. Delivery 2015, 6, 973 10.4155/TDE.15.47. 26272222

[ref108] Ghasemi Tahrir F.; Ganji F.; Mani A. R.; Khodaverdi E. In Vitro and in Vivo Evaluation of Thermosensitive Chitosan Hydrogel for Sustained Release of Insulin. Drug Delivery 2016, 23 (3), 1028–1036. 10.3109/10717544.2014.932861. 25005583

[ref109] Zu Y.; Zhang Y.; Zhao X.; Shan C.; Zu S.; Wang K.; Li Y.; Ge Y. Preparation and Characterization of Chitosan-Polyvinyl Alcohol Blend Hydrogels for the Controlled Release of Nano-Insulin. Int. J. Biol. Macromol. 2012, 50 (1), 82–87. 10.1016/j.ijbiomac.2011.10.006. 22020189

[ref110] Peng Q.; Sun X.; Gong T.; Wu C. Y.; Zhang T.; Tan J.; Zhang Z. R. Injectable and Biodegradable Thermosensitive Hydrogels Loaded with PHBHHx Nanoparticles for the Sustained and Controlled Release of Insulin. Acta Biomater. 2013, 9 (2), 5063–5069. 10.1016/j.actbio.2012.09.034. 23036950

[ref111] Li Z.; Li H.; Wang C.; Xu J.; Singh V.; Chen D.; Zhang J. Sodium Dodecyl Sulfate/β-Cyclodextrin Vesicles Embedded in Chitosan Gel for Insulin Delivery with PH-Selective Release. Acta Pharm. Sin. B 2016, 6 (4), 344–351. 10.1016/j.apsb.2016.03.003. 27471675 PMC4951593

[ref112] Wen N.; Lü S.; Xu X.; Ning P.; Wang Z.; Zhang Z.; Gao C.; Liu Y.; Liu M. A Polysaccharide-Based Micelle-Hydrogel Synergistic Therapy System for Diabetes and Vascular Diabetes Complications Treatment. Mater. Sci. Eng., C 2019, 100, 94–103. 10.1016/j.msec.2019.02.081. 30948130

[ref113] Yin R.; He J.; Bai M.; Huang C.; Wang K.; Zhang H.; Yang S. M.; Zhang W. Engineering Synthetic Artificial Pancreas Using Chitosan Hydrogels Integrated with Glucose-Responsive Microspheres for Insulin Delivery. Mater. Sci. Eng., C 2019, 96, 374–382. 10.1016/j.msec.2018.11.032. 30606545

[ref114] Khodaverdi E.; Tafaghodi M.; Ganji F.; Abnoos K.; Naghizadeh H. In Vitro Insulin Release from Thermosensitive Chitosan Hydrogel. AAPS PharmSciTech 2012, 13 (2), 460–466. 10.1208/s12249-012-9764-9. 22391886 PMC3364382

[ref115] Li Y.; He J.; Lyu X.; Yuan Y.; Wang G.; Zhao B. Chitosan-Based Thermosensitive Hydrogel for Nasal Delivery of Exenatide: Effect of Magnesium Chloride. Int. J. Pharm. 2018, 553 (1–2), 375–385. 10.1016/j.ijpharm.2018.10.071. 30389472

[ref116] Wang X.; Guo W.; Li L.; Yu F.; Li J.; Liu L.; Fang B.; Xia L. Photothermally Triggered Biomimetic Drug Delivery of Teriparatide via Reduced Graphene Oxide Loaded Chitosan Hydrogel for Osteoporotic Bone Regeneration. Chem. Eng. J. 2021, 413, 127413 10.1016/j.cej.2020.127413.

[ref117] Eicher A. C.; Dobler D.; Kiselmann C.; Schmidts T.; Runkel F. Dermal Delivery of Therapeutic DNAzymes via Chitosan Hydrogels. Int. J. Pharm. 2019, 563 (March), 208–216. 10.1016/j.ijpharm.2019.04.005. 30953763

[ref118] McMahon S. S.; Nikolskaya N.; Choileáin S. N.; Hennessy N.; O’Brien T.; Strappe P. M.; Gorelov A.; Rochev Y. Thermosensitive Hydrogel for Prolonged Delivery of Lentiviral Vector Expressing Neurotrophin-3 in Vitro. J. Gene Med. 2011, 13 (11), 591–601. 10.1002/jgm.1613. 21954128

[ref119] Ma Z.; Yang C.; Song W.; Wang Q.; Kjems J.; Gao S. Chitosan Hydrogel as Sirna Vector for Prolonged Gene Silencing. J. Nanobiotechnol. 2014, 12 (1), 23 10.1186/1477-3155-12-23. PMC4104730 24946934

[ref120] Cao C.; Yan C.; Hu Z.; Zhou S. Potential Application of Injectable Chitosan Hydrogel Treated with SiRNA in Chronic Rhinosinusitis Therapy. Mol. Med. Rep. 2015, 12 (5), 6688–6694. 10.3892/mmr.2015.4237. 26299569 PMC4626163

[ref121] Choi B.; Cui Z.-K.; Kim S.; Fan J.; Wu B. M.; Lee M. Glutamine-Chitosan Modified Calcium Phosphate Nanoparticles for Efficient SiRNA Delivery and Osteogenic Differentiation. J. Mater. Chem. B 2015, 3 (31), 6448–6455. 10.1039/C5TB00843C. 26413302 PMC4582691

[ref122] Han H. D.; Mora E. M.; Roh J. W.; Nishimura M.; Lee S. J.; Stone R. L.; Bar-Eli M.; Lopez-Berestein G.; Sood A. K. Chitosan Hydrogel for Localized Gene Silencing. Cancer Biol. Ther. 2011, 11 (9), 839–845. 10.4161/cbt.11.9.15185. 21358280 PMC3100632

[ref123] Yegappan R.; Selvaprithiviraj V.; Amirthalingam S.; Jayakumar R. Carrageenan Based Hydrogels for Drug Delivery, Tissue Engineering and Wound Healing. Carbohydr. Polym. 2018, 198, 385–400. 10.1016/j.carbpol.2018.06.086. 30093014

[ref124] Mihaila S. M.; Gaharwar A. K.; Reis R. L.; Marques A. P.; Gomes M. E.; Khademhosseini A. Photocrosslinkable Kappa-Carrageenan Hydrogels for Tissue Engineering Applications. Adv. Healthcare Mater. 2013, 2 (6), 895–907. 10.1002/adhm.201200317. 23281344

[ref125] Qureshi D.; Nayak S. K.; Maji S.; Kim D.; Banerjee I.; Pal K. Carrageenan: A Wonder Polymer from Marine Algae for Potential Drug Delivery Applications. Curr. Pharm. Des. 2019, 25 (11), 1172–1186. 10.2174/1381612825666190425190754. 31465278

[ref126] Qi X.; Su T.; Zhang M.; Tong X.; Pan W.; Zeng Q.; Zhou Z.; Shen L.; He X.; Shen J. Macroporous Hydrogel Scaffolds with Tunable Physicochemical Properties for Tissue Engineering Constructed Using Renewable Polysaccharides. ACS Appl. Mater. Interfaces 2020, 12, 13256–13264. 10.1021/acsami.9b20794. 32068392

[ref127] Wang L.; Cao J.; Lei D. L.; Cheng X. B.; Zhou H. Z.; Hou R.; Zhao Y. H.; Cui F. Z. Application of Nerve Growth Factor by Gel Increases Formation of Bone in Mandibular Distraction Osteogenesis in Rabbits. Br. J. Oral Maxillofac. Surg. 2010, 48 (7), 515–519. 10.1016/j.bjoms.2009.08.042. 20236741

[ref128] Rocha P. M.; Santo V. E.; Gomes M. E.; Reis R. L.; Mano J. F. Encapsulation of Adipose-Derived Stem Cells and Transforming Growth Factor-B1 in Carrageenan-Based Hydrogels for Cartilage Tissue Engineering. J. Bioact. Compat. Polym. 2011, 26 (5), 493–507. 10.1177/0883911511420700.

[ref129] Mohammadinejad R.; Maleki H.; Larrañeta E.; Fajardo A. R.; Nik A. B.; Shavandi A.; Sheikhi A.; Ghorbanpour M.; Farokhi M.; Govindh P.; Cabane E.; Azizi S.; Aref A. R.; Mozafari M.; Mehrali M.; Thomas S.; Mano J. F.; Mishra Y. K.; Thakur V. K. Status and Future Scope of Plant-Based Green Hydrogels in Biomedical Engineering. Appl. Mater. Today 2019, 16, 213–246. 10.1016/j.apmt.2019.04.010.

[ref130] Sun T.; Zhu C.; Xu J. Multiple Stimuli-Responsive Selenium-Functionalized Biodegradable Starch-Based Hydrogels. Soft Matter 2018, 14 (6), 921–926. 10.1039/C7SM02137B. 29309083

[ref131] Leloup V. M.; Colonna P.; Buleon A. Influence of Amylose-Amylopectin Ratio on Gel Properties. J. Cereal Sci. 1991, 13 (1), 1–13. 10.1016/S0733-5210(09)80023-4.

[ref132] Biduski B.; Silva W. M. F. d.; Colussi R.; Halal S. L. d. M. E.; Lim L.-T.; Dias A. R. G.; Zavareze E. d. R. Starch Hydrogels: The Influence of the Amylose Content and Gelatinization Method. Int. J. Biol. Macromol. 2018, 113, 443–449. 10.1016/j.ijbiomac.2018.02.144. 29486261

[ref133] Setty C. M.; Deshmukh A. S.; Badiger A. M. Hydrolyzed Polyacrylamide Grafted Carboxymethylxyloglucan Based Microbeads for PH Responsive Drug Delivery. Int. J. Biol. Macromol. 2014, 67, 28–36. 10.1016/j.ijbiomac.2014.03.005. 24632345

[ref134] Forouzandehdel S.; Forouzandehdel S.; Rezghi Rami M. Synthesis of a Novel Magnetic Starch-Alginic Acid-Based Biomaterial for Drug Delivery. Carbohydr. Res. 2020, 487, 107889 10.1016/j.carres.2019.107889. 31841826

[ref135] Qi X.; Li Z.; Shen L.; Qin T.; Qian Y.; Zhao S.; Liu M.; Zeng Q.; Shen J. Highly Efficient Dye Decontamination via Microbial Salecan Polysaccharide-Based Gels. Carbohydr. Polym. 2019, 219, 1–11. 10.1016/j.carbpol.2019.05.021. 31151505

[ref136] Wöhl-Bruhn S.; Bertz A.; Harling S.; Menzel H.; Bunjes H. Hydroxyethyl Starch-Based Polymers for the Controlled Release of Biomacromolecules from Hydrogel Microspheres. Eur. J. Pharm. Biopharm. 2012, 81 (3), 573–581. 10.1016/j.ejpb.2012.04.017. 22579731

[ref137] Faikrua A.; Wittaya-Areekul S.; Oonkhanond B.; Viyoch J. In Vivo Chondrocyte and Transforming Growth Factor-B1 Delivery Using the Thermosensitive Chitosan/Starch/β-Glycerol Phosphate Hydrogel. J. Biomater. Appl. 2013, 28 (2), 175–186. 10.1177/0885328212441847. 22457042

[ref138] Komur B.; Akyuva Y.; Karaslan N.; Isyar M.; Gumustas S. A.; Yilmaz I.; Akkaya S.; Sirin D. Y.; Mutlu C. A.; Batmaz A. G.; Guler O.; Mahirogullari M. Can a Biodegradable Implanted Bilayered Drug Delivery System Loaded with BMP-2/BMP-12 Take an Effective Role in the Biological Repair Process of Bone-Tendon Injuries? A Preliminary Report. J. Pharm. 2017, 2017, 7457865 10.1155/2017/7457865. PMC5474233 28660091

[ref139] Moon H. C.; Han S.; Borges J.; Pesqueira T.; Choi H.; Han S. Y.; Cho H.; Park J. H.; Mano J. F.; Choi I. S. Enzymatically Degradable, Starch-Based Layer-by-Layer Films: Application to Cytocompatible Single-Cell Nanoencapsulation. Soft Matter 2020, 16 (26), 6063–6071. 10.1039/D0SM00876A. 32510086

[ref140] Dutta S. D.; Patel D. K.; Lim K.-T. Functional Cellulose-Based Hydrogels as Extracellular Matrices for Tissue Engineering. J. Biol. Eng. 2019, 13 (1), 55 10.1186/s13036-019-0177-0. 31249615 PMC6585131

[ref141] Aubeux D.; Beck L.; Weiss P.; Guicheux J.; Enkel B.; Pérez F.; Simon S. Assessment and Quantification of Noncollagenic Matrix Proteins Released from Human Dentin Powder Incorporated into a Silated Hydroxypropylmethylcellulose Biomedical Hydrogel. J. Endod. 2016, 42 (9), 1371–1376. 10.1016/j.joen.2016.05.019. 27430942

[ref142] Pakulska M. M.; Tator C. H.; Shoichet M. S. Local Delivery of Chondroitinase ABC with or without Stromal Cell-Derived Factor 1$α$ Promotes Functional Repair in the Injured Rat Spinal Cord. Biomaterials 2017, 134, 13–21. 10.1016/j.biomaterials.2017.04.016. 28453954

[ref143] Paukkonen H.; Kunnari M.; Laurén P.; Hakkarainen T.; Auvinen V.-V.; Oksanen T.; Koivuniemi R.; Yliperttula M.; Laaksonen T. Nanofibrillar Cellulose Hydrogels and Reconstructed Hydrogels as Matrices for Controlled Drug Release. Int. J. Pharm. 2017, 532 (1), 269–280. 10.1016/j.ijpharm.2017.09.002. 28888974

[ref144] Hettiaratchi M. H.; O’Meara M. J.; Teal C. J.; Payne S. L.; Pickering A. J.; Shoichet M. S. Local Delivery of Stabilized Chondroitinase ABC Degrades Chondroitin Sulfate Proteoglycans in Stroke-Injured Rat Brains. J. Controlled Release 2019, 297, 14–25. 10.1016/j.jconrel.2019.01.033. 30690102

[ref145] Chang C.; Zhang L. Cellulose-Based Hydrogels: Present Status and Application Prospects. Carbohydr. Polym. 2011, 84 (1), 40–53. 10.1016/j.carbpol.2010.12.023.

[ref146] Giusto G.; Vercelli C.; Comino F.; Caramello V.; Tursi M.; Gandini M. A New, Easy-to-Make Pectin-Honey Hydrogel Enhances Wound Healing in Rats. BMC Complementary Altern. Med. 2017, 17 (1), 266 10.1186/s12906-017-1769-1. PMC5433168 28511700

[ref147] Munarin F.; Tanzi M. C.; Petrini P. Advances in Biomedical Applications of Pectin Gels. Int. J. Biol. Macromol. 2012, 51 (4), 681–689. 10.1016/j.ijbiomac.2012.07.002. 22776748

[ref148] Tan S.; Ladewig K.; Fu Q.; Blencowe A.; Qiao G. G. Cyclodextrin-Based Supramolecular Assemblies and Hydrogels: Recent Advances and Future Perspectives. Macromol. Rapid Commun. 2014, 35 (13), 1166–1184. 10.1002/marc.201400080. 24715693

[ref149] Ninan N.; Muthiah M.; Park I.-K.; Elain A.; Thomas S.; Grohens Y. Pectin/Carboxymethyl Cellulose/Microfibrillated Cellulose Composite Scaffolds for Tissue Engineering. Carbohydr. Polym. 2013, 98 (1), 877–885. 10.1016/j.carbpol.2013.06.067. 23987424

[ref150] Zhang X.; Kang X.; Ji L.; Bai J.; Liu W.; Wang Z. Stimulation of Wound Healing Using Bioinspired Hydrogels with Basic Fibroblast Growth Factor (BFGF). Int. J. Nanomed. 2018, 13, 3897–3906. 10.2147/IJN.S168998. PMC6038860 30013343

[ref151] Zhu Y.; Yao Z.; Liu Y.; Zhang W.; Geng L.; Ni T. Incorporation of ROS-Responsive Substance P-Loaded Zeolite Imidazolate Framework-8 Nanoparticles into a Ca2+-Cross-Linked Alginate/Pectin Hydrogel for Wound Dressing Applications. Int. J. Nanomed. 2020, 15, 333–346. 10.2147/IJN.S225197. PMC6980861 32021183

[ref152] Amirian J.; Linh N. T. B.; Min Y. K.; Lee B. T. Bone Formation of a Porous Gelatin-Pectin-Biphasic Calcium Phosphate Composite in Presence of BMP-2 and VEGF. Int. J. Biol. Macromol. 2015, 76, 10–24. 10.1016/j.ijbiomac.2015.02.021. 25709009

[ref153] Shen X.; Liu L.; Peek R. M.; Acra S. A.; Moore D. J.; Wilson K. T.; He F.; Polk D. B.; Yan F. Supplementation of P40, a Lactobacillus Rhamnosus GG-Derived Protein, in Early Life Promotes Epidermal Growth Factor Receptor-Dependent Intestinal Development and Long-Term Health Outcomes. Mucosal Immunol. 2018, 11 (5), 1316–1328. 10.1038/s41385-018-0034-3. 29875401 PMC6162144

[ref154] Van Tomme S. R.; Hennink W. E. Biodegradable Dextran Hydrogels for Protein Delivery Applications. Expert Rev. Med. Devices 2007, 4 (2), 147–164. 10.1586/17434440.4.2.147. 17359222

[ref155] Brunsen A.; Ritz U.; Mateescu A.; Höfer I.; Frank P.; Menges B.; Hofmann A.; Rommens P. M.; Knoll W.; Jonas U. Photocrosslinkable Dextran Hydrogel Films as Substrates for Osteoblast and Endothelial Cell Growth. J. Mater. Chem. 2012, 22 (37), 19590–19604. 10.1039/c2jm34006b.

[ref156] Wei Z.; Gerecht S. A Self-Healing Hydrogel as an Injectable Instructive Carrier for Cellular Morphogenesis. Biomaterials 2018, 185 (April), 86–96. 10.1016/j.biomaterials.2018.09.003. 30236839 PMC6432635

[ref157] Jin R.; Moreira Teixeira L. S.; Dijkstra P. J.; van Blitterswijk C. A.; Karperien M.; Feijen J. Enzymatically-Crosslinked Injectable Hydrogels Based on Biomimetic Dextran-Hyaluronic Acid Conjugates for Cartilage Tissue Engineering. Biomaterials 2010, 31 (11), 3103–3113. 10.1016/j.biomaterials.2010.01.013. 20116847

[ref158] Hennink W. E.; De Jong S. J.; Bos G. W.; Veldhuis T. F. J.; Van Nostrum C. F. Biodegradable Dextran Hydrogels Crosslinked by Stereocomplex Formation for the Controlled Release of Pharmaceutical Proteins. Int. J. Pharm. 2004, 277 (1–2), 99–104. 10.1016/j.ijpharm.2003.02.002. 15158973

[ref159] Meyvis T.; De Smedt S.; Stubbe B.; Hennink W.; Demeester J. On the Release of Proteins from Degrading Dextran Methacrylate Hydrogels and the Correlation with the Rheologic Properties of the Hydrogels. Pharm. Res. 2001, 18 (11), 1593–1599. 10.1023/A:1013038716373. 11758768

[ref160] Pacelli S.; Paolicelli P.; Casadei M. A. New Biodegradable Dextran-Based Hydrogels for Protein Delivery: Synthesis and Characterization. Carbohydr. Polym. 2015, 126, 208–214. 10.1016/j.carbpol.2015.03.016. 25933541

[ref161] Cadée J. A.; De Groot C. J.; Jiskoot W.; Den Otter W.; Hennink W. E. Release of Recombinant Human Interleukin-2 from Dextran-Based Hydrogels. J. Controlled Release 2002, 78 (1–3), 1–13. 10.1016/S0168-3659(01)00483-7. 11772444

[ref162] Nguyen K.; Dang P. N.; Alsberg E. Functionalized, Biodegradable Hydrogels for Control over Sustained and Localized SiRNA Delivery to Incorporated and Surrounding Cells. Acta Biomater. 2013, 9 (1), 4487–4495. 10.1016/j.actbio.2012.08.012. 22902819 PMC3508156

[ref163] Hill M. C.; Nguyen M. K.; Jeon O.; Alsberg E. Spatial Control of Cell Gene Expression by SiRNA Gradients in Biodegradable Hydrogels. Adv. Healthcare Mater. 2015, 4 (5), 714–722. 10.1002/adhm.201400458. PMC4406766 25530099

[ref164] Merckx P.; De Backer L.; Van Hoecke L.; Guagliardo R.; Echaide M.; Baatsen P.; Olmeda B.; Saelens X.; Pérez-Gil J.; De Smedt S. C.; Raemdonck K. Surfactant Protein B (SP-B) Enhances the Cellular SiRNA Delivery of Proteolipid Coated Nanogels for Inhalation Therapy. Acta Biomater. 2018, 78, 236–246. 10.1016/j.actbio.2018.08.012. 30118853

[ref165] Raemdonck K.; Van Thienen T. G.; Vandenbroucke R. E.; Sanders N. N.; Demeester J.; De Smedt S. C. Dextran Microgels for Time-Controlled Delivery of SiRNA. Adv. Funct. Mater. 2008, 18 (7), 993–1001. 10.1002/adfm.200701039.

[ref166] Hiemstra C.; Van Der Aa L. J.; Zhong Z.; Dijkstra P. J.; Feijen J. Novel in Situ Forming, Degradable Dextran Hydrogels by Michael Addition Chemistry: Synthesis, Rheology, and Degradation. Macromolecules 2007, 40 (4), 1165–1173. 10.1021/ma062468d.

[ref167] Hiemstra C.; Zhong Z.; van Steenbergen M. J.; Hennink W. E.; Feijen J. Release of Model Proteins and Basic Fibroblast Growth Factor from in Situ Forming Degradable Dextran Hydrogels. J. Controlled Release 2007, 122 (1), 71–78. 10.1016/j.jconrel.2007.06.011. 17658651

[ref168] Nguyen M. K.; Huynh C. T.; Gilewski A.; Wilner S. E.; Maier K. E.; Kwon N.; Levy M.; Alsberg E. Covalently Tethering SiRNA to Hydrogels for Localized, Controlled Release and Gene Silencing. Sci. Adv. 2019, 5 (8), eaax0801 10.1126/sciadv.aax0801. 31489374 PMC6713499

[ref169] Nguyen M. K.; McMillan A.; Huynh C. T.; Schapira D. S.; Alsberg E. Photocrosslinkable, Biodegradable Hydrogels with Controlled Cell Adhesivity for Prolonged SiRNA Delivery to HMSCs to Enhance Their Osteogenic Differentiation. J. Mater. Chem. B 2017, 5 (3), 485–495. 10.1039/C6TB01739H. 28652917 PMC5482539

[ref170] Maia J.; Ferreira L.; Carvalho R.; Ramos M. A.; Gil M. H. Synthesis and Characterization of New Injectable and Degradable Dextran-Based Hydrogels. Polymer 2005, 46 (23), 9604–9614. 10.1016/j.polymer.2005.07.089.

[ref171] Ribeiro M. P.; Morgado P. I.; Miguel S. P.; Coutinho P.; Correia I. J. Dextran-Based Hydrogel Containing Chitosan Microparticles Loaded with Growth Factors to Be Used in Wound Healing. Mater. Sci. Eng., C 2013, 33 (5), 2958–2966. 10.1016/j.msec.2013.03.025. 23623119

[ref172] Draye J. P.; Delaey B.; Van De Voorde A.; Van Den Bulcke A.; Bogdanov B.; Schacht E. In Vitro Release Characteristics of Bioactive Molecules from Dextran Dialdehyde Cross-Linked Gelatin Hydrogel Films. Biomaterials 1998, 19 (1–3), 99–107. 10.1016/S0142-9612(97)00164-6. 9678856

[ref173] Chen M.; Tian J.; Liu Y.; Cao H.; Li R.; Wang J.; Wu J.; Zhang Q. Dynamic Covalent Constructed Self-Healing Hydrogel for Sequential Delivery of Antibacterial Agent and Growth Factor in Wound Healing. Chem. Eng. J. 2019, 373 (May), 413–424. 10.1016/j.cej.2019.05.043.

[ref174] Xu F.; Corbett B.; Bell S.; Zhang C.; Budi Hartono M.; Farsangi Z. J.; MacGregor J.; Hoare T. High-Throughput Synthesis, Analysis, and Optimization of Injectable Hydrogels for Protein Delivery. Biomacromolecules 2020, 21 (1), 214–229. 10.1021/acs.biomac.9b01132. 31686502

[ref175] Maire M.; Logeart-Avramoglou D.; Degat M. C.; Chaubet F. Retention of Transforming Growth Factor B1 Using Functionalized Dextran-Based Hydrogels. Biomaterials 2005, 26 (14), 1771–1780. 10.1016/j.biomaterials.2004.06.003. 15576151

[ref176] Maire M.; Chaubet F.; Mary P.; Blanchat C.; Meunier A.; Logeart-Avramoglou D. Bovine BMP Osteoinductive Potential Enhanced by Functionalized Dextran-Derived Hydrogels. Biomaterials 2005, 26 (24), 5085–5092. 10.1016/j.biomaterials.2005.01.020. 15769544

[ref177] Jin R.; Hiemstra C.; Zhong Z.; Feijen J. Enzyme-Mediated Fast in Situ Formation of Hydrogels from Dextran-Tyramine Conjugates. Biomaterials 2007, 28 (18), 2791–2800. 10.1016/j.biomaterials.2007.02.032. 17379300

[ref178] Bae K. H.; Lee F.; Xu K.; Keng C. T.; Tan S. Y.; Tan Y. J.; Chen Q.; Kurisawa M. Microstructured Dextran Hydrogels for Burst-Free Sustained Release of PEGylated Protein Drugs. Biomaterials 2015, 63, 146–157. 10.1016/j.biomaterials.2015.06.008. 26100344

[ref179] Moreira Teixeira L. S.; Leijten J. C. H.; Wennink J. W. H.; Chatterjea A. G.; Feijen J.; van Blitterswijk C. A.; Dijkstra P. J.; Karperien M. The Effect of Platelet Lysate Supplementation of a Dextran-Based Hydrogel on Cartilage Formation. Biomaterials 2012, 33 (14), 3651–3661. 10.1016/j.biomaterials.2012.01.051. 22349290

[ref180] Santos S. C. N. D. S.; Sigurjonsson Ó. E.; Custódio C. D. A.; Mano J. F. C. D. L. Blood Plasma Derivatives for Tissue Engineering and Regenerative Medicine Therapies. Tissue Eng., Part B 2018, 24 (6), 454–462. 10.1089/ten.teb.2018.0008. PMC6443031 29737237

[ref181] Portalska K. J.; Teixeira L. M.; Leijten J. C. H.; Jin R.; Van Blitterswijk C.; De Boer J.; Karperien M. Boosting Angiogenesis and Functional Vascularization in Injectable Dextran-Hyaluronic Acid Hydrogels by Endothelial-like Mesenchymal Stromal Cells. Tissue Eng., Part A 2014, 20 (3–4), 819–829. 10.1089/ten.tea.2013.0280. 24070233

[ref182] Fathi E.; Nassiri S. M.; Atyabi N.; Ahmadi S. H.; Imani M.; Farahzadi R.; Rabbani S.; Akhlaghpour S.; Sahebjam M.; Taheri M. Induction of Angiogenesis via Topical Delivery of Basic-Fibroblast Growth Factor from Polyvinyl Alcohol–Dextran Blend Hydrogel in an Ovine Model of Acute Myocardial Infarction. J. Tissue Eng. Regener. Med. 2013, 7, 697–707. 10.1002/term.1460. 22674791

[ref183] Sun G.; Shen Y. I.; Ho C. C.; Kusuma S.; Gerecht S. Functional Groups Affect Physical and Biological Properties of Dextran-Based Hydrogels. J. Biomed. Mater. Res., Part A 2009, 93A (3), 1080–1090. 10.1002/jbm.a.32604. 19753626

[ref184] Chen F. M.; Zhao Y. M.; Sun H. H.; Jin T.; Wang Q. T.; Zhou W.; Wu Z. F.; Jin Y. Novel Glycidyl Methacrylated Dextran (Dex-GMA)/Gelatin Hydrogel Scaffolds Containing Microspheres Loaded with Bone Morphogenetic Proteins: Formulation and Characteristics. J. Controlled Release 2007, 118 (1), 65–77. 10.1016/j.jconrel.2006.11.016. 17250921

[ref185] Pescosolido L.; Miatto S.; Di Meo C.; Cencetti C.; Coviello T.; Alhaique F.; Matricardi P. Injectable and in Situ Gelling Hydrogels for Modified Protein Release. Eur. Biophys. J. 2010, 39 (6), 903–909. 10.1007/s00249-009-0440-2. 19326113

[ref186] Sun G.; Shen Y.-I.; Kusuma S.; Fox-Talbot K.; Steenbergen C. J.; Gerecht S. Functional Neovascularization of Biodegradable Dextran Hydrogels with Multiple Angiogenic Growth Factors. Biomaterials 2011, 32 (1), 95–106. 10.1016/j.biomaterials.2010.08.091. 20870284

[ref187] Stahl P. J.; Chan T. R.; Shen Y.-I.; Sun G.; Gerecht S.; Yu M. Capillary Network-Like Organization of Endothelial Cells in PEGDA Scaffolds Encoded with Angiogenic Signals via Triple Helical Hybridization. Adv. Funct. Mater. 2014, 24 (21), 3213–3225. 10.1002/adfm.201303217. 25541582 PMC4273917

[ref188] Wu X.; He C.; Wu Y.; Chen X.; Cheng J. Nanogel-Incorporated Physical and Chemical Hybrid Gels for Highly Effective Chemo-Protein Combination Therapy. Adv. Funct. Mater. 2015, 25 (43), 6744–6755. 10.1002/adfm.201502742.

[ref189] Wang P.; Huang S.; Hu Z.; Yang W.; Lan Y.; Zhu J.; Hancharou A.; Guo R.; Tang B. In Situ Formed Anti-Inflammatory Hydrogel Loading Plasmid DNA Encoding VEGF for Burn Wound Healing. Acta Biomater. 2019, 100, 191–201. 10.1016/j.actbio.2019.10.004. 31586729

[ref190] Conde J.; Oliva N.; Atilano M.; Song H. S.; Artzi N. Self-Assembled RNA-Triple-Helix Hydrogel Scaffold for MicroRNA Modulation in the Tumour Microenvironment. Nat. Mater. 2016, 15 (3), 353–363. 10.1038/nmat4497. 26641016 PMC6594154

[ref191] Huang X.; Wu L.; Li X.; Lowe T. L. Thermoresponsive and Biodegradable Hydrogels for Sustained Release of Nerve Growth Factor to Stimulate Neurite Outgrowth. Macromol. Symp. 2012, 317–318 (1), 301–309. 10.1002/masy.201200022.

[ref192] Zhu H.; Li X.; Yuan M.; Wan W.; Hu M.; Wang X.; Jiang X. Intramyocardial Delivery of BFGF with a Biodegradable and Thermosensitive Hydrogel Improves Angiogenesis and Cardio-protection in Infarcted Myocardium. Exp. Ther. Med. 2017, 14 (4), 3609–3615. 10.3892/etm.2017.5015. 29042955 PMC5639332

[ref193] Hu C. H.; Zhang L.; Wu D. Q.; Cheng S. X.; Zhang X. Z.; Zhuo R. X. Heparin-Modified PEI Encapsulated in Thermosensitive Hydrogels for Efficient Gene Delivery and Expression. J. Mater. Chem. 2009, 19 (20), 3189–3197. 10.1039/b817956e.

[ref194] Wan W. G.; Jiang X. J.; Li X. Y.; Zhang C.; Yi X.; Ren S.; Zhang X. Z. Enhanced Cardioprotective Effects Mediated by Plasmid Containing the Short-Hairpin RNA of Angiotensin Converting Enzyme with a Biodegradable Hydrogel after Myocardial Infarction. J. Biomed. Mater. Res., Part A 2014, 102 (10), 3452–3458. 10.1002/jbm.a.35014. 24222385

[ref195] Ferreira L. S.; Gerecht S.; Fuller J.; Shieh H. F.; Vunjak-Novakovic G.; Langer R. Bioactive Hydrogel Scaffolds for Controllable Vascular Differentiation of Human Embryonic Stem Cells. Biomaterials 2007, 28 (17), 2706–2717. 10.1016/j.biomaterials.2007.01.021. 17346788 PMC1903348

[ref196] Keller S.; Teora S. P.; Hu G. X.; Nijemeisland M.; Wilson D. A. High-Throughput Design of Biocompatible Enzyme-Based Hydrogel Microparticles with Autonomous Movement. Angew. Chem., Int. Ed. 2018, 57 (31), 9814–9817. 10.1002/anie.201805661. 29917309

[ref197] Fujioka-Kobayashi M.; Ota M. S.; Shimoda A.; Nakahama K.; Akiyoshi K.; Miyamoto Y.; Iseki S. Cholesteryl Group- and Acryloyl Group-Bearing Pullulan Nanogel to Deliver BMP2 and FGF18 for Bone Tissue Engineering. Biomaterials 2012, 33 (30), 7613–7620. 10.1016/j.biomaterials.2012.06.075. 22800537

[ref198] Cutiongco M. F. A.; Teo B. K. K.; Yim E. K. F. Composite Scaffolds of Interfacial Polyelectrolyte Fibers for Temporally Controlled Release of Biomolecules. J. Visualized Exp. 2015, 2015 (102), e53079 10.3791/53079. PMC4692548 26325384

[ref199] Sekine Y.; Moritani Y.; Ikeda-Fukazawa T.; Sasaki Y.; Akiyoshi K. A Hybrid Hydrogel Biomaterial by Nanogel Engineering: Bottom-up Design with Nanogel and Liposome Building Blocks to Develop a Multidrug Delivery System. Adv. Healthcare Mater. 2012, 1 (6), 722–728. 10.1002/adhm.201200175. 23184823

[ref200] King T. W.; Patrick C. W. Development Andin Vitro Characterization of Vascular Endothelial Growth Factor (VEGF)-Loaded Poly(DL-Lactic-Co-Glycolic Acid)/Poly(Ethylene Glycol) Microspheres Using a Solid Encapsulation/Single Emulsion/Solvent Extraction Technique. J. Biomed. Mater. Res. 2000, 51 (3), 383–390. 10.1002/1097-4636(20000905)51:3<383::AID-JBM12>3.0.CO;2-D. 10880080

[ref201] Rui J.; Dadsetan M.; Runge M. B.; Spinner R. J.; Yaszemski M. J.; Windebank A. J.; Wang H. Controlled Release of Vascular Endothelial Growth Factor Using Poly-Lactic-Co-Glycolic Acid Microspheres: In Vitro Characterization and Application in Polycaprolactone Fumarate Nerve Conduits. Acta Biomater. 2012, 8 (2), 511–518. 10.1016/j.actbio.2011.10.001. 22019759 PMC3972821

[ref202] Moradali M. F.; Rehm B. H. A. Bacterial Biopolymers: From Pathogenesis to Advanced Materials. Nat. Rev. Microbiol. 2020, 18 (4), 195–210. 10.1038/s41579-019-0313-3. 31992873 PMC7223192

[ref203] Fraser J. R. E.; Laurent T. C.; Laurent U. Hyaluronan: Its Nature, Distribution, Functions and Turnover. J. Intern. Med. 1997, 242, 27–33. 10.1046/j.1365-2796.1997.00170.x. 9260563

[ref204] Trombino S.; Servidio C.; Curcio F.; Cassano R. Strategies for Hyaluronic Acid-Based Hydrogel Design in Drug Delivery. Pharmaceutics 2019, 11 (8), 407 10.3390/pharmaceutics11080407. PMC6722772 31408954

[ref205] Voelcker V.; Gebhardt C.; Averbeck M.; Saalbach A.; Wolf V.; Weih F.; Sleeman J.; Anderegg U.; Simon J. Hyaluronan Fragments Induce Cytokine and Metalloprotease Upregulation in Human Melanoma Cells in Part by Signalling via TLR4. Exp. Dermatol. 2008, 17 (2), 100–107. 10.1111/j.1600-0625.2007.00638.x. 18031543

[ref206] Gao F.; Liu Y.; He Y.; Yang C.; Wang Y.; Shi X.; et al. Hyaluronan Oligosaccharides Promote Excisional Wound Healing through Enhanced Angiogenesis. Matrix Biol. 2010, 29, 107–116. 10.1016/j.matbio.2009.11.002. 19913615

[ref207] Li L.; Jiang G.; Yu W.; Liu D.; Chen H.; Liu Y.; Huang Q.; Tong Z.; Yao J.; Kong X. A Composite Hydrogel System Containing Glucose-Responsive Nanocarriers for Oral Delivery of Insulin. Mater. Sci. Eng., C 2016, 69, 37–45. 10.1016/j.msec.2016.06.059. 27612686

[ref208] Agrawal N. K.; Allen P.; Song Y. H.; Wachs R. A.; Du Y.; Ellington A. D.; Schmidt C. E. Oligonucleotide-Functionalized Hydrogels for Sustained Release of Small Molecule (Aptamer) Therapeutics. Acta Biomater. 2020, 102, 315–325. 10.1016/j.actbio.2019.11.037. 31760222

[ref209] Babo P. S.; Pires R. L.; Santos L.; Franco A.; Rodrigues F.; Leonor I.; Reis R. L.; Gomes M. E. Platelet Lysate-Loaded Photocrosslinkable Hyaluronic Acid Hydrogels for Periodontal Endogenous Regenerative Technology. ACS Biomater. Sci. Eng. 2017, 3 (7), 1359–1369. 10.1021/acsbiomaterials.6b00508. 33429694

[ref210] Deng Y.; Sun A. X.; Overholt K. J.; Yu G. Z.; Fritch M. R.; Alexander P. G.; Shen H.; Tuan R. S.; Lin H. Enhancing Chondrogenesis and Mechanical Strength Retention in Physiologically Relevant Hydrogels with Incorporation of Hyaluronic Acid and Direct Loading of TGF-β. Acta Biomater. 2019, 83, 167–176. 10.1016/j.actbio.2018.11.022. 30458242 PMC6733255

[ref211] Thönes S.; Rother S.; Wippold T.; Blaszkiewicz J.; Balamurugan K.; Moeller S.; Ruiz-Gómez G.; Schnabelrauch M.; Scharnweber D.; Saalbach A.; Rademann J.; Pisabarro M. T.; Hintze V.; Anderegg U. Hyaluronan/Collagen Hydrogels Containing Sulfated Hyaluronan Improve Wound Healing by Sustained Release of Heparin-Binding EGF-like Growth Factor. Acta Biomater. 2019, 86, 135–147. 10.1016/j.actbio.2019.01.029. 30660005

[ref212] Patterson J.; Siew R.; Herring S. W.; Lin A. S. P.; Guldberg R.; Stayton P. S. Hyaluronic Acid Hydrogels with Controlled Degradation Properties for Oriented Bone Regeneration. Biomaterials 2010, 31 (26), 6772–6781. 10.1016/j.biomaterials.2010.05.047. 20573393 PMC2907529

[ref213] Bae M. S.; Ohe J. Y.; Lee J. B.; Heo D. N.; Byun W.; Bae H.; Kwon Y. D.; Kwon I. K. Photo-Cured Hyaluronic Acid-Based Hydrogels Containing Growth and Differentiation Factor 5 (GDF-5) for Bone Tissue Regeneration. Bone 2014, 59, 189–198. 10.1016/j.bone.2013.11.019. 24291420

[ref214] Eckhouse S. R.; Purcell B. P.; McGarvey J. R.; Lobb D.; Logdon C. B.; Doviak H.; O’Neill J. W.; Shuman J. A.; Novack C. P.; Zellars K. N.; Pettaway S.; Black R. A.; Khakoo A.; Lee T. W.; Mukherjee R.; Gorman J. H.; Gorman R. C.; Burdick J. A.; Spinale F. G. Local Hydrogel Release of Recombinant TIMP-3 Attenuates Adverse Left Ventricular Remodeling after Experimental Myocardial Infarction. Sci. Transl. Med. 2014, 6 (223), 223ra21 10.1126/scitranslmed.3007244. PMC4365799 24523321

[ref215] Luo C.; Xu G.; Wang X.; Tu M.; Zeng R.; Rong J.; Zhao J. Self-Reinforcement and Protein Sustained Delivery of Hyaluronan Hydrogel by Tailoring a Dually Cross-Linked Network. Mater. Sci. Eng., C 2015, 46, 316–324. 10.1016/j.msec.2014.10.066. 25491993

[ref216] Jha A. K.; Mathur A.; Svedlund F. L.; Ye J.; Yeghiazarians Y.; Healy K. E. Molecular Weight and Concentration of Heparin in Hyaluronic Acid-Based Matrices Modulates Growth Factor Retention Kinetics and Stem Cell Fate. J. Controlled Release 2015, 209, 308–316. 10.1016/j.jconrel.2015.04.034. PMC4465536 25931306

[ref217] Lee S. Y.; Park Y.; Hwang S. J. Effect of BFGF and Fibroblasts Combined with Hyaluronic Acid-Based Hydrogels on Soft Tissue Augmentation: An Experimental Study in Rats. Maxillofac. Plast. Reconstr. Surg. 2019, 41, 47 10.1186/s40902-019-0234-0. 31750275 PMC6834819

[ref218] Holloway J. L.; Ma H.; Rai R.; Hankenson K. D.; Burdick J. A. Synergistic Effects of SDF-1α and BMP-2 Delivery from Proteolytically Degradable Hyaluronic Acid Hydrogels for Bone Repair. Macromol. Biosci. 2015, 15 (9), 1218–1223. 10.1002/mabi.201500178. 26059079 PMC4558375

[ref219] Villate-Beitia I.; Truong N. F.; Gallego I.; Zárate J.; Puras G.; Pedraz J. L.; Segura T. Hyaluronic Acid Hydrogel Scaffolds Loaded with Cationic Niosomes for Efficient Non-Viral Gene Delivery. RSC Adv. 2018, 8 (56), 31934–31942. 10.1039/C8RA05125A. 30294422 PMC6146377

[ref220] Gojgini S.; Tokatlian T.; Segura T. Utilizing Cell-Matrix Interactions to Modulate Gene Transfer to Stem Cells inside Hyaluronic Acid Hydrogels. Mol. Pharmaceutics 2011, 8 (5), 1582–1591. 10.1021/mp200171d. PMC4104282 21823632

[ref221] Truong N. F.; Segura T. Sustained Transgene Expression via Hydrogel-Mediated Gene Transfer Results from Multiple Transfection Events. ACS Biomater. Sci. Eng. 2018, 4 (3), 981–987. 10.1021/acsbiomaterials.7b00957. 33418780

[ref222] Tokatlian T.; Cam C.; Segura T. Porous Hyaluronic Acid Hydrogels for Localized Nonviral DNA Delivery in a Diabetic Wound Healing Model. Adv. Healthcare Mater. 2015, 4 (7), 1084–1091. 10.1002/adhm.201400783. PMC4433401 25694196

[ref223] Bian L.; Zhai D. Y.; Tous E.; Rai R.; Mauck R. L.; Burdick J. A. Enhanced MSC Chondrogenesis Following Delivery of TGF-B3 from Alginate Microspheres within Hyaluronic Acid Hydrogels in Vitro and in Vivo. Biomaterials 2011, 32 (27), 6425–6434. 10.1016/j.biomaterials.2011.05.033. 21652067 PMC3134110

[ref224] Ekaputra A. K.; Prestwich G. D.; Cool S. M.; Hutmacher D. W. The Three-Dimensional Vascularization of Growth Factor-Releasing Hybrid Scaffold of Poly (E{open}-Caprolactone)/Collagen Fibers and Hyaluronic Acid Hydrogel. Biomaterials 2011, 32 (32), 8108–8117. 10.1016/j.biomaterials.2011.07.022. 21807407

[ref225] Srinivasan P. P.; McCoy S. Y.; Jha A. K.; Yang W.; Jia X.; Farach-Carson M. C.; Kirn-Safran C. B. Injectable Perlecan Domain 1-Hyaluronan Microgels Potentiate the Cartilage Repair Effect of BMP2 in a Murine Model of Early Osteoarthritis. Biomed. Mater. 2012, 7 (2), 024109 10.1088/1748-6041/7/2/024109. 22455987 PMC3367563

[ref226] Hulsart-Billström G.; Yuen P. K.; Marsell R.; Hilborn J.; Larsson S.; Ossipov D. Bisphosphonate-Linked Hyaluronic Acid Hydrogel Sequesters and Enzymatically Releases Active Bone Morphogenetic Protein-2 for Induction of Osteogenic Differentiation. Biomacromolecules 2013, 14 (9), 3055–3063. 10.1021/bm400639e. 23947433

[ref227] Seelbach R. J.; Fransen P.; Pulido D.; D’Este M.; Duttenhoefer F.; Sauerbier S.; Freiman T. M.; Niemeyer P.; Albericio F.; Alini M.; Royo M.; Mata A.; Eglin D. Injectable Hyaluronan Hydrogels with Peptide-Binding Dendrimers Modulate the Controlled Release of BMP-2 and TGF-B1. Macromol. Biosci. 2015, 15 (8), 1035–1044. 10.1002/mabi.201500082. 25943094

[ref228] Liu X.; Yang Y.; Niu X.; Lin Q.; Zhao B.; Wang Y.; Zhu L. An in Situ Photocrosslinkable Platelet Rich Plasma – Complexed Hydrogel Glue with Growth Factor Controlled Release Ability to Promote Cartilage Defect Repair. Acta Biomater. 2017, 62, 179–187. 10.1016/j.actbio.2017.05.023. 28501713

[ref229] Silva C. R.; Babo P. S.; Gulino M.; Costa L.; Oliveira J. M.; Silva-Correia J.; Domingues R. M. A.; Reis R. L.; Gomes M. E. Injectable and Tunable Hyaluronic Acid Hydrogels Releasing Chemotactic and Angiogenic Growth Factors for Endodontic Regeneration. Acta Biomater. 2018, 77, 155–171. 10.1016/j.actbio.2018.07.035. 30031163

[ref230] Rother S.; Krönert V.; Hauck N.; Berg A.; Moeller S.; Schnabelrauch M.; Thiele J.; Scharnweber D.; Hintze V. Hyaluronan/Collagen Hydrogel Matrices Containing High-Sulfated Hyaluronan Microgels for Regulating Transforming Growth Factor-B1. J. Mater. Sci.: Mater. Med. 2019, 30 (6), 1–5. 10.1007/s10856-019-6267-1. 31127393

[ref231] Grab A. L.; Seckinger A.; Horn P.; Hose D.; Cavalcanti-Adam E. A. Hyaluronan Hydrogels Delivering BMP-6 for Local Targeting of Malignant Plasma Cells and Osteogenic Differentiation of Mesenchymal Stromal Cells. Acta Biomater. 2019, 96, 258–270. 10.1016/j.actbio.2019.07.018. 31302300

[ref232] Todeschi M. R.; El Backly R. M.; Varghese O. P.; Hilborn J.; Cancedda R.; Mastrogiacomo M. Host Cell Recruitment Patterns by Bone Morphogenetic Protein-2 Releasing Hyaluronic Acid Hydrogels in a Mouse Subcutaneous Environment. Regener. Med. 2017, 12 (5), 525–539. 10.2217/rme-2017-0023. 28770657

[ref233] Kumar P.; Ciftci S.; Barthes J.; Knopf-Marques H.; Muller C. B.; Debry C.; Vrana N. E.; Ghaemmaghami A. M. A Composite Gelatin/Hyaluronic Acid Hydrogel as an ECM Mimic for Developing Mesenchymal Stem Cell-Derived Epithelial Tissue Patches. J. Tissue Eng. Regener. Med. 2020, 14 (1), 45–57. 10.1002/term.2962. 31597222

[ref234] Bhakta G.; Rai B.; Lim Z. X. H.; Hui J. H.; Stein G. S.; van Wijnen A. J.; Nurcombe V.; Prestwich G. D.; Cool S. M. Hyaluronic Acid-Based Hydrogels Functionalized with Heparin That Support Controlled Release of Bioactive BMP-2. Biomaterials 2012, 33 (26), 6113–6122. 10.1016/j.biomaterials.2012.05.030. 22687758 PMC3628623

[ref235] Shamskhou E. A.; Kratochvil M. J.; Orcholski M. E.; Nagy N.; Kaber G.; Steen E.; Balaji S.; Yuan K.; Keswani S.; Danielson B.; Gao M.; Medina C.; Nathan A.; Chakraborty A.; Bollyky P. L.; De Jesus Perez V. A. Hydrogel-Based Delivery of Il-10 Improves Treatment of Bleomycin-Induced Lung Fibrosis in Mice. Biomaterials 2019, 203, 52–62. 10.1016/j.biomaterials.2019.02.017. 30852423 PMC6430662

[ref236] Liu G.; Wu R.; Yang B.; Shi Y.; Deng C.; Atala A.; Mou S.; Criswell T.; Zhang Y. A Cocktail of Growth Factors Released from a Heparin Hyaluronic-Acid Hydrogel Promotes the Myogenic Potential of Human Urine-Derived Stem Cells in Vivo. Acta Biomater. 2020, 107, 50–64. 10.1016/j.actbio.2020.02.005. 32044457 PMC8176383

[ref237] Wang W.; Tan B.; Chen J.; Bao R.; Zhang X.; Liang S.; Shang Y.; Liang W.; Cui Y.; Fan G.; Jia H.; Liu W. An Injectable Conductive Hydrogel Encapsulating Plasmid DNA-ENOs and ADSCs for Treating Myocardial Infarction. Biomaterials 2018, 160, 69–81. 10.1016/j.biomaterials.2018.01.021. 29396380

[ref238] Yang J. A.; Kim H.; Park K.; Hahn S. K. Molecular Design of Hyaluronic Acid Hydrogel Networks for Long-Term Controlled Delivery of Human Growth Hormone. Soft Matter 2011, 7 (3), 868–870. 10.1039/c0sm01011a.

[ref239] Ma X.; Xu T.; Chen W.; Qin H.; Chi B.; Ye Z. Injectable Hydrogels Based on the Hyaluronic Acid and Poly (γ-Glutamic Acid) for Controlled Protein Delivery. Carbohydr. Polym. 2018, 179 (30), 100–109. 10.1016/j.carbpol.2017.09.071. 29111032

[ref240] Paidikondala M.; Nawale G. N.; Varghese O. P. Insights into SiRNA Transfection in Suspension: Efficient Gene Silencing in Human Mesenchymal Stem Cells Encapsulated in Hyaluronic Acid Hydrogel. Biomacromolecules 2019, 20 (3), 1317–1324. 10.1021/acs.biomac.8b01712. 30642167

[ref241] Varghese O. P.; Kisiel M.; Martinez-Sanz E.; Ossipov D. A.; Hilborn J. Synthesis of Guanidinium-Modified Hyaluronic Acid Hydrogel. Macromol. Rapid Commun. 2010, 31 (13), 1175–1180. 10.1002/marc.200900906. 21590872

[ref242] Yan H. J.; Casalini T.; Hulsart-Billström G.; Wang S.; Oommen O. P.; Salvalaglio M.; Larsson S.; Hilborn J.; Varghese O. P. Synthetic Design of Growth Factor Sequestering Extracellular Matrix Mimetic Hydrogel for Promoting in Vivo Bone Formation. Biomaterials 2018, 161, 190–202. 10.1016/j.biomaterials.2018.01.041. 29421555

[ref243] Wang L. L.; Chung J. J.; Li E. C.; Uman S.; Atluri P.; Burdick J. A. Injectable and Protease-Degradable Hydrogel for SiRNA Sequestration and Triggered Delivery to the Heart. J. Controlled Release 2018, 285 (July), 152–161. 10.1016/j.jconrel.2018.07.004. PMC6134398 29981357

[ref244] Levinson C.; Lee M.; Applegate L. A.; Zenobi-Wong M. An Injectable Heparin-Conjugated Hyaluronan Scaffold for Local Delivery of Transforming Growth Factor B1 Promotes Successful Chondrogenesis. Acta Biomater. 2019, 99, 168–180. 10.1016/j.actbio.2019.09.017. 31536840

[ref245] Jooybar E.; Abdekhodaie M. J.; Karperien M.; Mousavi A.; Alvi M.; Dijkstra P. J. Developing Hyaluronic Acid Microgels for Sustained Delivery of Platelet Lysate for Tissue Engineering Applications. Int. J. Biol. Macromol. 2020, 144, 837–846. 10.1016/j.ijbiomac.2019.10.036. 31715235

[ref246] O’Dwyer J.; Murphy R.; Dolan E. B.; Kovarova L.; Pravda M.; Velebny V.; Heise A.; Duffy G. P.; Cryan S. A. Development of a Nanomedicine-Loaded Hydrogel for Sustained Delivery of an Angiogenic Growth Factor to the Ischaemic Myocardium. Drug Delivery Transl. Res. 2020, 10 (2), 440–454. 10.1007/s13346-019-00684-5. 31691161

[ref247] Vainieri M. L.; Lolli A.; Kops N.; D’Atri D.; Eglin D.; Yayon A.; Alini M.; Grad S.; Sivasubramaniyan K.; van Osch G. J. V. M. Evaluation of Biomimetic Hyaluronic-Based Hydrogels with Enhanced Endogenous Cell Recruitment and Cartilage Matrix Formation. Acta Biomater. 2020, 101, 293–303. 10.1016/j.actbio.2019.11.015. 31726249

[ref248] Jooybar E.; Abdekhodaie M. J.; Alvi M.; Mousavi A.; Karperien M.; Dijkstra P. J. An Injectable Platelet Lysate-Hyaluronic Acid Hydrogel Supports Cellular Activities and Induces Chondrogenesis of Encapsulated Mesenchymal Stem Cells. Acta Biomater. 2019, 83, 233–244. 10.1016/j.actbio.2018.10.031. 30366137

[ref249] Egbu R.; Brocchini S.; Khaw P. T.; Awwad S. Antibody Loaded Collapsible Hyaluronic Acid Hydrogels for Intraocular Delivery. Eur. J. Pharm. Biopharm. 2018, 124, 95–103. 10.1016/j.ejpb.2017.12.019. 29294367

[ref250] Lee F.; Chung J. E.; Xu K.; Kurisawa M. Injectable Degradation-Resistant Hyaluronic Acid Hydrogels Cross-Linked via the Oxidative Coupling of Green Tea Catechin. ACS Macro Lett. 2015, 4 (9), 957–960. 10.1021/acsmacrolett.5b00544. 35596463

[ref251] Shin M.; Lee H. Gallol-Rich Hyaluronic Acid Hydrogels: Shear-Thinning, Protein Accumulation against Concentration Gradients, and Degradation-Resistant Properties. Chem. Mater. 2017, 29 (19), 8211–8220. 10.1021/acs.chemmater.7b02267.

[ref252] Hsieh H. Y.; Lin W. Y.; Lee A. L.; Li Y. C.; Chen Y. J.; Chen K. C.; Young T. H. Hyaluronic Acid on the Urokinase Sustained Release with a Hydrogel System Composed of Poloxamer 407: HA/P407 Hydrogel System for Drug Delivery. PLoS One 2020, 15 (3), e0227784 10.1371/journal.pone.0227784. 32160196 PMC7065803

[ref253] Ansari S.; Diniz I. M.; Chen C.; Aghaloo T.; Wu B. M.; Shi S.; Moshaverinia A. Alginate/Hyaluronic Acid Hydrogel Delivery System Characteristics Regulate the Differentiation of Periodontal Ligament Stem Cells toward Chondrogenic Lineage. J. Mater. Sci.: Mater. Med. 2017, 28 (10), 162 10.1007/s10856-017-5974-8. 28914392

[ref254] Tsaryk R.; Gloria A.; Russo T.; Anspach L.; De Santis R.; Ghanaati S.; Unger R. E.; Ambrosio L.; Kirkpatrick C. J. Collagen-Low Molecular Weight Hyaluronic Acid Semi-Interpenetrating Network Loaded with Gelatin Microspheres for Cell and Growth Factor Delivery for Nucleus Pulposus Regeneration. Acta Biomater. 2015, 20, 10–21. 10.1016/j.actbio.2015.03.041. 25861947

[ref255] Cooke M. J.; Wang Y.; Morshead C. M.; Shoichet M. S. Controlled Epi-Cortical Delivery of Epidermal Growth Factor for the Stimulation of Endogenous Neural Stem Cell Proliferation in Stroke-Injured Brain. Biomaterials 2011, 32 (24), 5688–5697. 10.1016/j.biomaterials.2011.04.032. 21550655

[ref256] He Z.; Zang H.; Zhu L.; Huang K.; Yi T.; Zhang S.; Cheng S. An Anti-Inflammatory Peptide and Brain-Derived Neurotrophic Factor-Modified Hyaluronan-Methylcellulose Hydrogel Promotes Nerve Regeneration in Rats with Spinal Cord Injury. Int. J. Nanomed. 2019, 14, 721–732. 10.2147/IJN.S187854. PMC6342221 30705588

[ref257] Parker J.; Mitrousis N.; Shoichet M. S. Hydrogel for Simultaneous Tunable Growth Factor Delivery and Enhanced Viability of Encapsulated Cells in Vitro. Biomacromolecules 2016, 17 (2), 476–484. 10.1021/acs.biomac.5b01366. 26762290

[ref258] Vulic K.; Shoichet M. S. Tunable Growth Factor Delivery from Injectable Hydrogels for Tissue Engineering. J. Am. Chem. Soc. 2012, 134 (2), 882–885. 10.1021/ja210638x. 22201513 PMC3260740

[ref259] Delplace V.; Ortin-Martinez A.; Tsai E. L. S.; Amin A. N.; Wallace V.; Shoichet M. S. Controlled Release Strategy Designed for Intravitreal Protein Delivery to the Retina. J. Controlled Release 2019, 293, 10–20. 10.1016/j.jconrel.2018.11.012. 30419267

[ref260] Khaing Z. Z.; Agrawal N. K.; Park J. H.; Xin S.; Plumton G. C.; Lee K. H.; Huang Y. J.; Niemerski A. L.; Schmidt C. E.; Grau J. W. Localized and Sustained Release of Brain-Derived Neurotrophic Factor from Injectable Hydrogel/Microparticle Composites Fosters Spinal Learning after Spinal Cord Injury. J. Mater. Chem. B 2016, 4 (47), 7560–7571. 10.1039/C6TB01602B. 32263813

[ref261] Obermeyer J. M.; Tuladhar A.; Payne S. L.; Ho E.; Morshead C. M.; Shoichet M. S. Local Delivery of Brain-Derived Neurotrophic Factor Enables Behavioral Recovery and Tissue Repair in Stroke-Injured Rats. Tissue Eng., Part A 2019, 25 (15–16), 1175–1187. 10.1089/ten.tea.2018.0215. 30612516

[ref262] Pereira C. L.; Gonçalves R. M.; Peroglio M.; Pattappa G.; D’Este M.; Eglin D.; Barbosa M. A.; Alini M.; Grad S. The Effect of Hyaluronan-Based Delivery of Stromal Cell-Derived Factor-1 on the Recruitment of MSCs in Degenerating Intervertebral Discs. Biomaterials 2014, 35 (28), 8144–8153. 10.1016/j.biomaterials.2014.06.017. 24969636

[ref263] Peroglio M.; Eglin D.; Benneker L. M.; Alini M.; Grad S. Thermoreversible Hyaluronan-Based Hydrogel Supports in Vitro and Ex Vivo Disc-like Differentiation of Human Mesenchymal Stem Cells. Spine J. 2013, 13 (11), 1627–1639. 10.1016/j.spinee.2013.05.029. 23830827

[ref264] Fahmy-Garcia S.; Mumcuoglu D.; de Miguel L.; Dieleman V.; Witte-Bouma J.; van der Eerden B. C. J.; van Driel M.; Eglin D.; Verhaar J. A. N.; Kluijtmans S. G. J. M.; van Osch G. J. V. M.; Farrell E. Novel In Situ Gelling Hydrogels Loaded with Recombinant Collagen Peptide Microspheres as a Slow-Release System Induce Ectopic Bone Formation. Adv. Healthcare Mater. 2018, 7 (21), 1800507 10.1002/adhm.201800507. 30230271

[ref265] Awwad S.; Abubakre A.; Angkawinitwong U.; Khaw P. T.; Brocchini S. In Situ Antibody-Loaded Hydrogel for Intravitreal Delivery. Eur. J. Pharm. Sci. 2019, 137 (July), 104993 10.1016/j.ejps.2019.104993. 31302214

[ref266] Steele A. N.; Paulsen M. J.; Wang H.; Stapleton L. M.; Lucian H. J.; Eskandari A.; Hironaka C. E.; Farry J. M.; Baker S. W.; Thakore A. D.; Jaatinen K. J.; Tada Y.; Hollander M. J.; Williams K. M.; Seymour A. J.; Totherow K. P.; Yu A. C.; Cochran J. R.; Appel E. A.; Woo Y. J. Multi-Phase Catheter-Injectable Hydrogel Enables Dual-Stage Protein-Engineered Cytokine Release to Mitigate Adverse Left Ventricular Remodeling Following Myocardial Infarction in a Small Animal Model and a Large Animal Model. Cytokine+ 2020, 127, 154974 10.1016/j.cyto.2019.154974. 31978642

[ref267] Wei K.; Zhu M.; Sun Y.; Xu J.; Feng Q.; Lin S.; Wu T.; Xu J.; Tian F.; Xia J.; Li G.; Bian L. Robust Biopolymeric Supramolecular “Host-Guest Macromer” Hydrogels Reinforced by in Situ Formed Multivalent Nanoclusters for Cartilage Regeneration. Macromolecules 2016, 49 (3), 866–875. 10.1021/acs.macromol.5b02527.

[ref268] Rodell C. B.; Rai R.; Faubel S.; Burdick J. A.; Soranno D. E. Local Immunotherapy via Delivery of Interleukin-10 and Transforming Growth Factor β Antagonist for Treatment of Chronic Kidney Disease. J. Controlled Release 2015, 206, 131–139. 10.1016/j.jconrel.2015.03.025. 25804871

[ref269] Chen Y.-R.; Zhou Z.-X.; Zhang J.-Y.; Yuan F.-Z.; Xu B.-B.; Guan J.; Han C.; Jiang D.; Yang Y.-Y.; Yu J.-K. Low-Molecular-Weight Heparin-Functionalized Chitosan-Chondroitin Sulfate Hydrogels for Controlled Release of TGF-B3 and in Vitro Neocartilage Formation. Front. Chem. 2019, 7, 745 10.3389/fchem.2019.00745. 31737612 PMC6839338

[ref270] Conovaloff A. W.; Beier B. L.; Irazoqui P. P.; Panitch A. Effects of a Synthetic Bioactive Peptide on Neurite Growth and Nerve Growth Factor Release in Chondroitin Sulfate Hydrogels. Biomatter 2011, 1 (2), 165–173. 10.4161/biom.17849. 23507745 PMC3549887

[ref271] Butterfield K. C.; Conovaloff A. W.; Panitch A. Development of Affinity-Based Delivery of NGF from a Chondroitin Sulfate Biomaterial. Biomatter 2011, 1 (2), 174–181. 10.4161/biom.18791. 23507746 PMC3549888

[ref272] Wang S.; Oommen O. P.; Yan H.; Varghese O. P. Mild and Efficient Strategy for Site-Selective Aldehyde Modification of Glycosaminoglycans: Tailoring Hydrogels with Tunable Release of Growth Factor. Biomacromolecules 2013, 14 (7), 2427–2432. 10.1021/bm400612h. 23721079

[ref273] Du M.; Liang H.; Mou C.; Li X.; Sun J.; Zhuang Y.; Xiao Z.; Chen B.; Dai J. Regulation of Human Mesenchymal Stem Cells Differentiation into Chondrocytes in Extracellular Matrix-Based Hydrogel Scaffolds. Colloids Surf., B 2014, 114, 316–323. 10.1016/j.colsurfb.2013.10.001. 24231133

[ref274] Anjum F.; Lienemann P. S.; Metzger S.; Biernaskie J.; Kallos M. S.; Ehrbar M. Enzyme Responsive GAG-Based Natural-Synthetic Hybrid Hydrogel for Tunable Growth Factor Delivery and Stem Cell Differentiation. Biomaterials 2016, 87, 104–117. 10.1016/j.biomaterials.2016.01.050. 26914701

[ref275] Lim J. J.; Temenoff J. S. The Effect of Desulfation of Chondroitin Sulfate on Interactions with Positively Charged Growth Factors and Upregulation of Cartilaginous Markers in Encapsulated MSCs. Biomaterials 2013, 34 (21), 5007–5018. 10.1016/j.biomaterials.2013.03.037. 23570717 PMC3671883

[ref276] Schuurmans C. C. L.; Abbadessa A.; Bengtson M. A.; Pletikapic G.; Eral H. B.; Koenderink G.; Masereeuw R.; Hennink W. E.; Vermonden T. Complex Coacervation-Based Loading and Tunable Release of a Cationic Protein from Monodisperse Glycosaminoglycan Microgels. Soft Matter 2018, 14 (30), 6327–6341. 10.1039/C8SM00686E. 30024582

[ref277] Carulli D.; Laabs T.; Geller H. M.; Fawcett J. W. Chondroitin Sulfate Proteoglycans in Neural Development and Regeneration. Curr. Opin. Neurobiol. 2005, 15 (1), 116–120. 10.1016/j.conb.2005.01.014. 15721753

[ref278] Rabenstein D. L. Heparin and Heparan Sulfate: Structure and Function. Nat. Prod. Rep. 2002, 19 (3), 312–331. 10.1039/b100916h. 12137280

[ref279] Liang Y.; Kiick K. L. Heparin-Functionalized Polymeric Biomaterials in Tissue Engineering and Drug Delivery Applications. Acta Biomater. 2014, 10 (4), 1588–1600. 10.1016/j.actbio.2013.07.031. 23911941 PMC3937301

[ref280] Jeon O.; Powell C.; Solorio L. D.; Krebs M. D.; Alsberg E. Affinity-Based Growth Factor Delivery Using Biodegradable, Photocrosslinked Heparin-Alginate Hydrogels. J. Controlled Release 2011, 154 (3), 258–266. 10.1016/j.jconrel.2011.06.027. PMC3541683 21745508

[ref281] Tsurkan M. V.; Hauser P. V.; Zieris A.; Carvalhosa R.; Bussolati B.; Freudenberg U.; Camussi G.; Werner C. Growth Factor Delivery from Hydrogel Particle Aggregates to Promote Tubular Regeneration after Acute Kidney Injury. J. Controlled Release 2013, 167 (3), 248–255. 10.1016/j.jconrel.2013.01.030. 23395667

[ref282] Li Z.; Qu T.; Ding C.; Ma C.; Sun H.; Li S.; Liu X. Injectable Gelatin Derivative Hydrogels with Sustained Vascular Endothelial Growth Factor Release for Induced Angiogenesis. Acta Biomater. 2015, 13, 88–100. 10.1016/j.actbio.2014.11.002. 25462840 PMC4293253

[ref283] Zhao Y.-Z.; Jiang X.; Xiao J.; Lin Q.; Yu W.-Z.; Tian F.-R.; Mao K.-L.; Yang W.; Wong H. L.; Lu C.-T. Using NGF Heparin-Poloxamer Thermosensitive Hydrogels to Enhance the Nerve Regeneration for Spinal Cord Injury. Acta Biomater. 2016, 29, 71–80. 10.1016/j.actbio.2015.10.014. 26472614 PMC7517710

[ref284] Krieger J. R.; Ogle M. E.; McFaline-Figueroa J.; Segar C. E.; Temenoff J. S.; Botchwey E. A. Spatially Localized Recruitment of Anti-Inflammatory Monocytes by SDF-1α-Releasing Hydrogels Enhances Microvascular Network Remodeling. Biomaterials 2016, 77, 280–290. 10.1016/j.biomaterials.2015.10.045. 26613543 PMC4698334

[ref285] Kim H.; Park H.; Lee J. W.; Lee K. Y. Magnetic Field-Responsive Release of Transforming Growth Factor Beta 1 from Heparin-Modified Alginate Ferrogels. Carbohydr. Polym. 2016, 151, 467–473. 10.1016/j.carbpol.2016.05.090. 27474590

[ref286] Ding X.; Gao J.; Wang Z.; Awada H.; Wang Y. A Shear-Thinning Hydrogel That Extends in Vivo Bioactivity of FGF2. Biomaterials 2016, 111, 80–89. 10.1016/j.biomaterials.2016.09.026. 27728816

[ref287] Roberts J. J.; Farrugia B. L.; Green R. A.; Rnjak-Kovacina J.; Martens P. J. In Situ Formation of Poly(Vinyl Alcohol)-Heparin Hydrogels for Mild Encapsulation and Prolonged Release of Basic Fibroblast Growth Factor and Vascular Endothelial Growth Factor. J. Tissue Eng. 2016, 10.1177/2041731416677132. PMC5117248 27895888

[ref288] Xu H. L.; Xu J.; Shen B. X.; Zhang S. S.; Jin B. H.; Zhu Q. Y.; ZhuGe D. L.; Wu X. Q.; Xiao J.; Zhao Y. Z. Dual Regulations of Thermosensitive Heparin-Poloxamer Hydrogel Using ϵ-Polylysine: Bioadhesivity and Controlled KGF Release for Enhancing Wound Healing of Endometrial Injury. ACS Appl. Mater. Interfaces 2017, 9 (35), 29580–29594. 10.1021/acsami.7b10211. 28809108

[ref289] Kim I.; Lee S. S.; Bae S.; Lee H.; Hwang N. S. Heparin Functionalized Injectable Cryogel with Rapid Shape-Recovery Property for Neovascularization. Biomacromolecules 2018, 19 (6), 2257–2269. 10.1021/acs.biomac.8b00331. 29689163

[ref290] Claaßen C.; Southan A.; Grübel J.; Tovar G. E. M.; Borchers K. Interactions of Methacryloylated Gelatin and Heparin Modulate Physico-Chemical Properties of Hydrogels and Release of Vascular Endothelial Growth Factor. Biomed. Mater. 2018, 13 (5), 055008 10.1088/1748-605X/aacdb2. 29923498

[ref291] Schirmer L.; Chwalek K.; Tsurkan M. V.; Freudenberg U.; Werner C. Glycosaminoglycan-Based Hydrogels with Programmable Host Reactions. Biomaterials 2020, 228, 119557 10.1016/j.biomaterials.2019.119557. 31678844

[ref292] Wang P.; Berry D.; Moran A.; He F.; Tam T.; Chen L.; Chen S. Controlled Growth Factor Release in 3D-Printed Hydrogels. Adv. Healthcare Mater. 2020, 9, 1900977 10.1002/adhm.201900977. PMC7202999 31697028

[ref293] Liu S.; Zhao M.; Zhou Y.; Li L.; Wang C.; Yuan Y.; Li L.; Liao G.; Bresette W.; Chen Y.; Cheng J.; Lu Y.; Liu J. A Self-Assembling Peptide Hydrogel-Based Drug Co-Delivery Platform to Improve Tissue Repair after Ischemia-Reperfusion Injury. Acta Biomater. 2020, 103, 102–114. 10.1016/j.actbio.2019.12.011. 31843715

[ref294] Li R.; Li Y.; Wu Y.; Zhao Y.; Chen H.; Yuan Y.; Xu K.; Zhang H.; Lu Y.; Wang J.; Li X.; Jia X.; Xiao J. Heparin-Poloxamer Thermosensitive Hydrogel Loaded with BFGF and NGF Enhances Peripheral Nerve Regeneration in Diabetic Rats. Biomaterials 2018, 168, 24–37. 10.1016/j.biomaterials.2018.03.044. 29609091 PMC5935004

